# Density functional theory study of the role of benzylic hydrogen atoms in the antioxidant properties of lignans

**DOI:** 10.1038/s41598-018-30860-5

**Published:** 2018-08-17

**Authors:** Quan V. Vo, Pham Cam Nam, Mai Van Bay, Nguyen Minh Thong, Nguyen Duc Cuong, Adam Mechler

**Affiliations:** 1 Department for Management of Science and Technology Development, Ton Duc Thang University, Ho Chi Minh City, Vietnam; 2 Faculty of Applied Sciences, Ton Duc Thang University, Ho Chi Minh City, Vietnam; 3 Department of Chemical Engineering, The University of Da Nang - University of Science and Technology, Da Nang City, Vietnam; 4 Department of Chemistry, The University of Da Nang - University of Education, Da Nang City, Vietnam; 5 The University of Da Nang, Campus in Kon Tum, 704 Phan Dinh Phung, Kon Tum, Vietnam; 6 School of Hospitality and Tourism, Hue University, Hue City, Vietnam; 7 Department of Chemistry and Physics, La Trobe University, Victoria, 3086 Australia

## Abstract

Antioxidants are a diverse group of chemicals with proven health benefits and thus potential preventive medicine and therapeutic applications. While most of these compounds are natural products, determining their mechanism of radical scavenging and common motifs that contribute to antioxidant activity would allow the rational design of novel antioxidants. Here the origins of the antioxidant properties of ten natural products of the lignan family were studied *in silico* by calculating their thermochemical properties by using ROB3LYP/6-311++G(2df,2p)//B3LYP/6-311G(d,p) model chemistry. Three conditions were modelled: gas phase, ethanol and water solvents. The results allowed assigning the antioxidant activity to specific moieties and structural features of these compounds. It was found that the benzylic hydrogen atoms are the most likely to be abstracted to form radicals and hence define antioxidant properties in most of the studied compounds. The results also suggested that the most likely mechanism of HOO^•^ radical scavenging differs by the key moiety: it is hydrogen atom transfer in case the benzylic C-H bonds, however it is proton coupled electron transfer in case of the compounds where O-H bonds are responsible for radical scavenging.

## Introduction

Reactive oxygen species including HO^•^, O_2_^•−^, HOO^•^ are implicated in a range of diseases and medical conditions such as cancer, inflammation and allergies^1,2^. Natural antioxidants play a fundamental role in reducing the effect of oxidants in the environment as well as within the human body^3–6^. Among natural antioxidants, phenolic compounds such as lignans, stilbenes, and flavonoids stand out with their high antioxidant efficiency^5–14^. It was noted that lignans such as guayacasin and isopregomisin exhibit the highest antioxidant activity, exceeding that of the better known flavonoids (e.g. 3′-methoxycalicopterin and 7′-methylsudachitin)^15^ and therefore hold the potential for preventive and/or therapeutical applications in human medicine. Consequently lignans such as secoisolariciresinol diglycoside, enterodiol and enterolactone were shown to inhibit linoleic acid peroxidation in model systems^16^. Since then, many other synthetic and natural lignans (sesaminol triglucoside, sesamol, sesamin, sesamolin, and sesaminol diglucoside from sesame seeds or plants^17–21^; (+)-eudesmin, (+)-magnolin, (7S*, 8S*, 8′S*)-3,4,3′,4′-tetramethoxy-9,7′-dihydroxy-8.8′,7.0.9′-lignan, (+)-epimagnolin A and (+)-fargesin from the *Magnolia denudata* plant^22^; and the synthetic neo- and xanthene lignans^23^) have been confirmed as potent antioxidants and suggested to deliver health benefits.

The *Abies*, *Larix*, *Picea*, *Pinus* and *Tsuga* genera were shown as good sources of natural phenolic antioxidants^24^. Several studies have shown that the species exhibit strong antioxidant properties as they contain numerous potent antioxidants including lignans such as pinoresinol, lignan A, secoisolariciresinol, matairesinol, lariciresinol, nortrachelogenin^24–27^. The antioxidant properties of hydrophilic extracts from several industrially important tree species as well as some isolated compounds were experimentally evaluated by the peroxyl-trapping capacity tests and lipid-peroxidation inhibition (LPI)^24,25,28,29^. Hence these genera are potentially a source of novel, highly potent lignan antioxidants.

It is clear from former experimental and computational studies that the ability of phenolic compounds to scavenge free radicals is due to the presence of phenolic hydroxyl groups, and that it follows either the hydrogen atom transfer (HAT) or the single electron transfer followed by proton transfer (SETPT) mechanism^8,30–34^. Therefore, the bond dissociation energy (BDE) of the X-H (X=C, O) is a key descriptor of the antioxidant properties of lignans. Studies in some typical phenolic compounds including quercetin, isorhamnetin and phloretin showed that the lowest calculated BDE(O-H) values of appropriately 75–77 kcal.mol^−1^ ^35,36^ directly correspond to LPI IC_50_ values of 6.67–12.5 *μ*M^37^. However, there are documented cases of conflict between the lowest calculated BDE(O-H) values and the experimental LPI values; in case of pinoresinol, the lowest BDE(O-H) was 86.0 kcal.mol^−1^ ^38^, but the IC_50_ of the LPI was only 0.06 *μ*M^25^. Moreover, the anti-oxidative activity of (−)-eudesmin (64.1%), which was formed by the methoxylation of pinoresinol, is nearly as high as that of vitamin C (76.4%) in the 2,2′-azinobis(3-ethylbenzothiazoline-6-sulfonic acid test, despite of the fact that there are no hydroxyl groups in this compound^39^. Recent studies showed that the lowest BDE values are not always found for the phenolic hydroxyl groups but for the benzylic C-H^40^. Hence, in predicting antioxidant activity of lignans, the contributions of the benzylic hydrogen atoms should be also investigated.

**Figure 1:**
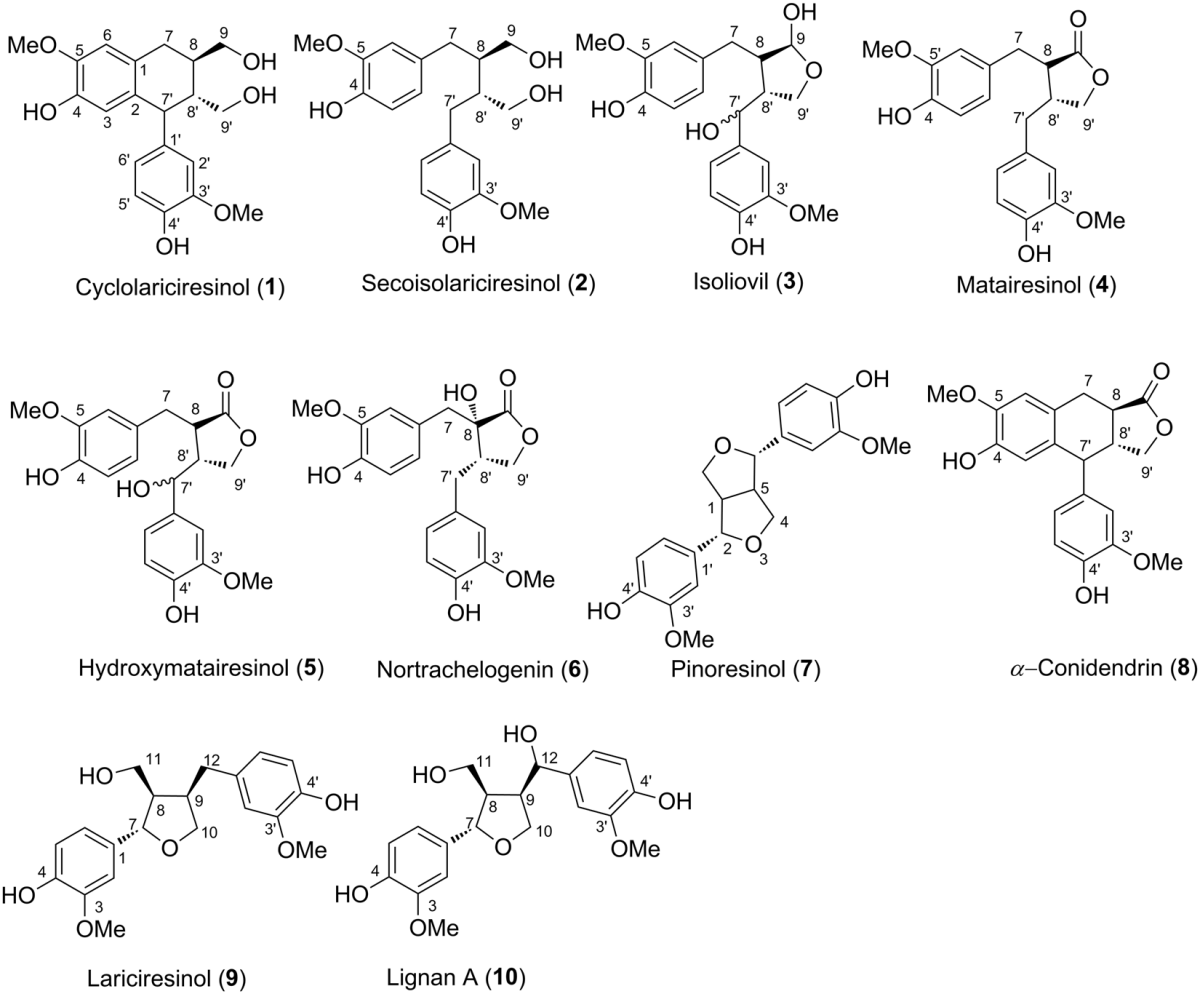
Structures of the lignans (**1**–**10**) studied here for their antioxidant properties.

The purpose of this study is to evaluate the antioxidant capacity of ten phenolic compounds (Fig. 1) of the lignan families identified in the extracts of the *Abies*, *Larix*, *Picea*, *Pinus* and *Tsuga* genera^25^; here antioxidant capacity is defined by the ability of electron transfer and/or H-donation to the free radicals, where H-donating ability is determined to all hydrogen containing moieties. The mechanistic pathway of the radical scavenging activity will be also investigated based on the calculation of thermodynamic parameters. Finally, potential energy surfaces (PES) and natural bond orbital (NBO) will be calculated for evaluating of the mechanism of reactions between the typical antioxidants and the HOO^•^ radical.

## Results and Discussions

### Bond dissociation enthalpies

Previous studies have shown that the BDE value is an important factor to evaluate the antioxidant activity of compounds as it characterizes the ability of donating a hydrogen radical and forming a stable radical of the donor^8,41^. In order to qualitatively identify the lowest BDE values, the BDEs of all possible X-H (X=C, O) bonds breaking in the studied compounds were calculated by the B3LYP/6-31G(d,p) method and presented in Table S1 (SI). With the purpose of accurate prediction, the lowest BDEs of X-H (X=C, O) were then calculated using ROB3LYP/6-311++G (2df,2p)//B3LYP/6-311G(d,p) model chemistry. It was found that, while all the weak O-H bonds are found at the C3 or C4 of the aromatic rings, the lowest BDEs of C_sp3_-H bonds are mostly predicted for the C7′-H (benzylic hydrogen atoms).

Based on the prediction of the weakest bonds for each compound in the gas phase, further BDE calculations have been performed in specific solvents; the other thermochemical parameters and PES have also been calculated for these reactive centers.

### The hydrogen atom transfer (HAT) mechanism

It is well known that HAT mechanism is characterized by the BDE values that correspond to the ability of a XH (X=C, O) moiety to donate its hydrogen atom and consequently form a radical. The lower the BDE of the relevant X-H (X=C, O) bond, the higher antioxidant potency. Thus in this section, the BDE values of the weakest X-H (X=C, O) bonds of each compound were computed in the study environment and shown in Table 1 ^42^. It is clear from the Table 1, the BDE (X-H)s (X=C, O) generally vary from 78.4 to 88.0 kcal.mol^−1^. While the phenolic hydroxyl groups are found as the lowest BDE groups in the compounds **2**, **4**, **6**, and **8**, the C-H (benzylic) bonds have the lowest BDE values at the lignans **1**, **3**, **5**, **7**, **9**, **10** in the range of 79.6–85.0 kcal.mol^−1^. Thus the benzylic hydrogen atoms play an important role in the antioxidant properties of these compounds, that agrees with the results obtained by Papadopoulos *et al*.^40^. That may explain for the low IC_50_ values in the inhibition of lipid peroxidation *in vitro* of the compounds **1** (0.05 *μ*M) and **7** (0.06 *μ*M) despite of the high BDE(O-H) values (the lowest BDE(O-H)s of **1** and **7** are 81.1 and 84.3 kcal.mol^−1^, respectively)^25^. Therefore, we should consider the effect of C7′-H (benzylic) group in studying the antioxidant mechanism for hydroxymatairesinol **5** rather than only focus on phenolic hydroxyl group O4′-H^43^.

**Table 1:** The calculated BDEs in gas phase, water and ethanol solvent at the weakest X-H (X=C, O) bond and the proton dissociation enthalpy (PDE) of the lignans.

Comp.	Name	X-H (X=O, C) position	BDEs (X-H, kcal.mol^−1^)			PDEs (kcal.mol^−1^) Gas phase
			Gas phase	Water	Ethanol	
1	Cyclolariciresinol	O4-H	81.1	83.9	83.2	241.3
		C7′-H	79.8	83.9	83.1	240.1
2	Secoisolariciresinol	O4-H	78.6	81.8	81.1	232.1
3	Isoliovil	O4-H	84.1	86.7	86.1	234.8
		O4′-H	85.8	86.8	86.3	236.5
		C7′-H	82.8	86.6	85.9	233.5
4	Matairesinol	O4-H	85.4	87.2	86.6	230.9
		O4′-H	85.1	84.0	83.6	230.5
5	Hydroxymatairesinol	O4-H	85.7	87.0	86.1	232.1
		O4′-H	85.8	88.0	87.4	232.3
		C7′-H	85.0	84.4	83.8	231.5
6	Nortrachelogenin	O4′-H	78.9	82.3	81.6	229.3
7	Pinoresinol	O4-H	84.3	87.0	86.3	235.7
		C2-H	79.6	82.7	82.1	231.0
8	*α*-Conidendrin	O4-H	84.6	86.8	85.8	233.5
		O4′-H	84.0	86.5	85.8	233.0
		C7′-H	82.0	85.8	85.1	230.9
9	Lariciresinol	O4-H	78.5	82.1	81.4	230.8
		O4′-H	78.4	81.9	81.2	230.7
10	LignanA	O4-H	84.9	84.0	83.6	238.5
		O4′-H	84.6	82.7	82.4	238.2
		C12-H	80.9	83.6	82.9	234.5

On the basis of the gas phase BDE values, the ability of H-donation of the studied compounds follows the sequence: **2** ≈ **6** ≈ **9** > **1** ≈ **7** > **10** > **8** > **3** > **4** ≈ **5**.

For the lignans: secoisolariciresinol **2**, nortrachelogenin **6** and lariciresinol **9** have the lowest BDE values for the O-H moiety at 78.6, 78.9 and 78.4 kcal.mol^−1^, respectively, whilst the lowest BDE values of cyclolariciresinol and pinoresinol belong to C7′-H and C2-H bonds with 79.8 and 79.6 kcal.mol^−1^, respectively.

Typically, the O-H bond that is easiest to dissociate is found at the C4 of the aromatic rings whereas the weakest C-H bond is identified at the C7′-H of the lignans. This can be explained with the electron-withdrawing conjugation effect of the π delocalization in the aromatic the rings. An electron is released from a lone pair of the O atom of the O-H bond into the aromatic ring, leading to increased polarization of the O-H bonds. Upon bond dissociation, the single electron at the O or C atom of the radical is released to the aromatic rings by resonance, *i*.*e*. the formed radical is stabilized. This favors X-H (X=C, O) bond breaking, as reflected in the lower BDEs of these bonds compared to other ones (Fig. 1).

It is clear that the hydrogen-donating ability is affected by the polarity of solvents because of the change of BDE values. Therefore, in this study ethanol and water solvents were used as the environment for calculating of BDEs. Ethanol was chosen because the experimental studies of the antioxidant activity and radical trapping capacity of the lignans were conducted in ethanol^24,25,28^. Water solvent was studied as it is physiologically more relevant if *in vivo* antioxidant activity is concerned^25^. The results obtained for most of the compounds show that there is an increase in the BDEs(X-H, X = O, C) in the solvents in the range of 0.4 to 3.6 kcal.mol^−1^, apart from the compounds **4** (O4′-H), **5** (C7′-H) and **10** (O4-H and O4′-H). However, similar to the gas phase, the compounds **2**, **6** and **9** have the lowest BDE values of O-H in the lignans family with 81.8, 82.3 and 81.9 kcal.mol^−1^ in water and 81.1, 81.6 and 81.2 kcal.mol^−1^ in ethanol, respectively. Surprisingly, the C-H bonds still are the easiest dissociation bonds in the lignans **1**, **3**, **5**, **7**, **9** in studied solvents. On the basis of these calculated values, cyclolariciresinol **1**, secoisolariciresinol **2**, nortrachelogenin **6**, pinoresinol **7** and lariciresinol **9** are the most potential antioxidants.

### The sequential electron transfer proton transfer (SETPT) mechanism

#### Ionization energies

According to SETPT mechanism, ionization of the antioxidant molecule is the first step, and thus IE is used to describe the electron donor ability. The lower the IE value, the easier the electron transfer and the higher the antioxidant activity. The adiabatic IE values were calculated using the same model: ROB3LYP/6-311++G (2df,2p)//B3LYP/6-311G(d,p) in gas phase. The results are shown in Table 2. The sequence of vertical IE values in gas phase is **1** < **10** < **7** < **2** < **3** < **8** < **9** < **5** < **6** < **4**.

**Table 2:** The calculated ionization energy (IE) of the studied compounds.

Comp.	IE (kcal.mol^−1^)		ΔIE (kcal.mol^−1^)*		PDE + IE (kcal.mol^−1^)
	Vertical	Adiabatic	Vertical	Adiabatic	
1	158.9	154.3	−38.0	−39.4	394.3
2	167.0	161.0	−29.9	−29.9	393.1
3	168.3	163.7	−28.6	−28.6	397.2
4	172.5	169.0	−24.4	−24.4	399.5
5	171.3	167.9	−25.6	−25.6	399.4
6	171.8	164.2	−25.1	−25.1	393.5
7	165.1	163.0	−31.8	−31.8	394.1
8	169.0	166.0	−27.9	−27.9	396.5
9	169.3	162.1	−27.6	−27.6	392.8
10	164.7	161.0	−32.2	−32.2	397.4

*ΔIE = IE – IE phenol.

#### Proton dissociation enthalpies

The last step of the SETPT mechanism is the loss of a proton from the cation radical formed in the first step, thus it is characterized by the PDE that determines the thermodynamically preferred X-H (X=C, O) group for deprotonation. The calculated results are given in Table 2.

The easiest deprotonation generally is assigned to the X-H (X=C, O) bond with the lowest BDE that is most likely to break. Among the studied compounds, the lowest PDEs are approximately 230 kcal.mol^−1^ that were calculated for **4**, **8** and **9**. Assuming SETPT mechanism, the antioxidant potential is defined by the combination of PDE and IE; Table 2 shows that, according to this mechanism, compounds **1**, **2**, **6**, **7**, **9** should be the most potential antioxidants, of which compound **9** has the lowest PDE + IE value at 392.8 kcal.mol^−1^. Hence this compound would be the best antioxidant irrespective of whether the reaction follows the HAT or the SETPT mechanism. Comparison with the calculated BDE and IE of phenol (87.7 kcal.mol^−1^ and 196.9 kcal.mol^−1^, respectively) at ROB3LYP/6-311++G(2df,2p)//B3LYP/6-311G(d,p), the ΔBDEs and ΔIEs of the studied compounds are in the range of −9.3 to 0.3 kcal.mol^−1^ and −24.4 to −39.0 kcal.mol^−1^, respectively. Thus the HAT mechanism appears to be the main pathway for the lignans^44^.

### The sequential proton loss electron transfer (SPLET) mechanism

#### Proton affinities

The sequential proton loss electron transfer (SPLET) mechanism starts with the dissociation of the acidic moiety, which can be characterized by the proton affinity; this is followed by an electron transfer to the free radical, at a cost of the electron transfer energy. Lower PA is characteristic of higher antioxidant capacity *via* this mechanism.

As described above, the PAs values of the compounds were first evaluated by the B3LYP/6-31G(d, p) method (Table S2, SI), then the lowest PAs of X-H (X=C, O) were calculated using ROB3LYP/6-311++G (2df,2p)//B3LYP/6-311G(d,p) model chemistry. The results are shown in Table 3. As can be seen from the Table 3, the PA values of O-H bonds are lower than those of C-H bonds. For example, the lowest PA values of O-H bonds in gas phase at **1** and **7** are 341.6 and 342.6 kcal.mol^−1^, respectively, whereas for C-H bonds these are 363.4 and 377.5 kcal.mol^−1^, respectively. Thus, the C-H bonds are not favored for SPLET mechanism. The compound **5** has the lowest PA value at 335.3 kcal.mol^−1^, followed by three lignans, **6** (O4′-H), **2** and **9** (O4-H) with the values of 335.7, 338.1 and 338.8 kcal.mol^−1^, respectively. It was found that the solvents (both water and ethanol) lead to a slight decrease in the PA values. The PA (**5** (O4′-H)) decreases from 335.3 kcal.mol^−1^ in the gas phase to 299.0 and 300.2 kcal mol^−1^ in water and ethanol, respectively. Similarly, the PA (**6** (O4′-H)) values are 335.7, 294.5 and 295.8 kcal.mol^−1^ in the gas phase, water and ethanol, respectively. This can be explained by the high solvation enthalpy of the proton in polar solvents. Thus, the PA values obtained in the gas phase are higher than the ones obtained in water and ethanol solvents. The results agree with experimental studies^45–48^.

**Table 3:** The calculated PAs and ETEs of the studied lignans.

Comp.	X-H (X=C, O) position	PAs (kcal.mol^−1^)			ETEs (kcal.mol^−1^)		
		Gas phase	Water	Ethanol	Gas phase	Water	Ethanol
1	O4′-H	341.6	296.3	297.8	53.9	100.0	89.5
	O4-H	243.7	298.4	299.9	52.9	96.7	95.2
	C7′-H	363.4	230.4	331.4	30.8	64.8	63.7
2	O4-H	338.1	295.7	297.0	55.0	97.4	96.1
3	O4-H	344.7	299.9	301.4	53.9	98.1	96.7
	O4′-H	342.0	298.5	299.9	58.2	99.7	98.4
	C7′-H	375.7	337.9	339.1	21.6	60.0	58.7
4	O4-H	339.9	300.0	301.4	60.0	98.5	97.2
	O4′-H	342.4	300.8	302.2	57.1	94.6	93.4
5	O4-H	339.3	303.3	304.5	60.9	95.1	94.4
	O4′-H	335.3	299.0	300.2	64.4	100.3	99.2
	C7′-H	373.6	338.7	339.8	25.9	60.7	59.6
6	O4′-H	335.7	294.5	295.8	57.7	99.1	97.8
7	O4-H	342.6	297.9	299.3	56.1	100.4	99.0
	C2-H	377.5	337.7	339.0	16.6	56.4	55.1
8	O4-H	342.8	299.7	301.2	56.3	98.4	97.0
	O4′-H	343.7	303.1	304.4	54.8	94.7	93.4
	C7′-H	364.7	331.1	332.2	31.8	66.0	64.9
9	O4-H	338.8	293.9	295.3	54.1	99.5	98.0
	O4′-H	342.7	295.6	297.1	51.2	97.6	96.1
10	O4-H	348.4	297.5	297.1	50.9	97.9	98.4
	O4′-H	342.8	299.6	301.0	56.3	94.5	93.3
	O11-H	341.0	300.6	301.9	75.0	115.9	114.6
	C12-H	374.3	338.4	339.5	21.0	56.6	55.5

#### Electron transfer enthalpies

The ETE is a key descriptor of the last step of the SPLET mechanism. It is clear from Table 3 that the IEs (data in Table 2) are much higher than the ETE values in the gas. Therefore, the single electron transfer process from the neutral form is not as preferable as from the anionic form. This result agrees with the previous studies^45,49–52^. It was found that the following electron transfer process does not favor in the polar solvents. In fact, there is a significant increase in ETE values in the studied solvents (both water and ethanol) compared with those obtained in the gas phase. On the calculated PA and ETE values of both step of the SPLET mechanism, **9** has the highest antioxidant activity with the total PA + ETE value at around 393 kcal.mol^−1^ in all of the studied environment. That agrees with our results in HAT and SETPT mechanism investigations.

### The reaction with HOO• radical: potential energy surfaces (PES)

**Figure 2:**
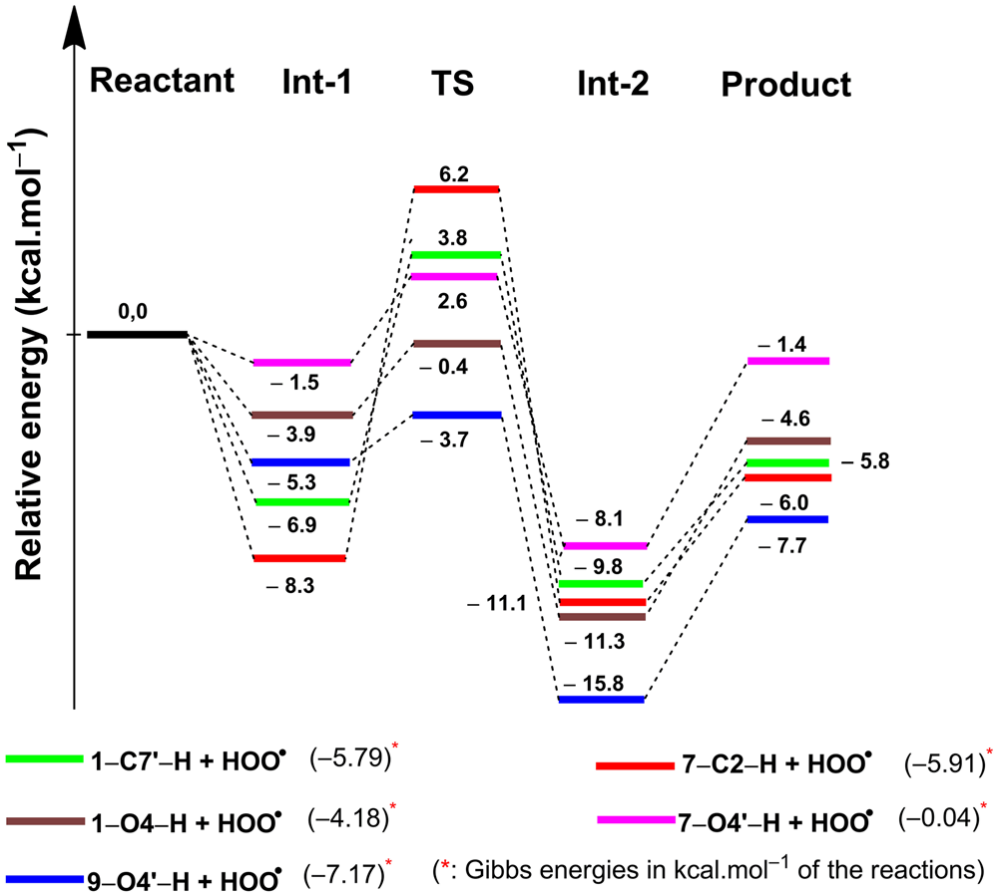
PES of reaction between the selected phenolic compounds and HOO^•^.

The antioxidant capacity of the phenolic compounds with the lowest BDE can be expressed by constructing the PES of their reaction with a ROO^**•**^ radical. Thus the PESs of the reactions of the most potential antioxidant compounds **9** and the lowest BDE(C-H) compounds **1**, **7** with HOO^**•**^ radical were investigated. The optimized structures and the energies of the reactants (R), the transition states (TS), the intermediates (Int) and the products (P) of each compound were calculated using the same computational method. The IRCs were calculated to ensure that each transition state connects to the expected reactant and product and shown in Fig. S1 (SI). All optimized TS structures were shown in Fig. S2 (SI) and the PESs were built and displayed in Fig. 2.

Based on the calculations, the reaction path for the hydrogen abstraction can be described as follows:

$${\bf{R}}\to \,{\bf{Int}} \mbox{-} {\bf{1}}\to {\bf{TS}}\to \,{\bf{Int}} \mbox{-} {\bf{2}}\to {\bf{P}}$$

In the structures of the transition states, the H atom of the X-H (X=C, O) bond is located nearly midway between the two O atoms. The H···OOH and X···H (X=C, O) distances are in the range of 1.148–1.298 Å and 1.115–1.301 Å, respectively. The X···H···O (X=C, O) angles are in the range of 161.3–187.3° (Fig. S2, SI). The energies of the studied TSs are only −0.4 and −3.7 kcal.mol^−1^ for **1**-O4-H-OOH and **9**-O4′-H-OOH, respectively, while for others are in the range of 2.6–6.2 kcal.mol^−1^ (Fig. 2). The results suggest that compound **9** has the highest antioxidant activity due to the lowest TS energy, in a good agreement with the above-described results. These phenolic compounds can easily transfer the hydrogens of either of the benzylic groups or phenyl hydroxyl groups to the peroxide radical species, forming the neutral compound (HOOH) and an unreactive radical (ArX^•^). On the basis of the calculated Gibbs free energies given in the last column of Fig. 2, these hydrogen abstraction reactions will be spontaneous. Noticing that, Gibbs free energies of the reactions of the benzylic hydrogen at **7** are more negative than these for the hydrogen atom of phenolic hydroxyl group (ΔG (**7**-C2-H + HOO^•^) = −5.91 kcal.mol^−1^ compared to −0.04 kcal.mol^−1^ for the **7**-O4′-H + HOO^•^ reaction). Thus the hydrogen abstraction reaction of the **7**-C2-H has a higher priority than that of **7**-O4′-H.

### Natural bond orbital (NBO)

**Figure 3:**
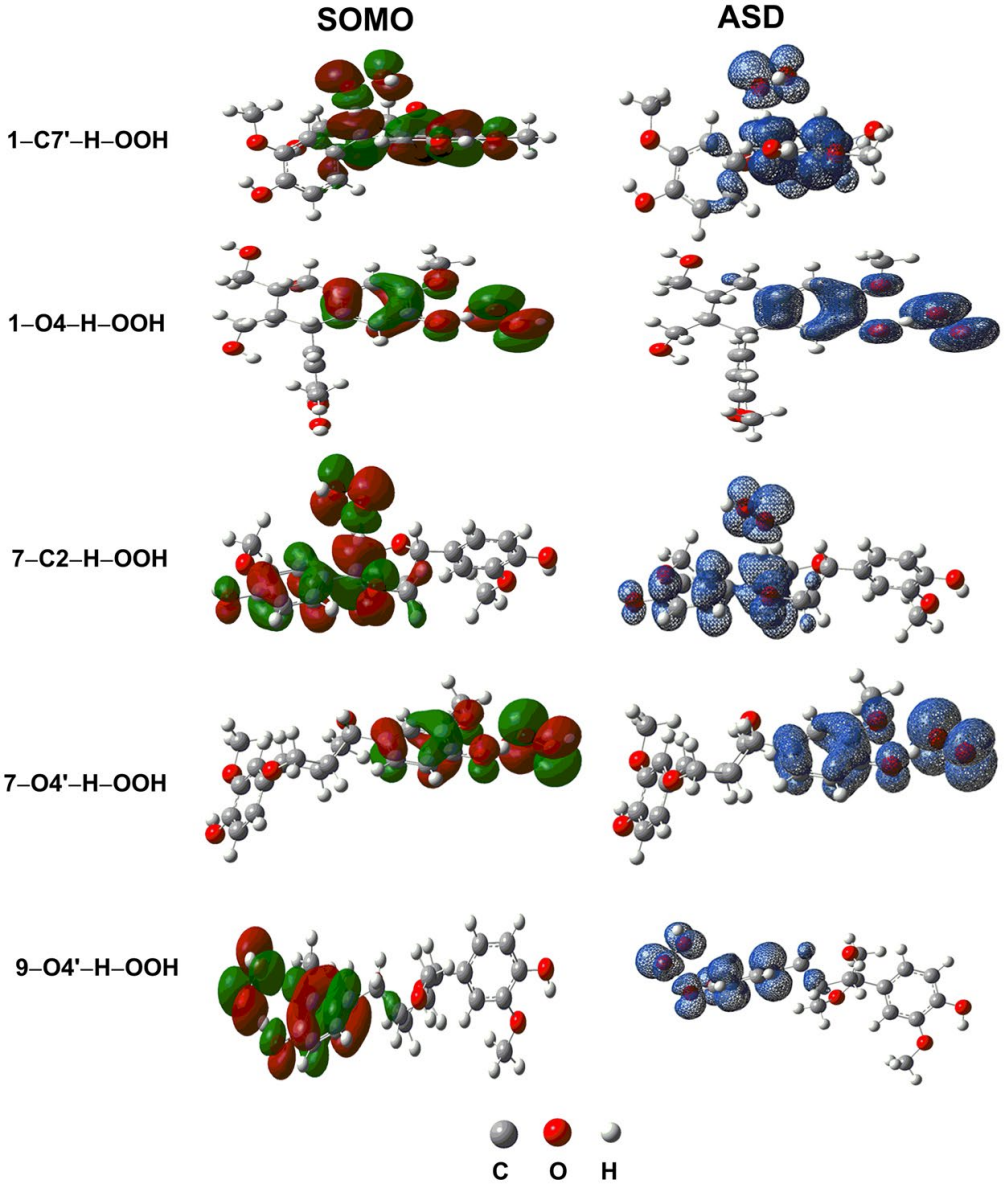
SOMO densities surface and ASD of the transition states of the reactions.

In order to gain further insights of the antioxidant mechanism, natural bond population (NBP) charge, the atomic spin densities (ASD) and singly-occupied molecular orbitals (SOMO) of the transition states of the studied compounds were also determined as shown in Table 4 and Fig. 3. Analysis of SOMO shows that a significant atomic orbital density oriented along the O1···H···X (X=C, O) transition vector is observed at the TSs of **1**-C7′-H-OOH, and **7**-C2-H-OOH but that is not found in the transition states of **1**-O4-H-OOH, **7**-O4′-H-OOH and **9**-O4′-H-OOH (Fig. 3). In the SOMO of the **1**-O4-H-OOH, **7**-O4′-H-OOH and **9**-O4′-H-OOH TSs, the atomic orbital density is distributed on both sides of the transition vector and is close to being planar. Furthermore, natural bond analysis shows that the stabilization energy values (E(2)) of the donor LP(3)O1 and the acceptor σ*(1)X-H, which are favored in the HAT process^53^, are found in the transition states of **1**-C7′-H-OOH, **7**-C2-H-OOH at 53.1 and 45.2, kcal.mol^−1^, respectively (Table 4). Surprisingly, these values are too small for the TSs of **1**-O4-H-OOH and **7**-O4′-H-OOH (<1.0 kcal.mol^−1^). Instead, high stabilization energy, that is consistent with the hydrogen bond in the proton coupled electron transfer (PCET) mechanism^53,54^, is found for the donor LP(3)O1 and the acceptor LP*(1)H (X-H) of **1**-O4-H-OOH, **7**-O4′-H-OOH and **9**-O4′-H-OOH at 99.6, 98.7 and 81.4 kcal.mol^−1^, respectively. That for the donor LP(3)O(O-H) and the acceptor LP*(1)H (O-H) is 131.4, 127.9 and 151.2 kcal.mol^−1^, respectively (Table 4). Thus it suggests that the HAT mechanism is most likely for the H-atom abstraction of the C-H bonds, while the PCET mechanism is favored for the H-atom abstraction of O-H bonds.

**Table 4:** Natural bond analysis of transition states of the reactions.

Reactions	Donor NBO (i)	Acceptor NBO (j)	E(2) (kcal.mol^−1^)
**1**-C7′-H + HOO^•^	LP(3)O1	σ*(1)C7′-H	53.1
**1**-O4-H + HOO^•^	LP(3)O1	LP*(1)H	99.6
	LP(3)O4	LP*(1)H	131.4
**7**-C2-H + HOO^•^	LP(3)O1	σ*(1)C2-H	45.2
**7**-O4′-H + HOO^•^	LP(3)O1	LP*(1)H	98.7
	LP(3)O4′	LP*(1)H	127.9
**9**-O4′-H + HOO^•^	LP(3)O1	LP*(1)H	81.4
	LP(3)O4′	LP*(1)H	151.2

### Theoretical and computational methods

In this work, all computational calculations were performed using the Gaussian 09 suite of programs^55^. The B3LYP/6-311G(d,p) level of theory was used for optimizing the geometry and determining the vibrational frequencies of each neutral compound and the related radicals, cationic radicals and anions^56,57^. The ROB3LYP/6-311++G(2df,2p) method was then used to calculate the single point electronic energies^42^. For the species that have multiple conformers, all of these were investigated and the conformer with the lowest electronic energy and possible intermolecular hydrogen bonds was included in the analysis^58–60^.The potential energy surfaces of the reaction between the selected lignans and the HOO^•^ radical were investigated based on the calculation of the transition states, intermediates, products and the intrinsic reaction coordinate at the same level of theory. The integral equation formalism of polarizable continuum model (IEF-PCM) was used to treat implicitly of the solvents at the same level of theory as in the gas phase^61,62^.

In order to determine the mechanistic pathway of the radical scavenging process, thermochemical properties were used, by assessing the energetics of the determining step of each pathway. The literature recognizes three common mechanisms of antioxidant activity^8,41^. In the hydrogen atom transfer (HAT) mechanism, the first step is the homolytic bond breakage in an appropriate moiety to yield a hydrogen radical, which then reacts with the free radical species; here the bond dissociation energy of the R-H moiety determines the enthalpy of the first step.

1 $${\rm{R}}-{\rm{H}}\to {{\rm{R}}}^{\bullet }+{{\rm{H}}}^{\bullet }({\rm{BDE}}({\rm{R}}-{\rm{H}}))$$

In the “Single electron transfer followed by proton transfer” (SETPT) mechanism the first step is electron loss to form a radical cation, characterized by the ionization energy, followed by a deprotonation step that is described with the proton dissociation energy.

2 $${\rm{R}}-{\rm{H}}\to {{\rm{RH}}}^{+\bullet }+{{\rm{e}}}^{-}\,({\rm{IE}})$$

3 $${{\rm{RH}}}^{+\bullet }\to {{\rm{R}}}^{\bullet }+{{\rm{H}}}^{+}\,({\rm{PDE}})$$

The third mechanism, “Sequential proton loss electron transfer” (SPLET), starts with the dissociation of the acidic moiety, which can be characterized by the proton affinity; this is followed by an electron transfer to the free radical, at a cost of the electron transfer energy

4 $${\rm{R}}-{\rm{H}}\to {{\rm{R}}}^{-}+{{\rm{H}}}^{+}\,({\rm{PA}})$$

5 $${{\rm{R}}}^{-}\to {{\rm{R}}}^{\bullet }+{{\rm{e}}}^{-}\,({\rm{ETE}})$$

Thus the reaction enthalpies of the individual steps in the above described mechanisms of antioxidant activity in gas phase (at 298.15 K and 1 atm) are calculated as follows^41,46,49^:

6 $${\rm{BDE}}=H({{\rm{R}}}^{\bullet })+H({{\rm{H}}}^{\bullet })-H({\rm{R}}-{\rm{H}})$$

7 $${\rm{IE}}=H({{\rm{RH}}}^{\bullet +})+H({{\rm{e}}}^{-})-H({\rm{R}}-{\rm{H}})$$

8 $${\rm{PDE}}=H({{\rm{R}}}^{\bullet })+H({{\rm{H}}}^{+})-H({{\rm{R}}}^{\bullet +})$$

9 $${\rm{PA}}=H({{\rm{R}}}^{-})+H({{\rm{H}}}^{+})-H({\rm{R}}-{\rm{H}})$$

10 $${\rm{ETE}}=H({{\rm{R}}}^{\bullet })+H({{\rm{e}}}^{-})-H({{\rm{R}}}^{-})$$

In the gas phase, the enthalpy of hydrogen atom was of −0.5 Hartree and for other environment, the enthalpy of hydrogen atom was calculated at the same method. The calculated enthalpies of the electron (e^−^) and proton (H^+^) were taken from the literature^41,63–65^. Vibrational frequencies obtained at the B3LYP/6-31G(d,p) and B3LYP/6-311G(d,p) levels were scaled by a factor of 0.9611 and 0.9669, respectively^66,67^.

## Conclusions

The antioxidant activities of ten lignans in gas phase, ethanol and water solvents has been successfully evaluated via their thermochemical properties by using the ROB3LYP/6-311++G(2df,2p)//B3LYP/6-311G(d,p) calculation method. The BDE values of the X-H (X=C, O) moieties of the studied compounds were all found in the range of 78.4–88.0 kcal.mol^−1^. Cyclolariciresinol, secoisolariciresinol, nortrachelogenin, pinoresinol and lariciresinol are predicted to be the most potential antioxidants, especially lariciresinol that was found to be the best antioxidant compound under all of the studied conditions. The results suggest that the benzylic hydrogen atoms play an important role in antioxidant properties of lignans alongside the hydrogen atoms of phenolic hydroxyl groups. Modelling the HOO^•^ radical scavenging mechanism of cyclolariciresinol, pinoresinol and lariciresinol suggests that the hydrogen atom transfer mechanism is most likely for the H-atom abstraction of the C-H bonds, while the proton coupled electron transfer mechanism is favored for the H-atom abstraction of O-H bonds.

## Electronic supplementary material

Supplementary Information
