# Author Correction: Density functional theory study of the role of benzylic hydrogen atoms in the antioxidant properties of lignans

**DOI:** 10.1038/s41598-019-40317-y

**Published:** 2019-03-12

**Authors:** Quan V. Vo, Pham Cam Nam, Mai Van Bay, Nguyen Minh Thong, Nguyen Duc Cuong, Adam Mechler

**Affiliations:** 1 Department for Management of Science and Technology Development, Ton Duc Thang University, Ho Chi Minh City, Vietnam; 2 Faculty of Applied Sciences, Ton Duc Thang University, Ho Chi Minh City, Vietnam; 3 Department of Chemical Engineering, The University of Da Nang - University of Science and Technology, Da Nang City, Vietnam; 4 Department of Chemistry, The University of Da Nang - University of Education, Da Nang City, Vietnam; 5 The University of Da Nang, Campus in Kon Tum, 704 Phan Dinh Phung, Kon Tum, Vietnam; 6 School of Hospitality and Tourism, Hue University, Hue City, Vietnam; 7 Department of Chemistry and Physics, La Trobe University, Victoria, 3086 Australia

Correction to: *Scientific Reports* 10.1038/s41598-018-30860-5, published online 17 August 2018

This Article contains errors in the Acknowledgements section.

“This work was supported by Vietnam National Foundation for Science and Technology Development (NAFOSTED) [grant number “104.06–2016.03” (3/2017/104/HĐTN)].”

should read:

“This research is funded by Vietnam National Foundation for Science and Technology Development (NAFOSTED) under grant number 104.06–2016.03.”

