# Supplementary material for: Density functional theory study of the role of benzylic hydrogen atoms in the antioxidant properties of lignans
Source: Sci Rep. 2018 Aug 17;8:12361. doi: 10.1038/s41598-018-30860-5 (PMC6098005; doi:10.1038/s41598-018-30860-5)
Supplement: Supplementary file 1 — Supplementary Information [file 41598_2018_30860_MOESM1_ESM.pdf]

Supplementary information (SI)

Density functional theory study of the role of benzylic hydrogen atoms in the antioxidant properties of lignans

Quan Van Vo,[1,2*], Pham Cam Nam,[3*] Mai Van Bay,[4] Nguyen Minh Thong [5], Nguyen Duc Cuong,[6] Adam Mechler[7]

1Department for Management of Science and Technology Development, Ton Duc Thang University, Ho Chi Minh City, Vietnam

[2]Faculty of Applied Sciences, Ton Duc Thang University, Ho Chi Minh City, Vietnam

[3]Department of Chemical Engineering, The University of Da Nang - University of Science and Technology, Vietnam

[4]Department of Chemistry, The University of Da Nang - University of Education, Vietnam

[5]The University of Danang, Campus in Kon Tum, 704 Phan Dinh Phung, Kon Tum, Vietnam

[6] University of Sciences- School of Hospitality and Tourism, Hue University, Hue City, Vietnam

[7]Department of Chemistry and Physics, La Trobe University, Victoria 3086, Australia

*Corresponding author: vovanquan@tdt.edu.vn; pcnam@dut.udn.vn

List of supporting information:

Table of Contents

Table S1: BDE values of the X–H (X = O, C) bond breaking of different compounds in the gas phase using B3LYP/6-31g(d,p) and ROB3LYP/6-311G++G(2df,2p)//B3LYP/6-311G(d,p) methods. 3

Table S2: Proton affinities (PA) of the X–H (X = O, C) bond breaking in the gas phase calculated using B3LYP/6-31g(d,p) and ROB3LYP/6-311G++G(2df,2p)//B3LYP/6-311G(d,p) methods. 6

Table S3: Cartesian coordinates and molecular enthalpies of all parent molecules, radicals and anions optimized at B3LYP/6-311G(d,p) level of theory in the gas phase, in water and in ethanol 9

Table S4: Cartesian coordinates of all of the Int, TS of the selective compounds and HOO• optimized at B3LYP/6-311G(d,p) level of theory in the gas phase........ 96

Figure S1: IRC plots for all transition states related to the reaction of HOO• radical with cyclolariciresinol 1, pinoresinol 7 and lariciresinol 9 at B3LYP/6-311G(d,p) level of theory in the gas phase........ 115

Figure S2. Optimized geometries of TS for the H abstraction channel of reaction between the selected phenolic compounds and the HOO• radical at the B3LYP/6-311G(d,p) level of theory (distances are given in angstroms) 118

Table S1: BDE values of the X–H (X = O, C) bond breaking of different compounds in the gas phase using B3LYP/6-31g(d,p) and ROB3LYP/6-311G++G(2df,2p)//B3LYP/6-311G(d,p) methods.

Comp. No	X-H (X = O, C) position	BDE (kcal/mol)	
		B3LYP/6-31G(d,p)	ROB3LYP/6-311G++G(2df,2p)//B3LYP/6-311G(d,p)
1	Cyclolariciresinol		
	O4-H	**77.7**	81.1
	O4'-H	78.1	
	C7-H	79.6	
	C8-H	90.0	
	C8'-H	84.3	
	C7'-H	**77.2**	79.8
	C9-H	88.4	
	C9'-H	86.5	
	O9-H	96.2	
	O9'-H	93.9	
	5-OC-H(CH_3)	93.5	
	Secoisolariciresinol		
2	O4-H	**74.6**	78.6
	C7-H	83.5	
	C8-H	89.4	
	C9-H	91.4	
	5-OC-H (of OCH_3)	97.2	
	O9-H	96.2	
	Isoliovil		
3	5-OC-H (of OCH_3)	97.0	
	O4-H	**81.7**	84.1
	3'-OC-H (of OCH_3)	97.0	
	O4'-H	**82.1**	85.8
	C7-H	84.2	
	C7'-H	**80.2**	82.8
	C8-H	89.7	
	C8'-H	89.1	
	C9-H	94.8	
	C9'-H	93.2	
	O7'-H	101.1	

	O9-H	96.7	
	Matairesinol		
**4**	5-OC-H (of OCH_3)	97.6	
	O4-H	**81.3**	85.4
	3’-OC-H (of OCH_3)	97.5	
	O4’-H	**81.9**	85.1
	C7-H	87.1	
	C7'-H	86.3	
	C8-H	88.0	
	C8'-H	94.0	
	C9'-H	95.0	
	Hydroxymatairesinol		
**5**	5-OC-H (of OCH_3)	97.0	
	O4-H	**82.1**	85.7
	3’-OC-H (of OCH_3)	97.6	
	O4’-H	**83.1**	85.8
	C7-H	84.6	
	C7'-H	**82.7**	85.0
	C8-H	84.5	
	C8'-H	93.9	
	C9'-H	95.1	
	O7'-H	100.6	
	Nortrachelogenin		
**6**	O4-H	82.2	
	5-OC-H (of OCH_3)	97.3	
	3’-OC-H (of OCH_3)	97.1	
	O4’-H	**75.0**	78.9
	C7-H	88.2	
	C7'-H	85.5	
	C8'-H		
	C9'-H	94.9	
	O8-H	94.6	
	Pinoresinol		
**7**	5-OC-H (of OCH_3)	97.5	
	O4’-H	**78.9**	84.3
	C1-H	97.1	
	C4-H	91.6	
	C2	**78.1**	79.6

	α-Conidendrin		
**8**	5-OC-H (of OCH_3)	97.3	
	O4-H	**80.6**	84.6
	3'-OC-H (of OCH_3)	97.1	
	O4'-H	**80.1**	84.0
	C7-H	85.4	
	C8-H	86.5	
	C8'-H	92.9	
	C7'-H	**80.0**	82.0
	C9'-H	96.4	
	Lariciresinol		
9	5-OC-H (of OCH_3)	97.5	
	O4-H	**74.5**	78.5
	3'-OC-H (of OCH_3)	96.9	
	O4'-H	**74.4**	78.4
	C7-H	79.7	
	C8-H	91.5	
	C9-H	91.3	
	C10-H	92.9	
	C11-H	92.3	
	C12-H	85.6	
	Lignan-A		
**10**	5-OC-H (of OCH_3)	97.0	
	O4-H	**81.4**	84.9
	3'-OC-H (of OCH_3)	97.1	
	O4'-H	**80.0**	84.6
	C7-H	81.3	
	C8-H	92.4	
	C9-H	94.0	
	C10-H	91.0	
	C11-H	91.9	
	C12-H	**78.0**	80.9
	O11-H	98.0	
	O12-H	97.7	

Table S2: Proton affinities (PA) of the X–H (X = O, C) bond breaking in the gas phase calculated using B3LYP/6-31g(d,p) and ROB3LYP/6-311G++G(2df,2p)//B3LYP/6-311G(d,p) methods.

Comp. No	X-H (X = O, C) position	PAs kcal/mol	
		B3LYP/6-31G(d,p)	ROB3LYP/6-311G++G(2df,2p)//B3LYP/6-311G(d,p)
**1**	Cyclolariciresinol		
	O4-H	352.8	
	O4'-H	**350.0**	341.6
	C7-H	380.1	
	C8-H	396.4	
	C8'-H	382.6	
	C7'-H	371.1	
	C9-H	410.4	
	C9'-H	363.1	
	O9-H	352.3	
	O9'-H	352.3	
	5-OC-H(CH_3)	401.7	
**2**	Secoisolariciresinol		
	O4-H	**347.8**	338.1
	C7-H	375.8	
	C8-H	361.6	
	C9-H	406.1	
	5-OC-H (of OCH_3)	415.2	
	O9-H	369.9	
**3**	Isoliovil		
	5-OC-H (of OCH_3)	357.4	
	O4-H	354.6	
	3'-OC-H (of OCH_3)	399.6	
	O4'-H	**351.9**	342.0
	C7-H	384.7	
	C7'-H	387.2	
	C8-H	389.7	
	C8'-H	386.6	
	C9-H	401.6	
	C9'-H	357.4	
	O7'-H	371.7	
	O9-H	364.5	
**4**	Matairesinol		
	5-OC-H (of OCH_3)	349	

	O4-H	**349.3**	339.9
	3'-OC-H (of OCH_3)	350.6	
	O4'-H	351.5	
	C7-H	383.8	
	C7'-H	377.6	
	C8-H	370.5	
	C8'-H	390.4	
	C9'-H	401.6	
**5**	Hydroxymatairesinol		
	5-OC-H (of OCH_3)	385.6	
	O4-H	347.3	
	3'-OC-H (of OCH_3)	388.1	
	O4'-H	**344.6**	342.4
	C7-H	379.3	
	C7'-H	383.1	
	C8-H	359.2	
	C8'-H	338.6	
	C9'-H	399.0	
	O7'-H	353.1	
**6**	Nortrachelogenin		
	O4-H	351.2	
	5-OC-H (of OCH_3)	401.5	
	3'-OC-H (of OCH_3)	416.2	
	O4'-H	**345.2**	335.7
	C7-H	374.0	
	C7'-H	364.0	
	C8'-H	382.3	
	C9'-H	393.6	
	O8-H	356.5	
**7**	Pinoresinol		
	5-OC-H (of OCH_3)	403.8	
	O4'-H	**352.4**	342.6
	C1-H	393.7	
	C4-H	387.0	
	C2	390.1	
**8**	*α*-Conidendrin		
	5-OC-H (of OCH_3)	405.0	
	O4-H	**352.4**	342.8
	3'-OC-H (of OCH_3)	404.2	

	O4'-H	**353.2**	343.7
	C7-H	386.1	
	C8-H	370.6	
	C8'-H	358.1	
	C7'-H	373.4	
	C9'-H	399.8	
9	Lariciresinol		
	5-OC-H (of OCH_3)	419.8	
	O4-H	**349.2**	338.8
	3'-OC-H (of OCH_3)	418.9	
	O4'-H	351.4	
	C7-H	386.0	
	C8-H	393.4	
	C9-H	392.0	
	C10-H	403.3	
	C11-H	406.2	
	C12-H	371.5	
**10**	Lignan-A		
	5-OC-H (of OCH_3)	403.3	
	O4-H	359.8	
	3'-OC-H (of OCH_3)	405.4	
	O4'-H	**352.6**	342.8
	C7-H	387.0	
	C8-H	382.2	
	C9-H	385.0	
	C10-H	397.9	
	C11-H	364.0	
	C12-H	384.9	
	O11-H	**348.4**	341.0
	O12-H	364.0	

Table S3: Cartesian coordinates and molecular enthalpies of all parent molecules, radicals and anions optimized at B3LYP/6-311G(d,p) level of theory in the gas phase, in water and in ethanol

Name of compound (1)				Cyclolariciresinol
Cartesian Coordinates optimized at B3LYP/6-311G(d,p)				
C	1.12807200	-1.55529500	0.47519900	
C	2.27686700	-2.32146000	0.41196700	
C	3.50069200	-1.70566300	0.09072200	
C	3.54028700	-0.34223500	-0.14592700	
C	-0.14208800	0.63667200	0.36728700	
C	2.37242800	0.43684700	-0.07373700	
C	1.15348400	-0.17232600	0.23040900	
C	1.45868400	4.19767500	-0.15360600	
H	2.52955600	4.43852800	-0.19254600	
H	1.04104100	4.36846400	-1.15700100	
C	-1.27972300	2.90784300	0.08826400	
H	-1.03882700	3.97258100	0.08975900	
H	-1.64835000	2.65807400	1.09429100	
C	1.29497900	2.72659400	0.24052500	
C	-0.02919000	2.07278000	-0.22367500	
H	1.34802700	2.69500300	1.33664200	
H	0.01206200	1.99870900	-1.31817000	
O	0.81544100	5.02734700	0.81888500	
H	0.90025700	5.94224000	0.53412900	
O	-2.26692400	2.62268900	-0.90667400	
H	-3.10278600	2.99423800	-0.61177100	
O	2.22731900	-3.66193500	0.65643600	
H	3.12539200	-4.00195300	0.55033700	
H	4.47876900	0.14327700	-0.38577000	
C	2.47273300	1.92353700	-0.32847500	
H	3.40914300	2.30086400	0.09697200	
H	2.53520900	2.10965800	-1.41126200	
H	-0.30511700	0.77594500	1.44577900	
C	5.84849500	-2.05047000	-0.27330200	
H	5.85155400	-1.60406900	-1.27374300	
H	6.53392000	-2.89634800	-0.25286000	
H	6.17240700	-1.30301700	0.45923000	
O	4.56883100	-2.57151100	0.05388000	
H	0.19653100	-2.05620400	0.70709400	
C	-1.35307800	-0.12699800	-0.15686800	
C	-1.44018100	-0.52969400	-1.48880200	
C	-2.40839800	-0.44667900	0.70723700	
C	-2.55439200	-1.21776500	-1.96132000	
H	-0.63019500	-0.30556000	-2.17298000	
C	-3.52424500	-1.13427300	0.24067900	
C	-3.60490700	-1.52193300	-1.10589800	
H	-2.62658000	-1.52891800	-2.99670600	
O	-4.69769600	-2.19623100	-1.56564600	
H	-5.29263400	-2.31331300	-0.81345200	
H	-2.34712500	-0.15342800	1.74807900	
C	-4.61879100	-1.20543000	2.37928500	
H	-3.78124500	-1.69328900	2.88979900	
H	-5.55726000	-1.59378300	2.77231900	

H -4.57165600 -0.12513500 2.55640800<br>O -4.61978000 -1.50197700 0.99132600		
Frequency and Energy at B3LYP/6-311G(d,p)in gas phase		
Zero-point correction= 0.412443 (Hartree/Particle)<br>Thermal correction to Energy= 0.438162<br>Thermal correction to Enthalpy= 0.439106<br>Thermal correction to Gibbs Free Energy= 0.355886<br>Sum of electronic and zero-point Energies= -1227.791466<br>Sum of electronic and thermal Energies= -1227.765747<br>Sum of electronic and thermal Enthalpies= -1227.764803<br>Sum of electronic and thermal Free Energies= -1227.848022		
Energy at ROB3LYP/6-311++G(2df,2p): in gas phase		HF=-1229.5113613
Energy at ROB3LYP/6-311++G(2df,2p): in H2O		HF=-1228.3286184
Energy at ROB3LYP/6-311++G(2df,2p): in ETHANOL		HF=-1228.327595
**Name of radical**	**Cyclolariciresinol-A4**	
Cartesian Coordinates optimized at B3LYP/6-311G(d,p)		
0 2<br>C 1.29727500 -1.40268900 0.35492100<br>C 2.53486000 -2.14663800 0.30238600<br>C 3.74135500 -1.35862900 0.02714400<br>C 3.63355400 0.01697400 -0.14925300<br>C -0.13148900 0.68087700 0.32504500<br>C 2.41264400 0.68380700 -0.08076300<br>C 1.21142300 -0.04445600 0.17584000<br>C 1.18752800 4.35619900 -0.13847300<br>H 2.23551600 4.68547700 -0.12896700<br>H 0.80202600 4.50004700 -1.15876200<br>C -1.45645000 2.85355400 0.02908200<br>H -1.31896600 3.92925100 -0.03981900<br>H -1.77093600 2.64487200 1.06341800<br>C 1.12945900 2.87267900 0.23898200<br>C -0.12735200 2.12328700 -0.26531300<br>H 1.15431000 2.83579200 1.33586000<br>H -0.05483200 2.06104500 -1.35950300<br>O 0.43428400 5.11231900 0.81032100<br>H 0.42374400 6.03090400 0.52499200<br>O -2.49033500 2.54270000 -0.89735000<br>H -2.67269800 1.59950200 -0.83642600<br>O 2.56340700 -3.38530400 0.48636500<br>H 4.54600200 0.57003600 -0.34515100<br>C 2.38329000 2.17364900 -0.30531100<br>H 3.28216100 2.61959500 0.13277100<br>H 2.44991600 2.36580100 -1.38715500<br>H -0.28100200 0.81688200 1.40539600<br>C 5.26774900 -3.26733100 0.07959700<br>H 4.98789800 -3.62423800 1.06979900<br>H 6.34530300 -3.33507000 -0.06514100<br>H 4.74023400 -3.85682600 -0.66915200<br>O 4.97918100 -1.86470000 -0.06697700<br>H 0.41605600 -2.00210500 0.54843100<br>C -1.29742800 -0.16937700 -0.16843300<br>C -1.39276200 -0.57856900 -1.50054600		

C	-2.30701400	-0.56412300	0.72425700	
C	-2.46483900	-1.35235000	-1.94092900	
H	-0.62198500	-0.29627300	-2.20778200	
C	-3.37823300	-1.33898900	0.28991300	
C	-3.46266300	-1.73889200	-1.05551700	
H	-2.54174900	-1.66945300	-2.97388700	
O	-4.50679800	-2.49594100	-1.48429300	
H	-5.07863100	-2.65398800	-0.72120300	
H	-2.24207100	-0.26174300	1.76185400	
C	-4.43448600	-1.44198300	2.44629400	
H	-3.55709300	-1.84356800	2.96388000	
H	-5.33524200	-1.89420500	2.85743800	
H	-4.47716400	-0.35643400	2.58351800	
O	-4.41840300	-1.78498700	1.06594200	
Frequency and Energy at B3LYP/6-311G(d,p)in gas phase				
Zero-point correction= 0.400058 (Hartree/Particle)<br>Thermal correction to Energy= 0.425266<br>Thermal correction to Enthalpy= 0.426210<br>Thermal correction to Gibbs Free Energy= 0.343557<br>Sum of electronic and zero-point Energies= -1227.170551<br>Sum of electronic and thermal Energies= -1227.145344<br>Sum of electronic and thermal Enthalpies= -1227.144400<br>Sum of electronic and thermal Free Energies= -1227.227053				
Energy at ROB3LYP/6-311++G(2df,2p): in GAS PHASE				HF=-1228.8761036
Energy at ROB3LYP/6-311++G(2df,2p): in H2O				HF=-1227.6898043
Energy at ROB3LYP/6-311++G(2df,2p): in ETHANOL				HF=-1227.6888314
**Name of radical**			**Cyclolariciresinol-O4’-H**	
Cartesian Coordinates optimized at B3LYP/6-311G(d,p)				
0 2				
C	-1.02052200	-1.56251900	-0.59117200	
C	-2.09490300	-2.42613600	-0.48838300	
C	-3.34363200	-1.92755000	-0.07151500	
C	-3.48272900	-0.58014300	0.21523900	
C	0.07320700	0.71868100	-0.46053400	
C	-2.39072500	0.29808600	0.10540700	
C	-1.14703700	-0.19579200	-0.29064800	
C	-1.80344000	4.12097100	0.19283400	
H	-2.88888200	4.27280300	0.26284200	
H	-1.37475600	4.31273200	1.18806400	
C	1.01256200	3.08133700	-0.23626800	
H	0.72352900	4.12022700	-0.06664800	
H	1.23133400	2.97756600	-1.30907100	
C	-1.53530500	2.67245900	-0.22509100	
C	-0.13218900	2.13869300	0.15035800	
H	-1.65286600	2.64032700	-1.31599900	
H	-0.09946700	2.05817300	1.24495300	
O	-1.25354900	5.00756000	-0.78438800	
H	-1.41873400	5.91065600	-0.49738500	
O	2.15085800	2.74480400	0.55959400	
H	2.91771500	3.19294100	0.19219500	
O	-1.94822700	-3.74676100	-0.78694500	
H	-2.80618700	-4.16794300	-0.64517200	

H	-4.44302900	-0.18367900	0.52252700
C	-2.59841900	1.76410100	0.40720500
H	-3.59072300	2.06726000	0.05642000
H	-2.59921000	1.92440800	1.49569900
H	0.20752600	0.87108700	-1.53975300
C	-5.62782600	-2.48380700	0.41499000
H	-5.60742100	-2.07013800	1.42904400
H	-6.23806600	-3.38541000	0.40426600
H	-6.05742500	-1.74602200	-0.27130300
O	-4.32993300	-2.88147400	-0.00391600
H	-0.06939700	-1.97486500	-0.90486300
C	1.33378600	0.03692000	0.04503000
C	1.45056300	-0.34225000	1.41327800
C	2.37529800	-0.25836000	-0.82422000
C	2.57309600	-0.96160300	1.87434400
H	0.62989100	-0.13268300	2.08991500
C	3.54192000	-0.89272000	-0.39178600
C	3.69879400	-1.27373300	1.01680000
H	2.68355700	-1.25179900	2.91267200
O	4.72972000	-1.83128300	1.45736800
H	2.31117100	0.00714300	-1.87365800
C	5.71077400	-1.78078600	-1.09198700
H	6.31623700	-1.23695300	-0.36832500
H	6.20140700	-1.80760100	-2.06451400
H	5.54061200	-2.79008200	-0.71907200
O	4.46657400	-1.10325000	-1.34247700

Frequency and Energy at B3LYP/6-311G(d,p)in gas phase

Zero-point correction=	0.399548 (Hartree/Particle)
Thermal correction to Energy=	0.425039
Thermal correction to Enthalpy=	0.425983
Thermal correction to Gibbs Free Energy=	0.342578
Sum of electronic and zero-point Energies=	-1227.168112
Sum of electronic and thermal Energies=	-1227.142621
Sum of electronic and thermal Enthalpies=	-1227.141677
Sum of electronic and thermal Free Energies=	-1227.225082

Energy at ROB3LYP/6-311++G(2df,2p): in GAS PHASE	HF=-1227.6673043
Energy at ROB3LYP/6-311++G(2df,2p): in H2O	HF=-1227.6880382
Energy at ROB3LYP/6-311++G(2df,2p): in ETHANOL	HF=-1227.6870258
**Name of radical**	**Cyclolariciresinol-C7’-H**

Cartesian Coordinates optimized at B3LYP/6-311G(d,p)

0 2

C	0.99021500	-1.57998400	-0.20018800
C	2.11452100	-2.36670600	-0.10150600
C	3.39233500	-1.76689800	-0.05008400
C	3.50519900	-0.38162200	-0.11338300
C	-0.08505300	0.66622200	-0.33616700
C	2.37510600	0.42623900	-0.21546100
C	1.07422800	-0.15608800	-0.24021200
C	1.57447200	4.19064800	0.12105600
H	2.65476400	4.39042100	0.10948100
H	1.16111900	4.53422500	-0.83818400
C	-1.20647700	2.84596700	0.40367900

H	-0.94555300	3.86535700	0.67718800
H	-1.41861500	2.30358900	1.33609100
C	1.34858900	2.68121900	0.27931400
C	0.00302900	2.18772600	-0.31309900
H	1.35589400	2.47598000	1.35716100
H	-0.04893500	2.56008200	-1.35091200
O	0.97142300	4.88530900	1.21624000
H	1.03224700	5.82949200	1.04294600
O	-2.36777100	2.95231200	-0.40962200
H	-2.68826700	2.06244200	-0.59001100
O	2.00180900	-3.72527600	-0.05359800
H	2.89744600	-4.08311600	0.00080800
H	4.48214900	0.08631800	-0.09270200
C	2.51620800	1.91708600	-0.35736600
H	3.46156800	2.24439700	0.08733900
H	2.57023500	2.18052700	-1.42512000
C	5.75759500	-2.16452800	0.07829500
H	5.99212800	-1.61655400	-0.84052300
H	6.40471100	-3.03671400	0.15496300
H	5.92103500	-1.51355200	0.94373500
O	4.42678400	-2.66281700	0.05364400
H	0.03080700	-2.07578000	-0.25180700
C	-1.42363900	0.03362000	-0.50978100
C	-2.07914500	0.05836400	-1.74608800
C	-2.05827300	-0.61765100	0.56664500
C	-3.32760500	-0.54395900	-1.91533700
H	-1.60710600	0.54579600	-2.59073100
C	-3.29953600	-1.21759200	0.39990100
C	-3.94541200	-1.18378000	-0.85103500
H	-3.83283800	-0.52909600	-2.87354900
O	-5.16013800	-1.77284100	-1.01121100
H	-5.40567400	-2.15589100	-0.15832200
H	-1.56180300	-0.64537100	1.52754700
C	-3.45090800	-1.98706100	2.67140200
H	-2.50095100	-2.53070600	2.64592000
H	-4.17316500	-2.54596900	3.26387600
H	-3.29684100	-1.00064100	3.12096400
O	-4.01423900	-1.87576100	1.36958800

Frequency and Energy at B3LYP/6-311G(d,p)in gas phase

Zero-point correction= 0.399721 (Hartree/Particle)

Thermal correction to Energy= 0.425177

Thermal correction to Enthalpy= 0.426122

Thermal correction to Gibbs Free Energy= 0.342888

Sum of electronic and zero-point Energies= -1227.170564

Sum of electronic and thermal Energies= -1227.145108

Sum of electronic and thermal Enthalpies= -1227.144163

Sum of electronic and thermal Free Energies= -1227.227397

Energy at ROB3LYP/6-311++G(2df,2p): in GAS PHASE	HF-1227.6712237
**Name of anion**	**Cyclolariciresinol-O4-H**

Cartesian Coordinates optimized at B3LYP/6-311G(d,p			
-1 1			
C	1.30180300	-1.40607300	0.56755800
C	2.51140900	-2.17981700	0.58640200
C	3.68128300	-1.41251900	0.18639900
C	3.60317000	-0.07015700	-0.12471000
C	-0.11320100	0.68215500	0.38144100
C	2.39633100	0.65298800	-0.08771800
C	1.23173500	-0.04663900	0.25449800
C	1.22568700	4.33232100	-0.25866300
H	2.27679300	4.64368100	-0.32961600
H	0.77597600	4.44169900	-1.25700400
C	-1.40937100	2.88455200	0.13466200
H	-1.26868900	3.95611800	0.01344400
H	-1.64633600	2.71864700	1.19807200
C	1.17688700	2.86716700	0.18065700
C	-0.11763200	2.12125000	-0.22534800
H	1.26783000	2.86258800	1.27494100
H	-0.10629100	2.04071400	-1.32142400
O	0.54747500	5.16492400	0.69643900
H	0.51812900	6.05577500	0.33583100
O	-2.52857600	2.55642800	-0.69109000
H	-2.64870200	1.60177600	-0.64597300
O	2.55725400	-3.39956300	0.92452300
H	4.53097400	0.43552300	-0.38683000
C	2.38603400	2.12610000	-0.40980000
H	3.30628300	2.59226300	-0.03406500
H	2.38956700	2.30241600	-1.50150300
H	-0.30520400	0.82060100	1.45626400
C	5.06501100	-3.20407300	-0.53566900
H	4.33520300	-3.93048200	-0.17310800
H	6.08641200	-3.56241200	-0.37508200
H	4.91655200	-3.03293900	-1.61365900
O	4.94908000	-1.98400300	0.19096100
H	0.40607700	-1.95031500	0.85242300
C	-1.26803600	-0.16118600	-0.15228600
C	-1.29820500	-0.60277300	-1.47830800
C	-2.33377800	-0.52180200	0.68604500
C	-2.35987000	-1.36230600	-1.96380500
H	-0.46659100	-0.36825800	-2.13145100
C	-3.39185100	-1.29021300	0.20763300
C	-3.41377500	-1.71145300	-1.12863200
H	-2.37900200	-1.70949900	-2.99040800
O	-4.45666500	-2.46435400	-1.59825000
H	-5.04395600	-2.61293000	-0.84592400
H	-2.31242400	-0.20526700	1.72123900
C	-4.49888500	-1.42154700	2.33404500
H	-3.63004800	-1.85571900	2.84033800
H	-5.41043300	-1.87187600	2.72660200
H	-4.52138000	-0.34140100	2.51783800
O	-4.48346800	-1.70925100	0.94525100
Frequency and Energy at B3LYP/6-311G(d,p)in gas phase			

Zero-point correction=	0.398144 (Hartree/Particle)		
Thermal correction to Energy=	0.423407		
Thermal correction to Enthalpy=	0.424351		
Thermal correction to Gibbs Free Energy=	0.341938		
Sum of electronic and zero-point Energies=	-1227.240381		
Sum of electronic and thermal Energies=	-1227.215118		
Sum of electronic and thermal Enthalpies=	-1227.214173		
Sum of electronic and thermal Free Energies=	-1227.296587		
Name of anion	Cyclolariciresinol-O4'-H		
Cartesian Coordinates optimized at B3LYP/6-311G(d,p			
-1 1			
C	0.96548800	-1.59906900	0.40986600
C	2.03060200	-2.47708800	0.32723300
C	3.31399300	-1.98227800	0.03953900
C	3.50052400	-0.62320100	-0.14831400
C	-0.07154300	0.71376700	0.39074900
C	2.41886600	0.26906300	-0.05200000
C	1.13771700	-0.21782100	0.22543600
C	1.88370200	4.10534500	-0.07697400
H	2.97295800	4.23873300	-0.15571600
H	1.44814900	4.33117700	-1.06053800
C	-0.97847700	3.08777400	0.15348600
H	-0.66000500	4.12756800	0.09006000
H	-1.30040800	2.92009500	1.19356500
C	1.57611300	2.65348000	0.30357300
C	0.19606200	2.14028500	-0.17308800
H	1.61896600	2.60557400	1.39994400
H	0.23446100	2.07974400	-1.26980900
O	1.37096500	4.99174900	0.92557400
H	1.29935900	5.86842900	0.53828300
O	-2.06627700	2.96433500	-0.74861800
H	-2.33921700	2.03488900	-0.73537400
O	1.84424600	-3.81967600	0.52043100
H	2.70413100	-4.23864600	0.39085000
H	4.48652000	-0.22974100	-0.36875900
C	2.67343400	1.74440700	-0.26824600
H	3.64140800	2.01637700	0.17088200
H	2.76404700	1.94465800	-1.34724300
H	-0.19181400	0.84921900	1.47647900
C	5.61224500	-2.55257000	-0.34621800
H	5.65525200	-2.07329200	-1.33166400
H	6.21701200	-3.45945800	-0.36000400
H	6.01505200	-1.86136300	0.40411700
O	4.29619100	-2.95732400	-0.02434600
H	-0.02453800	-1.99451800	0.60193900
C	-1.37374900	0.09752700	-0.10062300
C	-1.58221300	-0.25923200	-1.44451300
C	-2.42580800	-0.18344500	0.79401500
C	-2.77017100	-0.83372600	-1.86883400
H	-0.79344000	-0.07959100	-2.17295500
C	-3.62227200	-0.75022700	0.38790600
C	-3.88689700	-1.10791500	-0.99720000

H	-2.91837900	-1.08527300	-2.91558600
O	-4.97841400	-1.60214300	-1.39783900
H	-2.32553600	0.05954700	1.84989800
C	-5.17286600	-2.20395800	1.44956700
H	-5.59079500	-2.50030800	0.48576500
H	-5.95505800	-2.14694600	2.21240500
H	-4.41927800	-2.94014900	1.77020600
O	-4.60262900	-0.90112000	1.36243600

Frequency and Energy at B3LYP/6-311G(d,p)in gas phase

Zero-point correction= 0.398078 (Hartree/Particle)
Thermal correction to Energy= 0.423290
Thermal correction to Enthalpy= 0.424235
Thermal correction to Gibbs Free Energy= 0.342225
Sum of electronic and zero-point Energies= -1227.244491
Sum of electronic and thermal Energies= -1227.219278
Sum of electronic and thermal Enthalpies= -1227.218334
Sum of electronic and thermal Free Energies= -1227.300343

Name of anion	**Cyclolariciresinol-C7’-H**

Cartesian Coordinates optimized at B3LYP/6-311G(d,p

-1 1

C	1.08930700	-1.49486900	0.10165100
C	2.28606200	-2.18450700	0.18352600
C	3.51386500	-1.51849300	0.14013400
C	3.50961900	-0.13867000	-0.06538700
C	-0.13949400	0.73678200	-0.11874000
C	2.32635400	0.57133200	-0.17819900
C	1.03486900	-0.06637100	-0.02961500
C	1.33282300	4.28275900	-0.48297100
H	2.33359300	4.72447500	-0.44447000
H	1.04892000	4.20063400	-1.54433600
C	-0.60978900	2.52578300	1.62058100
H	-0.42011700	3.56829200	1.89983700
H	-0.05882300	1.88611700	2.32759700
C	1.36105500	2.87503500	0.12711900
C	-0.05512900	2.23715600	0.19624700
H	1.75080500	2.96153900	1.15052600
H	-0.70699900	2.79951800	-0.49222500
O	0.48377200	5.22234700	0.18225700
H	-0.40868400	4.86068400	0.16183600
O	-2.01499500	2.34708100	1.75263200
H	-2.22324600	1.49010400	1.35989300
O	2.27675400	-3.55858000	0.33601400
H	3.19827100	-3.78877600	0.51704800
H	4.45887700	0.38113000	-0.17695800
C	2.33326400	1.99434400	-0.65612400
H	3.34711700	2.41223100	-0.61586400
H	2.02218700	2.00906000	-1.71473100
C	5.34855700	-2.54815400	-0.95036200
H	4.69466700	-3.09163600	-1.64451800
H	6.22641300	-3.15975900	-0.72122200
H	5.67611100	-1.62169600	-1.43640700

O	4.68920200	-2.28091300	0.28449500
H	0.19316000	-2.09390600	0.12463400
C	-1.48676900	0.20575200	-0.34519000
C	-2.41173900	0.88101400	-1.18855500
C	-1.98690900	-0.99292700	0.24698800
C	-3.68664200	0.39593300	-1.45179400
H	-2.10421300	1.79907300	-1.67416700
C	-3.25636300	-1.48264800	-0.03759200
C	-4.12580900	-0.79946500	-0.89321200
H	-4.35624100	0.93016200	-2.11818200
O	-5.38221300	-1.30024800	-1.17279200
H	-5.44988400	-2.12155900	-0.67011400
H	-1.38320700	-1.50085200	0.98194100
C	-2.96950300	-3.45809600	1.30576400
H	-2.06625600	-3.76947800	0.77000500
H	-3.56182800	-4.33745600	1.56241000
H	-2.67475900	-2.94222100	2.22692600
O	-3.80022700	-2.64541100	0.49550800

Frequency and Energy at B3LYP/6-311G(d,p)in gas phase

Zero-point correction= 0.397596 (Hartree/Particle)
Thermal correction to Energy= 0.423036
Thermal correction to Enthalpy= 0.423980
Thermal correction to Gibbs Free Energy= 0.341856
Sum of electronic and zero-point Energies= -1227.211626
Sum of electronic and thermal Energies= -1227.186186
Sum of electronic and thermal Enthalpies= -1227.185242
Sum of electronic and thermal Free Energies= -1227.267366

**Name of cationic radical**	**Cyclolariciresinol**

Cartesian Coordinates optimized at B3LYP/6-311G(d,p

1 2

C	-1.65118600	-1.10126500	-1.34625200
C	-2.93799400	-1.59540900	-1.19428700
C	-3.76846800	-1.09324000	-0.14549000
C	-3.28459600	-0.11334300	0.71873300
C	0.23704700	0.42216200	-0.64195100
C	-1.98810200	0.37191500	0.56358700
C	-1.16368100	-0.12189400	-0.48250000
C	-0.20083800	3.62425100	1.55750100
H	-1.04019200	4.00225400	2.15647600
H	0.53927400	3.19005500	2.24658700
C	0.54363500	2.75290400	-1.57497100
H	0.71015300	3.80162900	-1.32419200
H	-0.38324400	2.66941600	-2.16146200
C	-0.71671100	2.53089600	0.61768700
C	0.40505600	1.94768000	-0.28097700
H	-1.47845800	2.98890500	-0.02468300
H	1.35383500	2.05127300	0.25365700
O	0.37587200	4.66661000	0.78221100
H	0.66676400	5.36830000	1.37242700
O	1.65054100	2.21206700	-2.30751100
H	1.75701100	2.72170700	-3.11633300
O	-3.40592800	-2.52918800	-2.03415200

H	-4.32043400	-2.73956000	-1.79322500
H	-3.91179800	0.27741400	1.50942400
C	-1.41955100	1.43807300	1.45021200
H	-2.20464700	1.87653500	2.07077500
H	-0.68560300	0.99747600	2.14159000
H	0.48855700	0.35017300	-1.70348600
C	-5.94969600	-1.23447200	0.86760000
H	-5.57615900	-1.44150900	1.87312700
H	-6.84168400	-1.82464100	0.67556400
H	-6.17500000	-0.17153100	0.75421000
O	-4.99161300	-1.65149400	-0.11914700
H	-1.04507100	-1.48920400	-2.15618800
C	1.26939800	-0.45514400	0.05042700
C	0.98375900	-1.21620300	1.20965900
C	2.55831100	-0.50795800	-0.50152800
C	1.94965800	-2.00471000	1.80796200
H	-0.01035100	-1.19358900	1.63591500
C	3.53796600	-1.28592300	0.09314200
C	3.23397700	-2.05488700	1.26338300
H	1.74280800	-2.58586800	2.69749200
O	4.17643200	-2.80330700	1.83551500
H	5.00259000	-2.70825800	1.33521200
H	2.77566200	0.09459100	-1.37433200
C	5.27199400	-0.70903700	-1.47099600
H	4.71471500	-1.03455200	-2.35227300
H	6.32196100	-0.96790500	-1.57727600
H	5.16332500	0.36846100	-1.32889600
O	4.81671000	-1.41568000	-0.30388200

Frequency and Energy at B3LYP/6-311G(d,p)in gas phase

Zero-point correction=	0.413665 (Hartree/Particle)
Thermal correction to Energy=	0.439040
Thermal correction to Enthalpy=	0.439985
Thermal correction to Gibbs Free Energy=	0.357441
Sum of electronic and zero-point Energies=	-1227.553650
Sum of electronic and thermal Energies=	-1227.528275
Sum of electronic and thermal Enthalpies=	-1227.527330
Sum of electronic and thermal Free Energies=	-1227.609874

**Name of compound (2)**	**Secoisolariciresinol**		
Cartesian Coordinates optimized at B3LYP/6-311G(d,p)			
0 1			
O	0.64378000	1.47410100	2.79426400
O	-0.48077400	-2.60489900	1.56573500
O	6.31204200	1.17823100	-1.05218300
O	-5.92783800	-0.66151000	-1.88071700
O	7.14381900	-1.08943200	0.03380000
O	-7.11369300	0.87686500	0.11098000
C	0.80002200	-0.04954200	0.84344100
C	-0.72909200	-0.27874800	0.74750800
C	1.54117500	-0.23070700	-0.50387300
C	-1.43074100	0.57546400	-0.33906000
C	1.15956500	1.30505700	1.47348400

C	-1.07686700	-1.76720700	0.57802600	
C	3.03464700	-0.44968300	-0.36108900	
C	-2.93802900	0.65384600	-0.20317000	
C	3.95155300	0.51867500	-0.79615700	
C	-3.78470600	-0.05383600	-1.06158500	
C	3.52795300	-1.62765100	0.20168500	
C	-3.52971200	1.43673200	0.79086900	
C	5.31971000	0.30791000	-0.66456500	
C	-5.17193400	0.00750700	-0.95431000	
C	4.89902800	-1.84316700	0.33549200	
C	-4.91451900	1.51029300	0.91585400	
C	5.80321800	-0.88206500	-0.09376900	
C	-5.74762400	0.80823300	0.04723900	
C	5.93814900	2.41488600	-1.64226800	
C	-6.79273800	-1.68929300	-1.38151200	
H	1.17043800	-0.81510700	1.53061600	
H	-1.14800000	0.01657500	1.72055000	
H	1.36086600	0.63827300	-1.14642300	
H	1.11476200	-1.09000700	-1.03070800	
H	-1.02455500	1.59173000	-0.30848200	
H	-1.17947400	0.18052700	-1.32838100	
H	2.24421700	1.37123900	1.57302600	
H	0.84312000	2.13486100	0.82407900	
H	-2.16672600	-1.88817800	0.57326300	
H	-0.70649100	-2.14258200	-0.37991000	
H	3.58351600	1.43477200	-1.24161600	
H	-3.37962200	-0.66872900	-1.85785100	
H	2.83497900	-2.39039100	0.54064900	
H	-2.90958500	2.01344000	1.46973600	
H	-0.29385100	1.67230700	2.72869800	
H	-0.81605500	-2.33475300	2.42689600	
H	5.28505800	-2.75745000	0.77016300	
H	-5.35788200	2.13727400	1.68486400	
H	7.58666500	-0.30807600	-0.32267200	
H	-7.36359100	1.47472600	0.82288300	
H	5.33853500	3.01903500	-0.95275400	
H	5.37886800	2.25913000	-2.57119100	
H	6.86784400	2.93649300	-1.86404900	
H	-6.21833700	-2.44344600	-0.83171200	
H	-7.24444500	-2.15052300	-2.25912500	
H	-7.57328600	-1.27822800	-0.73901900	
Frequency and Energy at B3LYP/6-311G(d,p)in gas phase				
Zero-point correction= 0.433301 (Hartree/Particle)<br>Thermal correction to Energy= 0.460653<br>Thermal correction to Enthalpy= 0.461597<br>Thermal correction to Gibbs Free Energy= 0.371520<br>Sum of electronic and zero-point Energies= -1228.974265<br>Sum of electronic and thermal Energies= -1228.946913<br>Sum of electronic and thermal Enthalpies= -1228.945968<br>Sum of electronic and thermal Free Energies= -1229.036046				
Energy at ROB3LYP/6-311++G(2df,2p): in gas phase				HF=-1229.5113613
Energy at ROB3LYP/6-311++G(2df,2p): in H2O				HF=-1229.53368

Energy at ROB3LYP/6-311++G(2df,2p): in ETHANOL				HF=-1229.5325299
**Name of radical**			**Secoisolariciresinol-4O'-H**	
Cartesian Coordinates optimized at B3LYP/6-311G(d,p)				
0 2				
O	0.66185900	0.55944000	3.16217100	
O	-0.42270400	-2.97481000	0.76972400	
O	6.26951700	1.46303700	-0.74743300	
O	-5.82592400	-0.13528000	-2.03360800	
O	7.11240400	-1.01851500	-0.37201600	
O	-7.07721300	0.51523600	0.50434700	
C	0.78927000	-0.29407700	0.84010800	
C	-0.73724300	-0.51914300	0.70780500	
C	1.50439900	-0.04940000	-0.51119600	
C	-1.47047500	0.60719600	-0.06798900	
C	1.14465300	0.81422400	1.84238900	
C	-1.06666800	-1.89478700	0.10132100	
C	2.99996000	-0.29730600	-0.46918800	
C	-2.97407600	0.57673000	0.06105800	
C	3.91151700	0.75635800	-0.63206700	
C	-3.78398800	0.24099700	-1.01656400	
C	3.49917000	-1.58633400	-0.27729200	
C	-3.58817900	0.90181500	1.30527700	
C	5.28119300	0.51817800	-0.59944000	
C	-5.17795500	0.20552000	-0.91325600	
C	4.87163300	-1.82937800	-0.24346300	
C	-4.94503200	0.88094000	1.44857300	
C	5.77095700	-0.78470900	-0.40280100	
C	-5.83435400	0.53112700	0.35926400	
C	5.89053500	2.81608600	-0.95462800	
C	-7.26100100	-0.22979300	-2.12112700	
H	1.18462700	-1.22243800	1.26050300	
H	-1.13602600	-0.53635600	1.73192100	
H	1.31593500	0.97492100	-0.85220500	
H	1.06696800	-0.70596000	-1.26996600	
H	-1.11308800	1.57656100	0.29788000	
H	-1.19688000	0.55810700	-1.12574400	
H	2.23097700	0.87103100	1.93080300	
H	0.79855400	1.79479600	1.48392000	
H	-2.15396900	-2.03813100	0.08836300	
H	-0.72282500	-1.95028900	-0.93510800	
H	3.53839300	1.76109700	-0.78849800	
H	-3.34980500	-0.00477900	-1.97926800	
H	2.81035500	-2.41524000	-0.15376000	
H	-2.96093800	1.18020000	2.14636800	
H	-0.25780300	0.83170200	3.20888000	
H	-0.74612000	-3.00249300	1.67622800	
H	5.26238800	-2.82917900	-0.09685400	
H	-5.42888900	1.13193900	2.38513600	
H	7.55211500	-0.16805600	-0.50295500	
H	5.31407000	3.20164300	-0.10672800	
H	5.30631200	2.92861200	-1.87435500	
H	6.81832700	3.37860400	-1.04548600	

H	-7.64629700	-0.97797700	-1.42995500
H	-7.44590800	-0.52498100	-3.15294400
H	-7.72987900	0.72833800	-1.90235400
Frequency and Energy at B3LYP/6-311G(d,p)in gas phase			
Zero-point correction= 0.421009 (Hartree/Particle)<br>Thermal correction to Energy= 0.447943<br>Thermal correction to Enthalpy= 0.448887<br>Thermal correction to Gibbs Free Energy= 0.359196<br>Sum of electronic and zero-point Energies= -1228.355866<br>Sum of electronic and thermal Energies= -1228.328932<br>Sum of electronic and thermal Enthalpies= -1228.327988<br>Sum of electronic and thermal Free Energies= -1228.417680			
Energy at ROB3LYP/6-311++G(2df,2p): in GAS PHASE			HF=-1228.8761036
Energy at ROB3LYP/6-311++G(2df,2p): in H2O			HF=-1228.8984278
Energy at ROB3LYP/6-311++G(2df,2p): in ETHANOL			HF=-1228.8972773
**Name of radical**	**Secoisolariciresinol-O9-H**		
Cartesian Coordinates optimized at B3LYP/6-311G(d,p)			
0 2			
O	-0.60596400	-0.46107900	2.98580400
O	0.58433800	3.09194400	0.71158700
O	-6.21327200	-1.58988800	-0.66065000
O	5.98352300	-0.14402600	-1.93981200
O	-7.20506000	0.80517800	-0.12391700
O	7.05575900	-0.91800300	0.50607900
C	-0.80544700	0.48205700	0.72856700
C	0.72997400	0.62613000	0.58164100
C	-1.55974500	0.24756500	-0.59952500
C	1.40505100	-0.53097000	-0.19537400
C	-1.16248500	-0.61000700	1.75237700
C	1.12113300	1.98674200	-0.01329100
C	-3.06403500	0.38985000	-0.47402400
C	2.90471500	-0.62258300	0.00514300
C	-3.90847600	-0.71960300	-0.63805700
C	3.79765400	-0.31305600	-1.02439800
C	-3.64184600	1.62983900	-0.19498700
C	3.43822300	-1.02070200	1.23365900
C	-5.28727300	-0.58373300	-0.52118700
C	5.17816800	-0.39936000	-0.85983800
C	-5.02382300	1.77069400	-0.07736300
C	4.81516300	-1.11213800	1.41675700
C	-5.85523600	0.67090200	-0.23725800
C	5.69600000	-0.81245200	0.37906200
C	-5.75395800	-2.89989400	-0.96364700
C	6.88427600	0.96277200	-1.81544700
H	-1.15287800	1.42890500	1.15125100
H	1.12444800	0.60819800	1.60692800
H	-1.32154800	-0.74725600	-0.99147000
H	-1.19784300	0.96332800	-1.34455800
H	0.95425800	-1.47878900	0.11696800
H	1.19037700	-0.43132500	-1.26427700
H	-2.26011100	-0.58416600	1.93207600
H	-0.98950400	-1.63316200	1.36507800

H	2.21383400	2.05651300	-0.07514000	
H	0.73273900	2.09220800	-1.03006800	
H	-3.47496000	-1.68589100	-0.86531000	
H	3.43568800	-0.00643400	-1.99980900	
H	-3.00800100	2.50169000	-0.07402100	
H	2.77679000	-1.27232900	2.05607400	
H	0.94836300	3.06382800	1.60257600	
H	-5.47546600	2.73232300	0.13486700	
H	5.21456500	-1.43682000	2.37398800	
H	-7.59176800	-0.06755000	-0.27483800	
H	7.26211800	-1.22646000	1.39430600	
H	-5.10109500	-3.28462300	-0.17273200	
H	-5.22114200	-2.92236300	-1.92030100	
H	-6.64409500	-3.52311600	-1.03130800	
H	6.33317100	1.88784700	-1.61106100	
H	7.38643900	1.04875200	-2.77861500	
H	7.62282800	0.79135100	-1.03018800	
Frequency and Energy at B3LYP/6-311G(d,p)in gas phase				
Zero-point correction= 0.418559 (Hartree/Particle)<br>Thermal correction to Energy= 0.445510<br>Thermal correction to Enthalpy= 0.446454<br>Thermal correction to Gibbs Free Energy= 0.356862<br>Sum of electronic and zero-point Energies= -1228.321030<br>Sum of electronic and thermal Energies= -1228.294079<br>Sum of electronic and thermal Enthalpies= -1228.293135<br>Sum of electronic and thermal Free Energies= -1228.382727				
Energy at ROB3LYP/6-311++G(2df,2p): in GAS PHASE				HF=-1228.8399398
**Name of anion**		**Secoisolariciresinol-A4**		
Cartesian Coordinates optimized at B3LYP/6-311G(d,p				
-1 1				
O	-0.43158900	-1.09028300	2.77380800	
O	0.61795400	2.91019800	1.10764600	
O	-6.25804400	-1.39816300	-0.83668600	
O	6.08248100	0.49317500	-1.76380800	
O	-7.19837900	0.89967100	0.08872000	
O	7.04690200	-1.10462700	0.41579000	
C	-0.75928900	0.29005300	0.73257200	
C	0.76087300	0.50324900	0.52556500	
C	-1.56627200	0.33019900	-0.58829400	
C	1.44482900	-0.48546500	-0.46052300	
C	-1.06983800	-1.00553900	1.50086300	
C	1.09673300	1.95191300	0.15308600	
C	-3.06354600	0.47583900	-0.40506200	
C	2.93229000	-0.65110200	-0.24342900	
C	-3.93499300	-0.57619100	-0.72350600	
C	3.88545300	-0.00426800	-1.05045300	
C	-3.61290600	1.66455200	0.07959600	
C	3.43072600	-1.44060500	0.80264200	
C	-5.30827900	-0.43651600	-0.55752600	
C	5.24869500	-0.13572200	-0.84342400	
C	-4.99018600	1.80969800	0.24599400	
C	4.79185300	-1.57614100	1.03628600	

C	-5.84654200	0.76470800	-0.06971300
C	5.80515000	-0.95027700	0.22511500
C	-5.81603900	-2.66040200	-1.31144600
C	7.08751000	1.32555500	-1.19303900
H	-1.10115000	1.11999000	1.36016100
H	1.23304000	0.34245300	1.50455400
H	-1.35730600	-0.57264100	-1.17162700
H	-1.20955600	1.16938600	-1.19264200
H	0.95361500	-1.46307300	-0.36986500
H	1.25380600	-0.15368700	-1.48923000
H	-2.14084500	-1.05373500	1.71025100
H	-0.81773900	-1.88129700	0.88512200
H	2.18030000	2.04025400	0.02323400
H	0.62819600	2.23352600	-0.79436200
H	-3.52246100	-1.50296800	-1.10253000
H	3.56876200	0.61201200	-1.89010200
H	-2.95594500	2.49028600	0.33079200
H	2.72959700	-1.98226100	1.44184300
H	0.51322900	-1.19507100	2.62220900
H	1.06042100	2.71765500	1.94068900
H	-5.41738700	2.73274200	0.62033400
H	5.15637200	-2.21201600	1.83889500
H	-7.59211700	0.06043900	-0.18303900
H	-5.15930300	-3.15157600	-0.58526300
H	-5.28980600	-2.56499900	-2.26763000
H	-6.71337800	-3.26193300	-1.45222000
H	6.63573700	2.19171300	-0.68370600
H	7.69829800	1.68786300	-2.02554200
H	7.69369200	0.75593100	-0.48591600
Frequency and Energy at B3LYP/6-311G(d,p)in gas phase			
Zero-point correction= 0.419805 (Hartree/Particle)<br>Thermal correction to Energy= 0.446421<br>Thermal correction to Enthalpy= 0.447365<br>Thermal correction to Gibbs Free Energy= 0.359534<br>Sum of electronic and zero-point Energies= -1228.431059<br>Sum of electronic and thermal Energies= -1228.404443<br>Sum of electronic and thermal Enthalpies= -1228.403499<br>Sum of electronic and thermal Free Energies= -1228.491330			
**Name of cationic radical**	**Secoisolariciresinol**		
Cartesian Coordinates optimized at B3LYP/6-311G(d,p			
1 2			
O	0.11469400	2.00050600	2.06994300
O	0.04992300	-2.42475300	1.41988800
O	6.52253200	0.90949300	-0.92526900
O	-6.05072200	-1.01169900	-1.52479900
O	7.03930200	-1.21648200	0.53639300
O	-7.05145600	0.64122900	0.61172800
C	0.79850600	0.25182300	0.57470900
C	-0.66787900	-0.23686500	0.50854100
C	1.64336600	-0.07790000	-0.68959200
C	-1.50708200	0.39779300	-0.63267100

C	0.90656100	1.74126200	0.91256000
C	-0.76256700	-1.77230500	0.44888800
C	3.09236300	-0.34393200	-0.37260900
C	-2.98205300	0.44477000	-0.31727500
C	4.11099600	0.49156400	-0.83986300
C	-3.91220400	-0.29032200	-1.03012100
C	3.43080000	-1.47803000	0.40986200
C	-3.44203700	1.26916600	0.73966900
C	5.43920200	0.20596200	-0.54092500
C	-5.28774500	-0.24264300	-0.74216100
C	4.74457000	-1.77291300	0.71472200
C	-4.78780700	1.33700900	1.04575000
C	5.76548200	-0.94004900	0.24827100
C	-5.73066800	0.59491300	0.32765000
C	6.36144000	2.08108400	-1.73876200
C	-7.48923400	-1.09664400	-1.45125200
H	1.23396200	-0.28816900	1.41894100
H	-1.10800300	0.07821700	1.46055700
H	1.56136900	0.72697100	-1.42619400
H	1.23744800	-0.97395500	-1.16776500
H	-1.16589400	1.42310700	-0.80503300
H	-1.34613100	-0.14268200	-1.57028400
H	1.95991800	1.98805800	1.09637500
H	0.56685200	2.35540900	0.06526300
H	-1.81078900	-2.07945600	0.54435700
H	-0.41183200	-2.14355100	-0.51787100
H	3.86222500	1.35333200	-1.44510100
H	-3.61346700	-0.92992600	-1.85193300
H	2.63567600	-2.12075400	0.77230000
H	-2.72741600	1.85058300	1.31153400
H	0.33043800	2.87747700	2.40051200
H	-0.33990600	-2.29259200	2.29031700
H	5.01392200	-2.63925400	1.30532000
H	-5.13229600	1.97449000	1.85422800
H	7.60813500	-0.54307400	0.13174600
H	-7.21181100	1.24188800	1.35010800
H	5.77023100	2.83373000	-1.21142000
H	5.89002500	1.82337400	-2.69019200
H	7.36625600	2.45640600	-1.91359100
H	-7.80505500	-1.48691900	-0.48455200
H	-7.75783100	-1.78892700	-2.24520500
H	-7.94427000	-0.12258400	-1.62784500

Frequency and Energy at B3LYP/6-311G(d,p)in gas phase

Zero-point correction= 0.434325 (Hartree/Particle)
Thermal correction to Energy= 0.461329
Thermal correction to Enthalpy= 0.462274
Thermal correction to Gibbs Free Energy= 0.373384
Sum of electronic and zero-point Energies= -1228.727164
Sum of electronic and thermal Energies= -1228.700160
Sum of electronic and thermal Enthalpies= -1228.699216
Sum of electronic and thermal Free Energies= -1228.788105

Name of compound (3)	Isoliovil
Cartesian Coordinates optimized at B3LYP/6-311G(d,p)	

0 1

C	-3.79005900	2.22325100	0.28348500
C	-4.90047300	1.39747200	0.22762400
C	-4.75477800	0.08458700	-0.26162500
C	-3.51297700	-0.37719700	-0.67399800
C	-2.38078600	0.45414000	-0.61163900
C	-2.54251100	1.75247300	-0.13477100
C	0.77568500	-1.88336700	-0.72869500
H	1.44791000	-1.22244900	-1.28273200
C	-0.42589000	-1.13068600	-0.12535500
C	0.06530900	-0.74200500	1.28436300
H	-1.19677700	-1.89895600	0.03254000
O	1.47528700	-2.44363200	0.38297200
O	-6.12043300	1.84839700	0.63039900
H	-6.74829700	1.12566600	0.49890500
H	-3.41099000	-1.38323000	-1.06277200
C	-1.03240700	-0.07226300	-1.06085500
H	-1.13711200	-0.54452100	-2.04429700
H	-0.33667100	0.75813300	-1.19441100
C	-5.90975700	-1.95797400	-0.78116700
H	-5.58377900	-1.98439500	-1.82636900
H	-6.93379000	-2.32119800	-0.71231800
H	-5.25636800	-2.59814900	-0.17863600
O	-5.92983500	-0.62780700	-0.28188900
H	-3.91537400	3.23436500	0.65174800
H	-0.77689900	-0.66734500	1.97691200
H	-1.69031700	2.42157600	-0.09628300
C	0.89311800	-1.98995700	1.61933500
H	1.68814600	-1.79406600	2.33792000
H	0.24979600	-2.77761000	2.03139100
C	0.82365500	0.59748200	1.45138400
C	2.02776500	0.89426700	0.56985900
C	2.00823300	1.98764300	-0.29527000
C	3.20548100	0.13128800	0.65514400
C	3.11092600	2.30503900	-1.09211500
H	1.12242600	2.61084100	-0.35479900
C	4.29600300	0.43064300	-0.14452000
H	3.25176200	-0.70606000	1.33384000
C	4.25684800	1.52880500	-1.02839800
H	3.09445300	3.15259300	-1.76647200
H	0.08932700	1.39120700	1.26635000
O	1.19056300	0.61369600	2.84256900
H	1.72002000	1.40416700	2.98996400
O	5.33455100	1.81947800	-1.80285800
H	6.00826400	1.15210500	-1.61376100
C	5.57307000	-1.46999800	0.57308700
H	6.53911100	-1.90450900	0.32216200
H	4.77087500	-2.16207600	0.29993100
H	5.53439700	-1.27007500	1.64907900
O	5.48070100	-0.26030600	-0.17563700
O	0.42170500	-2.88010200	-1.65119600

H -0.06915100 -3.55567000 -1.16800100		
Frequency and Energy at B3LYP/6-311G(d,p)in gas phase		
Zero-point correction= 0.418061 (Hartree/Particle)<br>Thermal correction to Energy= 0.444110<br>Thermal correction to Enthalpy= 0.445054<br>Thermal correction to Gibbs Free Energy= 0.359969<br>Sum of electronic and zero-point Energies= -1303.030093<br>Sum of electronic and thermal Energies= -1303.004045<br>Sum of electronic and thermal Enthalpies= -1303.003101<br>Sum of electronic and thermal Free Energies= -1303.088185		
Energy at ROB3LYP/6-311++G(2df,2p): in gas phase		HF=-1228.8399398
Energy at ROB3LYP/6-311++G(2df,2p): in H2O		HF-1303.5774583
Energy at ROB3LYP/6-311++G(2df,2p): in ETHANOL		HF=-1303.5765281
**Name of radical**	**Isoliovil-O4-H**	
Cartesian Coordinates optimized at B3LYP/6-311G(d,p)		
0 2<br>C -3.78513500 2.07498400 0.34349500<br>C -4.95927400 1.23559600 0.23461200<br>C -4.73968200 -0.08554200 -0.37162200<br>C -3.46275800 -0.46398300 -0.78561000<br>C -2.36116100 0.37710400 -0.64880500<br>C -2.55111500 1.66528300 -0.07783600<br>C 0.84098300 -1.87217900 -0.86652300<br>H 1.50350100 -1.16195100 -1.36835000<br>C -0.38207100 -1.18554300 -0.22549100<br>C 0.08578400 -0.88224900 1.21232000<br>H -1.14020300 -1.97540800 -0.13065700<br>O 1.54097900 -2.48850300 0.21295600<br>O -6.08272900 1.62055200 0.62968000<br>H -3.35724500 -1.44515400 -1.23592400<br>C -0.99467300 -0.07971700 -1.10366300<br>H -1.07645400 -0.49477300 -2.11442900<br>H -0.31616400 0.77251200 -1.16972700<br>C -7.08550100 -0.77113000 -0.23808700<br>H -7.20991600 -0.60287700 0.83055500<br>H -7.58567000 -1.68835100 -0.54602700<br>H -7.48418500 0.08579900 -0.77933300<br>O -5.70219000 -0.99584000 -0.57011100<br>H -3.94812700 3.05368500 0.77913000<br>H -0.76452700 -0.87376200 1.89935800<br>H -1.70034600 2.33169100 0.01217400<br>C 0.93439400 -2.13512800 1.47008900<br>H 1.71620600 -1.97486800 2.21132200<br>H 0.30151800 -2.96166800 1.81625400<br>C 0.81637000 0.45735000 1.48189800<br>C 2.01679000 0.84354500 0.63173800<br>C 1.97579900 1.99735300 -0.15006500<br>C 3.21136600 0.10285900 0.66774200<br>C 3.07456100 2.39906200 -0.91342300<br>H 1.07697800 2.60447200 -0.16687400<br>C 4.29926100 0.48749100 -0.09846900<br>H 3.27376400 -0.78272900 1.28087100		

C	4.23863400	1.64772300	-0.89841400
H	3.04176100	3.29426300	-1.52238200
H	0.06714100	1.24898400	1.35076600
O	1.17355900	0.37832700	2.87216000
H	1.69002900	1.16343800	3.08164100
O	5.31301300	2.02152000	-1.63975700
H	6.00277200	1.35917000	-1.49588700
C	5.62533500	-1.42427800	0.49244900
H	6.60592000	-1.81066900	0.22054600
H	4.84703100	-2.11999900	0.16492100
H	5.57331300	-1.30009800	1.57916800
O	5.50070800	-0.16887400	-0.17211000
O	0.51474400	-2.80806400	-1.85813100
H	0.03078600	-3.52625300	-1.43261100

Frequency and Energy at B3LYP/6-311G(d,p)in gas phase

Zero-point correction= 0.404939 (Hartree/Particle)
Thermal correction to Energy= 0.430853
Thermal correction to Enthalpy= 0.431797
Thermal correction to Gibbs Free Energy= 0.346181
Sum of electronic and zero-point Energies= -1302.403506
Sum of electronic and thermal Energies= -1302.377591
Sum of electronic and thermal Enthalpies= -1302.376647
Sum of electronic and thermal Free Energies= -1302.462263

Energy at ROB3LYP/6-311++G(2df,2p): in GAS PHASE	HF=-1302.9139417
Energy at ROB3LYP/6-311++G(2df,2p): in H2O	HF=-1302.9337805
Energy at ROB3LYP/6-311++G(2df,2p): in ETHANOL	HF=-1302.932819
**Name of radical**	**Isoliovil-O4’-H**

Cartesian Coordinates optimized at B3LYP/6-311G(d,p)

0 2

C	-3.83115200	2.32675200	-0.31663500
C	-4.93869700	1.56362000	0.01329300
C	-4.85520300	0.15954500	-0.07236700
C	-3.67607700	-0.45063300	-0.47567900
C	-2.54683800	0.31966800	-0.80449200
C	-2.64666200	1.70610400	-0.72251300
C	0.58330500	-2.07300400	-0.67529700
H	1.20755700	-1.55539600	-1.40935400
C	-0.58288000	-1.20391300	-0.16397000
C	-0.01620100	-0.54795600	1.11141100
H	-1.33286400	-1.92211000	0.19694100
O	1.36653500	-2.37474500	0.47456500
O	-6.09691100	2.15904100	0.40781700
H	-6.73586600	1.45471900	0.58005600
H	-3.62518200	-1.52978100	-0.55411900
C	-1.26702500	-0.35959500	-1.24861700
H	-1.48045100	-1.03675000	-2.08366500
H	-0.57941500	0.39210300	-1.64089200
C	-6.06118800	-1.90037700	0.20677100
H	-5.87113700	-2.25945800	-0.81021500
H	-7.06681900	-2.18692300	0.50957100
H	-5.33399800	-2.34585500	0.89405200
O	-6.02190700	-0.48091200	0.26666400

H	-3.90914100	3.40551300	-0.25558700
H	-0.82377100	-0.32179800	1.81143900
H	-1.79630100	2.32392200	-0.99039000
C	0.83807600	-1.70945500	1.63392300
H	1.67248800	-1.39635200	2.25919900
H	0.21976300	-2.40547200	2.21572200
C	0.75329900	0.79496000	0.98948700
C	2.09119900	0.78323000	0.26602000
C	2.17172500	1.13976600	-1.11005000
C	3.26204000	0.47930600	0.95044800
C	3.36849400	1.14405500	-1.76723800
H	1.27067700	1.43074500	-1.63775300
C	4.50501300	0.46456700	0.31415300
H	3.23654500	0.24523600	2.00644700
C	4.61407500	0.80333800	-1.11118700
H	3.44868500	1.41911000	-2.81224200
H	0.09374200	1.49896000	0.46639000
O	0.91104200	1.21297400	2.35011400
H	1.38718500	2.05023100	2.35135500
O	5.70498200	0.81889500	-1.72259000
C	6.89227700	0.01594400	0.60717000
H	7.46356500	-0.29293700	1.48135100
H	7.25999900	0.96440600	0.21873400
H	6.95292800	-0.73579000	-0.17861000
O	5.54299900	0.14429200	1.09540300
O	0.17191800	-3.24386400	-1.33375600
H	-0.23293000	-3.81729700	-0.67195600
Frequency and Energy at B3LYP/6-311G(d,p)in gas phase			
Zero-point correction=	0.404636 (Hartree/Particle)		
Thermal correction to Energy=	0.430622		
Thermal correction to Enthalpy=	0.431567		
Thermal correction to Gibbs Free Energy=	0.345192		
Sum of electronic and zero-point Energies=	-1302.400194		
Sum of electronic and thermal Energies=	-1302.374208		
Sum of electronic and thermal Enthalpies=	-1302.373263		
Sum of electronic and thermal Free Energies=	-1302.459638		
Energy at ROB3LYP/6-311++G(2df,2p): in GAS PHASE			HF=-1302.9110749
Energy at ROB3LYP/6-311++G(2df,2p): in H2O			HF=-1302.9333989
Energy at ROB3LYP/6-311++G(2df,2p): in ETHANOL			HF=-1302.9322616
**Name of radical**	**Isoliovil-C7’-H**		
Cartesian Coordinates optimized at B3LYP/6-311G(d,p)			
0 2			
C	3.89655100	-2.09366800	0.35256200
C	5.02543800	-1.33676700	0.08007100
C	4.87298100	-0.05232000	-0.47382400
C	3.60614400	0.44940400	-0.74167600
C	2.46022400	-0.31512300	-0.46437800
C	2.62365400	-1.58709300	0.08168300
C	-0.53795100	2.10858800	0.02560500
H	-1.18151400	1.77325400	-0.79380200
C	0.47745200	1.03484900	0.44117800
C	-0.27815600	0.23477200	1.54805800

H	1.28873800	1.57173000	0.95279100	
O	-1.34482200	2.35279500	1.18502900	
O	6.26916300	-1.82954200	0.33919100	
H	6.90359700	-1.15173300	0.07120600	
H	3.49157000	1.43485900	-1.17715200	
C	1.08974400	0.26913500	-0.74422100	
H	1.17224100	0.97315200	-1.57852600	
H	0.39936200	-0.51658500	-1.06444200	
C	6.03143700	1.89047800	-1.28487700	
H	5.56042000	1.87236900	-2.27355200	
H	7.06942800	2.20391300	-1.38453100	
H	5.49862700	2.59891700	-0.64116300	
O	6.06533200	0.59372900	-0.70636200	
H	4.02864600	-3.08250200	0.77550200	
H	0.44728600	-0.17047700	2.25541100	
H	1.75169600	-2.18690000	0.31142000	
C	-1.04739100	1.39405200	2.21529300	
H	-1.98435000	1.08330100	2.68010500	
H	-0.42281700	1.86823100	2.98083100	
C	-1.08728500	-0.95874200	1.10847900	
C	-2.32105100	-1.05254800	0.40175800	
C	-2.73963700	-2.29790800	-0.14771300	
C	-3.21230000	0.05322000	0.23304100	
C	-3.96522700	-2.44371000	-0.78302000	
H	-2.07540000	-3.15445600	-0.13937300	
C	-4.42497400	-0.10771700	-0.40453200	
H	-2.94383300	1.02690600	0.61286100	
C	-4.82699900	-1.36334400	-0.91377700	
H	-4.26270000	-3.39632100	-1.20496300	
O	-0.41432300	-2.12268900	1.41960900	
H	-1.03514000	-2.86028700	1.40104400	
O	-6.02870200	-1.50271300	-1.53454800	
H	-6.45298300	-0.63389000	-1.52593900	
C	-5.07021400	2.19358400	-0.13965600	
H	-5.92852700	2.80291800	-0.41731600	
H	-4.16663300	2.59719900	-0.60587600	
H	-4.94965000	2.20346300	0.94814900	
O	-5.35424500	0.88010500	-0.61243600	
O	0.03211600	3.29839400	-0.44050400	
H	0.56114700	3.66748400	0.27717000	
Frequency and Energy at B3LYP/6-311G(d,p)in gas phase				
Zero-point correction= 0.405132 (Hartree/Particle)<br>Thermal correction to Energy= 0.431085<br>Thermal correction to Enthalpy= 0.432029<br>Thermal correction to Gibbs Free Energy= 0.346863<br>Sum of electronic and zero-point Energies= -1302.404628<br>Sum of electronic and thermal Energies= -1302.378676<br>Sum of electronic and thermal Enthalpies= -1302.377732<br>Sum of electronic and thermal Free Energies= -1302.462897				
Energy at ROB3LYP/6-311++G(2df,2p): in GAS PHASE				HF=-1302.9162507
**Name of radical**			**Isoliovil-O7’-H**	

Cartesian Coordinates optimized at B3LYP/6-311G(d,p)			
0 2			
C	3.82734300	-2.33961900	-0.08956700
C	4.92755100	-1.54444100	0.18444100
C	4.83735800	-0.15305300	-0.02060900
C	3.65837600	0.41327900	-0.48392800
C	2.53634900	-0.38941900	-0.75560100
C	2.64298200	-1.76340700	-0.55653500
C	-0.59731400	2.00004900	-0.82966300
H	-1.23367000	1.43217200	-1.51241700
C	0.55495600	1.15846400	-0.25070900
C	-0.02591300	0.59904100	1.06412400
H	1.30339200	1.89345300	0.08243700
O	-1.36333800	2.41872800	0.29590800
O	6.08507400	-2.09706600	0.63887300
H	6.71777900	-1.37643100	0.75874400
H	3.60257800	1.48135700	-0.65575800
C	1.25582900	0.24328800	-1.26211100
H	1.47135500	0.86126200	-2.14123000
H	0.57499400	-0.53768100	-1.60424200
C	6.02749400	1.93183900	0.10463000
H	5.85090700	2.21055300	-0.93966700
H	7.02635200	2.24907900	0.39921500
H	5.28672100	2.42294500	0.74471900
O	5.99751600	0.52107000	0.27293000
H	3.90971500	-3.40864100	0.06473300
H	0.76942400	0.41248600	1.78600500
H	1.79858500	-2.40689600	-0.77753800
C	-0.89883900	1.78409300	1.49554900
H	-1.76578400	1.49912300	2.08963900
H	-0.30142100	2.49362500	2.08333800
C	-0.81998200	-0.79644600	0.98420300
C	-2.12392400	-0.81218800	0.19827700
C	-2.16891500	-1.24636100	-1.12410900
C	-3.32306200	-0.45775100	0.83377200
C	-3.37466800	-1.28966600	-1.82776200
H	-1.26574200	-1.58000800	-1.62161300
C	-4.52122900	-0.49337600	0.13829600
H	-3.30000500	-0.19589800	1.88205700
C	-4.55372200	-0.90595700	-1.20897100
H	-3.41486900	-1.62903100	-2.85566000
H	-0.08941800	-1.48056400	0.52180800
O	-0.97206200	-1.09888900	2.30379200
O	-5.73271300	-0.94595400	-1.88398500
H	-6.42055400	-0.64918400	-1.27293600
C	-5.83880700	0.30251700	1.98170200
H	-6.88980100	0.52572300	2.15707800
H	-5.24428400	1.21201700	2.11530100
H	-5.50572800	-0.46126400	2.69204300
O	-5.75614300	-0.16968800	0.64228100
O	-0.16542700	3.10448000	-1.58015100
H	0.24503500	3.72504000	-0.96581700
Frequency and Energy at B3LYP/6-311G(d,p)in gas phase			

Zero-point correction= 0.403687 (Hartree/Particle)<br>Thermal correction to Energy= 0.428714<br>Thermal correction to Enthalpy= 0.429658<br>Thermal correction to Gibbs Free Energy= 0.347190<br>Sum of electronic and zero-point Energies= -1302.369081<br>Sum of electronic and thermal Energies= -1302.344054<br>Sum of electronic and thermal Enthalpies= -1302.343110<br>Sum of electronic and thermal Free Energies= -1302.425578	
Energy at ROB3LYP/6-311++G(2df,2p): in GAS PHASE	HF=-1302.8804155
**Name of anion**	**Isoliovil-O4-H**
Cartesian Coordinates optimized at B3LYP/6-311G(d,p	

-1 1

C	-3.64968400	2.21519600	-0.18635500
C	-4.81642100	1.39915400	0.01794700
C	-4.58133300	-0.00557000	-0.28806700
C	-3.36420600	-0.47682000	-0.74707500
C	-2.25313000	0.36423900	-0.95073700
C	-2.43893000	1.72111100	-0.65752200
C	0.98283400	-1.89593200	-1.19687000
H	1.65738900	-1.12429700	-1.57670900
C	-0.34018700	-1.32473700	-0.66008600
C	-0.07178700	-1.13584100	0.84749400
H	-1.06035100	-2.15493600	-0.71635200
O	1.61869000	-2.53926000	-0.08030200
O	-5.93119700	1.85179500	0.41130800
H	-3.30658600	-1.54127300	-0.96878200
C	-0.93593200	-0.16481900	-1.47711400
H	-1.03360500	-0.53516900	-2.50852100
H	-0.21230100	0.65444200	-1.52611700
C	-6.30494200	-0.96154700	1.04091000
H	-5.64086000	-1.30988100	1.84792800
H	-7.12210000	-1.67996300	0.92475500
H	-6.69007600	0.03096200	1.28212800
O	-5.61511000	-0.93337700	-0.20540700
H	-3.77282600	3.27567500	0.01766300
H	-1.00612700	-1.23956500	1.40272500
H	-1.61512400	2.41560100	-0.82542100
C	0.83102000	-2.34677900	1.10863200
H	1.49983500	-2.21248300	1.95954300
H	0.22110700	-3.24110700	1.29545500
C	0.49948100	0.22074600	1.31743300
C	1.76675700	0.76349500	0.67064900
C	1.74067900	1.97191700	-0.02451400
C	2.99776700	0.10140700	0.80978200
C	2.89748000	2.50375000	-0.60112900
H	0.80267800	2.50488800	-0.12985400
C	4.14172000	0.61285800	0.22137200
H	3.04186200	-0.82520800	1.36027400
C	4.10141500	1.82750800	-0.48832500
H	2.87431200	3.43849100	-1.14873000
H	-0.29699900	0.95348800	1.15062500
O	0.70475200	0.04763100	2.73741600

H	0.97558700	0.90276700	3.08595400
O	5.24004900	2.32352900	-1.05625500
H	5.93571500	1.67550500	-0.88213400
C	5.47946100	-1.30557900	0.74822400
H	6.49652600	-1.63690400	0.54080100
H	4.76185100	-1.96156500	0.24607800
H	5.30327300	-1.33850700	1.82922800
O	5.38981900	0.02652800	0.25375600
O	0.83354300	-2.79101500	-2.27044500
H	0.17007800	-3.44015800	-2.00900300

Frequency and Energy at B3LYP/6-311G(d,p)in gas phase

Zero-point correction=	0.403402 (Hartree/Particle)
Thermal correction to Energy=	0.429299
Thermal correction to Enthalpy=	0.430243
Thermal correction to Gibbs Free Energy=	0.345505
Sum of electronic and zero-point Energies=	-1302.477069
Sum of electronic and thermal Energies=	-1302.451172
Sum of electronic and thermal Enthalpies=	-1302.450228
Sum of electronic and thermal Free Energies=	-1302.534966

**Name of anion**	**Isoliovil-O4'-H**

Cartesian Coordinates optimized at B3LYP/6-311G(d,p

-1 1

C	-3.74180200	2.25001200	0.18112300
C	-4.86886700	1.46025700	0.02411200
C	-4.71867400	0.13846800	-0.43032700
C	-3.45905600	-0.36887500	-0.71513500
C	-2.30994200	0.42492700	-0.55405400
C	-2.47512800	1.73442200	-0.10668800
C	0.71597400	-2.04982900	-0.38812300
H	1.46074400	-1.46462200	-0.93287100
C	-0.43929700	-1.18486600	0.14636000
C	0.07181700	-0.72706900	1.52801700
H	-1.26045200	-1.89405800	0.33506300
O	1.30064100	-2.64831000	0.76013400
O	-6.11493600	1.95453600	0.29962800
H	-6.73860900	1.23869300	0.12212700
H	-3.34827000	-1.38408000	-1.07686600
C	-0.94647200	-0.15221800	-0.87390700
H	-0.99974300	-0.66604500	-1.84092900
H	-0.21264700	0.64717600	-0.98615000
C	-5.87227700	-1.89035100	-0.99758400
H	-5.44574900	-1.96809200	-2.00385100
H	-6.90563300	-2.23551600	-1.01607900
H	-5.29198400	-2.51741900	-0.31161800
O	-5.91379800	-0.54346500	-0.55588800
H	-3.86713800	3.26882700	0.52870500
H	-0.76799300	-0.59186800	2.21664700
H	-1.60524700	2.36843900	0.01707600
C	0.87053100	-1.97097800	1.95668000
H	1.73861500	-1.70741300	2.55945900
H	0.24323600	-2.66008500	2.53746300
C	0.86514400	0.60409000	1.59640600

C	2.04113100	0.85442300	0.68945200
C	2.05256900	1.98804300	-0.14244000
C	3.21161600	0.05946400	0.67935500
C	3.13305200	2.30374400	-0.94910000
H	1.18404600	2.64694500	-0.14418600
C	4.30656300	0.36829300	-0.10314000
H	3.29323400	-0.83167600	1.29264300
C	4.35067500	1.53428500	-0.97917600
H	3.11888400	3.19355700	-1.57257800
H	0.13524800	1.39719200	1.38638100
O	1.23107400	0.71190100	3.00377600
H	2.05059900	1.21760100	3.00076400
O	5.35536000	1.84305400	-1.67661900
C	5.95589800	-0.97056500	-1.17065500
H	6.84913400	-1.53773300	-0.89333500
H	6.20953900	-0.16173500	-1.85795800
H	5.23687000	-1.65456900	-1.64599100
O	5.42508600	-0.44284900	0.04235900
O	0.29883500	-3.05559500	-1.28655800
H	-0.19550300	-3.69173500	-0.75561500

Frequency and Energy at B3LYP/6-311G(d,p)in gas phase

Zero-point correction= 0.403161 (Hartree/Particle)
Thermal correction to Energy= 0.429225
Thermal correction to Enthalpy= 0.430169
Thermal correction to Gibbs Free Energy= 0.344651
Sum of electronic and zero-point Energies= -1302.480926
Sum of electronic and thermal Energies= -1302.454862
Sum of electronic and thermal Enthalpies= -1302.453918
Sum of electronic and thermal Free Energies= -1302.539436

Name of anion	**Isoliovil-C7’-H**

Cartesian Coordinates optimized at B3LYP/6-311G(d,p

-1 1

C	1.69328200	-2.59855300	-0.48131300
C	3.06185600	-2.60981500	-0.25138000
C	3.76979800	-1.39950400	-0.29933400
C	3.11326800	-0.20699400	-0.57632300
C	1.72771100	-0.18858300	-0.81316200
C	1.03420700	-1.39842600	-0.76328500
C	0.51199800	3.52150100	-0.42802200
H	-0.27134400	3.44573900	-1.19413300
C	1.01608900	2.13902000	0.01132200
C	0.13698600	1.77012700	1.27948200
H	2.05122200	2.30066200	0.34608100
O	0.01891500	4.14205500	0.74703400
O	3.71790700	-3.78245600	0.02625100
H	4.64182900	-3.54519900	0.17631000
H	3.66617500	0.72392500	-0.61282100
C	1.02261700	1.11273200	-1.13007900
H	1.51403500	1.58220800	-1.99395800
H	-0.00730500	0.89588200	-1.41829700
C	5.91460600	-0.36258600	0.00527200

H	5.92040200	0.16320100	-0.95662900
H	6.92782500	-0.68703100	0.24307900
H	5.55922900	0.32143100	0.78432000
O	5.12584500	-1.53732700	-0.05064100
H	1.15045200	-3.53554100	-0.43341900
H	0.83920500	1.57984500	2.09376400
H	-0.03804400	-1.40141600	-0.92307700
C	-0.57860000	3.12186000	1.57535600
H	-1.64710300	3.04999900	1.35908600
H	-0.45477200	3.43973900	2.61314600
C	-0.74275800	0.55960200	1.27141000
C	-1.90728500	0.32063300	0.53851700
C	-2.49766000	1.22547200	-0.42094400
C	-2.61571100	-0.93437500	0.68594900
C	-3.66191200	0.91320600	-1.11463500
H	-2.04452700	2.18959800	-0.61243500
C	-3.77160000	-1.20572500	-0.02324700
H	-2.23679600	-1.63269700	1.41735400
C	-4.32355100	-0.29551600	-0.93765600
H	-4.07654600	1.62384800	-1.82470300
O	-0.29035800	-0.47661600	2.14607200
H	0.22607200	-1.10386300	1.61807000
O	-5.48791000	-0.60286000	-1.64807600
H	-5.74182700	-1.47984200	-1.33884900
C	-4.04473700	-3.36244900	1.00241600
H	-4.74166700	-4.20070600	0.93768600
H	-4.03142700	-2.97878900	2.03000500
H	-3.03506400	-3.70892000	0.74952300
O	-4.50442700	-2.39033800	0.08946000
O	1.48950800	4.35922600	-1.00335500
H	2.12252700	4.53468000	-0.29613700

Frequency and Energy at B3LYP/6-311G(d,p)in gas phase

Zero-point correction=	0.400941 (Hartree/Particle)
Thermal correction to Energy=	0.427571
Thermal correction to Enthalpy=	0.428515
Thermal correction to Gibbs Free Energy=	0.342965
Sum of electronic and zero-point Energies=	-1302.427053
Sum of electronic and thermal Energies=	-1302.400423
Sum of electronic and thermal Enthalpies=	-1302.399478
Sum of electronic and thermal Free Energies=	-1302.485029

**Name of cationic radical**	**Isoliovil**

Cartesian Coordinates optimized at B3LYP/6-311G(d,p

1 2

C	3.84156400	-2.39205000	-0.01635500
C	4.89386800	-1.49335900	0.17701200
C	4.68654900	-0.09837400	-0.07127300
C	3.44423400	0.35843400	-0.48880200
C	2.38832200	-0.54254800	-0.68165900
C	2.61489500	-1.91892200	-0.44096400
C	-0.73129900	1.79781200	-1.18721400
H	-1.34244000	1.08767100	-1.75813000
C	0.45410000	1.14230500	-0.45099200

C	-0.03004600	1.02486600	1.01207200
H	1.21050600	1.93318500	-0.44590800
O	-1.57329900	2.34622000	-0.18287800
O	6.08341800	-1.93387000	0.58345900
H	6.69334600	-1.18244600	0.65654200
H	3.29889100	1.41145200	-0.68713200
C	1.04407700	-0.07102000	-1.18489100
H	1.15634600	0.21736300	-2.23856900
H	0.34128100	-0.90670300	-1.18232600
C	5.75674000	2.06077000	-0.08929800
H	5.51620500	2.27290100	-1.13351700
H	6.75737800	2.41628600	0.14079800
H	5.03080500	2.53495200	0.57524500
O	5.79038200	0.64139900	0.13737400
H	4.01831500	-3.44374800	0.16904300
H	0.80921100	1.11533700	1.70569700
H	1.80631100	-2.62271900	-0.60112700
C	-0.90040300	2.28594200	1.08436700
H	-1.64625100	2.26157200	1.87665200
H	-0.27585600	3.17538900	1.22775300
C	-0.75214300	-0.27549200	1.45625500
C	-1.97746400	-0.73505600	0.67623600
C	-1.92760400	-1.92635800	-0.08197600
C	-3.18726500	-0.03974100	0.76299600
C	-3.03064500	-2.39734500	-0.77356200
H	-1.00886200	-2.50067500	-0.10751400
C	-4.29984200	-0.48770500	0.06586600
H	-3.25094800	0.84951800	1.36967000
C	-4.22853000	-1.68401100	-0.71562100
H	-2.99869700	-3.31272700	-1.35035200
H	-0.01220300	-1.08443200	1.40069900
O	-1.09126500	-0.02596600	2.82260300
H	-1.45131500	-0.83352800	3.20516900
O	-5.30205500	-2.11808700	-1.37806700
H	-6.03885700	-1.50695500	-1.21955700
C	-5.71580100	1.34771400	0.71145300
H	-6.73380400	1.64581000	0.47583000
H	-5.00776700	2.09552200	0.34727100
H	-5.60841000	1.21265400	1.79021400
O	-5.51182600	0.09767200	0.02879500
O	-0.19634200	2.77475900	-2.04275100
H	-0.91510700	3.17212500	-2.54662700

Frequency and Energy at B3LYP/6-311G(d,p)in gas phase

Zero-point correction=	0.418147 (Hartree/Particle)
Thermal correction to Energy=	0.443223
Thermal correction to Enthalpy=	0.444167
Thermal correction to Gibbs Free Energy=	0.362007
Sum of electronic and zero-point Energies=	-1302.774806
Sum of electronic and thermal Energies=	-1302.749729
Sum of electronic and thermal Enthalpies=	-1302.748785
Sum of electronic and thermal Free Energies=	-1302.830946

**Name of compound (4)**				**Matairesinol**
Cartesian Coordinates optimized at B3LYP/6-311G(d,p)				
0 1				
O	4.78049800	-0.12089900	0.27693900	
O	4.48918700	0.29263100	-1.91009900	
O	-2.87849500	-1.28852600	1.28682900	
O	-2.72630900	1.22519300	-1.53505700	
O	-3.15848200	-2.52849800	-1.02388600	
O	-3.38867700	2.46789400	0.69487900	
C	2.51342300	-0.27959900	1.01649100	
C	2.55490600	0.30926500	-0.40479700	
C	3.97484500	-0.10794100	1.47401500	
C	2.08191600	-1.76669200	1.04769600	
C	2.12281200	1.79489900	-0.52648000	
C	4.01872800	0.16584300	-0.81610700	
C	0.68808300	-2.01803500	0.51599800	
C	0.65974600	2.01984400	-0.21666200	
C	-0.43242100	-1.54319100	1.21944400	
C	0.48340800	-2.68897500	-0.68711100	
C	-0.32703500	1.52334400	-1.08534100	
C	0.25779000	2.68873400	0.93652700	
C	-1.71090500	-1.71937900	0.71166600	
C	-1.67199600	1.67894900	-0.78615800	
C	-0.80328400	-2.87442800	-1.20198500	
C	-1.09707900	2.85350500	1.24158500	
C	-1.90207200	-2.38586300	-0.51502500	
C	-2.06519300	2.34513000	0.39200800	
C	-2.81619700	-0.61404100	2.54070700	
C	-2.45483000	0.58156800	-2.77837300	
H	1.84407200	0.29567800	1.65989400	
H	1.95279800	-0.27785300	-1.10061900	
H	4.14154500	0.84371900	1.98711500	
H	4.32734600	-0.91823200	2.11353100	
H	2.15517100	-2.11854300	2.08354700	
H	2.80040300	-2.35846100	0.47060900	
H	2.35334100	2.11813700	-1.54627000	
H	2.73362000	2.41167500	0.14065000	
H	-0.29220000	-1.02098200	2.15749700	
H	1.33384000	-3.07556300	-1.23817900	
H	-0.03003000	0.99985300	-1.98544000	
H	1.00468100	3.09612400	1.60946700	
H	-0.96506000	-3.39844700	-2.13654500	
H	-1.41382100	3.37931400	2.13461200	
H	-3.76525300	-2.12240000	-0.38977100	
H	-3.88066400	2.06065000	-0.03134400	
H	-2.22190500	0.30026800	2.46432900	
H	-2.40214700	-1.26822300	3.31526600	
H	-3.84279900	-0.35462800	2.79145600	
H	-1.94500100	1.26373500	-3.46650900	
H	-3.42372100	0.30343100	-3.18808300	
H	-1.85387100	-0.31924100	-2.63066100	
Frequency and Energy at B3LYP/6-311G(d,p)in gas phase				

Zero-point correction= 0.390685 (Hartree/Particle)<br>Thermal correction to Energy= 0.414802<br>Thermal correction to Enthalpy= 0.415746<br>Thermal correction to Gibbs Free Energy= 0.336441<br>Sum of electronic and zero-point Energies= -1226.642708<br>Sum of electronic and thermal Energies= -1226.618592<br>Sum of electronic and thermal Enthalpies= -1226.617647<br>Sum of electronic and thermal Free Energies= -1226.696953	
Energy at ROB3LYP/6-311++G(2df,2p): in gas phase	HF=-1227.1344876
Energy at ROB3LYP/6-311++G(2df,2p): in H2O	HF=-1227.1523736
Energy at ROB3LYP/6-311++G(2df,2p): in ETHANOL	HF=-1227.1515058
**Name of radical**	**Matairesinol-O4-H**
Cartesian Coordinates optimized at B3LYP/6-311G(d,p)	

0 2

O	4.81760600	-0.19190100	0.30220500
O	4.60222600	0.27237200	-1.88388600
O	-2.91020500	-1.36222900	1.20746400
O	-2.39415200	1.18030400	-1.79094400
O	-3.16526300	-2.49406200	-1.15921100
O	-3.22177200	2.44170600	0.69134600
C	2.52683800	-0.30819700	0.97418400
C	2.62328300	0.29530900	-0.43887100
C	3.97844800	-0.17806100	1.47578900
C	2.05946800	-1.78510700	0.97199300
C	2.22601000	1.79102600	-0.56467300
C	4.09777900	0.13156700	-0.80755300
C	0.67015600	-2.00064800	0.41305800
C	0.76498300	2.03124700	-0.27561000
C	-0.45830800	-1.57715900	1.13654000
C	0.47734900	-2.59805400	-0.83082600
C	-0.20976600	1.56441100	-1.14911400
C	0.36333900	2.69156900	0.91871300
C	-1.73264500	-1.74207400	0.61271400
C	-1.57162700	1.68752200	-0.86859400
C	-0.80384600	-2.76991600	-1.36122200
C	-0.95962000	2.84055200	1.22766600
C	-1.91159700	-2.34089700	-0.64986700
C	-2.01467300	2.33584900	0.37444500
C	-2.86731900	-0.78266400	2.50856700
C	-3.82187100	1.08878900	-1.61931100
H	1.85294700	0.27142500	1.60890600
H	2.03136900	-0.27252400	-1.15855900
H	4.15353700	0.76094400	2.00908200
H	4.29285000	-1.00654800	2.11156100
H	2.10649900	-2.15460300	2.00296000
H	2.77618300	-2.38296300	0.39897700
H	2.46896900	2.10699700	-1.58359900
H	2.83938200	2.39671100	0.10958900
H	-0.32931300	-1.11648800	2.10819600
H	1.33407900	-2.94489700	-1.39848300
H	0.06330700	1.05878700	-2.06840500
H	1.12533400	3.08111700	1.58602900

H	-0.95570300	-3.23678700	-2.32677600	
H	-1.28609900	3.34309200	2.13077900	
H	-3.78478400	-2.18784200	-0.48324600	
H	-2.30316800	0.15388100	2.50345900	
H	-2.43180500	-1.47974600	3.23193500	
H	-3.90091300	-0.57381900	2.77685100	
H	-4.06381000	0.52108000	-0.72258200	
H	-4.16287500	0.56437200	-2.51013500	
H	-4.27180100	2.07790000	-1.55050100	
Frequency and Energy at B3LYP/6-311G(d,p)in gas phase				
Zero-point correction=	0.377433 (Hartree/Particle)			
Thermal correction to Energy=	0.401549			
Thermal correction to Enthalpy=	0.402494			
Thermal correction to Gibbs Free Energy=	0.321517			
Sum of electronic and zero-point Energies=	-1226.014496			
Sum of electronic and thermal Energies=	-1225.990380			
Sum of electronic and thermal Enthalpies=	-1225.989436			
Sum of electronic and thermal Free Energies=	-1226.070413			
Energy at ROB3LYP/6-311++G(2df,2p): in GAS PHASE				HF=-1226.4878817
Energy at ROB3LYP/6-311++G(2df,2p): in H2O				HF=-1226.5079905
Energy at ROB3LYP/6-311++G(2df,2p): in ETHANOL				HF=-1226.506961
**Name of radical**			**Matairesinol-O4'-H**	
Cartesian Coordinates optimized at B3LYP/6-311G(d,p)				
0 2				
O	4.72738200	-0.05992000	-0.26157800	
O	4.40888800	-0.33713100	1.94409200	
O	-2.80446900	1.42874300	-1.26005300	
O	-2.85607400	-1.22564300	1.51998600	
O	-2.93112400	2.75026400	1.06189800	
O	-3.48574400	-2.41915000	-0.73898900	
C	2.47457200	0.16300100	-1.01793000	
C	2.48396800	-0.36852000	0.42638100	
C	3.92674800	-0.10218000	-1.46039400	
C	2.13053500	1.67160800	-1.11549300	
C	2.00747800	-1.83705800	0.59639500	
C	3.94972600	-0.25552200	0.84191900	
C	0.76688400	2.02440600	-0.57493000	
C	0.54703200	-2.03660700	0.25900400	
C	-0.38883600	1.57679000	-1.23476800	
C	0.64211500	2.77452400	0.62031400	
C	-0.45008900	-1.53285800	1.11283800	
C	0.15957500	-2.69187800	-0.90697100	
C	-1.65100400	1.82585400	-0.71545600	
C	-1.79180600	-1.67097000	0.78723700	
C	-0.58841500	3.04268300	1.16089700	
C	-1.19091500	-2.83403600	-1.24170600	
C	-1.81220000	2.56850100	0.55096900	
C	-2.16915300	-2.31921200	-0.40777700	
C	-2.78312800	0.70850300	-2.49373300	
C	-2.61480600	-0.52796000	2.73997900	
H	1.77576200	-0.39965300	-1.63987900	
H	1.89431600	0.25845600	1.09747100	

H	4.04627500	-1.08793100	-1.91896500	
H	4.31926200	0.65266600	-2.14287100	
H	2.19449500	1.96448100	-2.17006600	
H	2.89402900	2.24831000	-0.58391700	
H	2.20598100	-2.12384200	1.63341300	
H	2.61776000	-2.49562600	-0.03000000	
H	-0.28130800	1.00985600	-2.15093600	
H	1.54126800	3.13317800	1.11038100	
H	-0.16480100	-1.03022800	2.02897700	
H	0.91395800	-3.10871000	-1.56570500	
H	-0.70039900	3.61112700	2.07679700	
H	-1.49660700	-3.34769800	-2.14555300	
H	-3.98865400	-1.99379800	-0.03003500	
H	-2.23061100	-0.22853300	-2.38978700	
H	-2.34773100	1.31827500	-3.29166300	
H	-3.82396300	0.49207100	-2.72181900	
H	-2.10266600	-1.17210200	3.46225200	
H	-3.59480200	-0.25173700	3.12280000	
H	-2.03211500	0.37972600	2.56560300	
Frequency and Energy at B3LYP/6-311G(d,p)in gas phase				
Zero-point correction= 0.377437 (Hartree/Particle)<br>Thermal correction to Energy= 0.401504<br>Thermal correction to Enthalpy= 0.402448<br>Thermal correction to Gibbs Free Energy= 0.321126<br>Sum of electronic and zero-point Energies= -1226.012888<br>Sum of electronic and thermal Energies= -1225.988821<br>Sum of electronic and thermal Enthalpies= -1225.987877<br>Sum of electronic and thermal Free Energies= -1226.069199				
Energy at ROB3LYP/6-311++G(2df,2p): in GAS PHASE				HF=-1226.4884588
Energy at ROB3LYP/6-311++G(2df,2p): in H2O				HF=-1226.5129545
Energy at ROB3LYP/6-311++G(2df,2p): in ETHANOL				HF=-1226.5116742
**Name of anion**		**Matairesinol-O4-H**		
Cartesian Coordinates optimized at B3LYP/6-311G(d,p				
-1 1				
O	4.75269700	0.19109100	0.25324500	
O	4.49556100	0.39783500	-1.96532700	
O	-2.76020700	-1.59029700	1.04844000	
O	-2.65944600	1.78844100	-1.90640600	
O	-2.86357900	-3.29359300	-0.94506100	
O	-3.44312500	2.58255400	0.75034500	
C	2.48891800	-0.14058100	0.93061200	
C	2.52192400	0.42728900	-0.49700400	
C	3.90667700	0.19568100	1.42387400	
C	2.22748200	-1.66276100	0.99794600	
C	2.05014500	1.91850800	-0.60279500	
C	3.98949900	0.33122000	-0.87888100	
C	0.87602200	-2.11053800	0.48309300	
C	0.60267900	2.13746100	-0.24907500	
C	-0.30318300	-1.60810700	1.05946000	
C	0.77157200	-3.02585400	-0.56093100	
C	-0.42239600	1.85014000	-1.17115300	
C	0.20344900	2.59317700	1.01271900	

C	-1.54377800	-2.01166700	0.58969100
C	-1.76285400	2.00664800	-0.86913800
C	-0.47675500	-3.43696000	-1.04004000
C	-1.13968500	2.73589100	1.34670600
C	-1.63383200	-2.93024200	-0.47567700
C	-2.22014300	2.45731600	0.43789100
C	-2.79902200	-0.57264200	2.05756200
C	-3.75730400	0.92462900	-1.62039700
H	1.73828100	0.38244900	1.52592900
H	1.92782800	-0.16463600	-1.19440200
H	3.96092800	1.18737400	1.88309300
H	4.31141100	-0.53968400	2.12259000
H	2.33965700	-1.97273500	2.04590400
H	3.01226700	-2.18753000	0.44108300
H	2.25811000	2.23781300	-1.62975600
H	2.68628700	2.53270100	0.04692700
H	-0.24553700	-0.87065200	1.84918200
H	1.67211400	-3.41981600	-1.02042100
H	-0.17906800	1.50643800	-2.17428300
H	0.96338200	2.86061700	1.74739000
H	-0.56229800	-4.13411500	-1.86545300
H	-1.43036300	3.11722500	2.32245300
H	-3.50580800	-2.77197800	-0.44270100
H	-2.21574300	0.29968100	1.76051300
H	-2.43512700	-0.97519300	3.01046400
H	-3.84138100	-0.27727200	2.14680600
H	-3.40923700	-0.09232300	-1.38772800
H	-4.35890200	0.88795800	-2.53295900
H	-4.33849500	1.31379600	-0.78330100

Frequency and Energy at B3LYP/6-311G(d,p)in gas phase

Zero-point correction= 0.375951 (Hartree/Particle)
Thermal correction to Energy= 0.399886
Thermal correction to Enthalpy= 0.400831
Thermal correction to Gibbs Free Energy= 0.321627
Sum of electronic and zero-point Energies= -1226.097680
Sum of electronic and thermal Energies= -1226.073744
Sum of electronic and thermal Enthalpies= -1226.072800
Sum of electronic and thermal Free Energies= -1226.152004

**Name of anion**	**Matairesinol-O4’-H**

Cartesian Coordinates optimized at B3LYP/6-311G(d,p

-1 1

O	4.65109400	-0.46207200	-0.22759000
O	4.33495800	-0.28250100	1.98432100
O	-2.68234000	1.69866300	-1.10387900
O	-3.15721400	-1.01883400	1.14520300
O	-2.48501600	3.16253600	1.14609600
O	-3.55881500	-2.90943000	-0.59092800
C	2.43496400	-0.10423700	-1.03440300
C	2.39288800	-0.37540500	0.47756500
C	3.83218800	-0.62016100	-1.41022700
C	2.27141600	1.40639800	-1.37631400

C	1.80020100	-1.74355500	0.91525300
C	3.86176700	-0.34829200	0.88287400
C	1.02149000	2.02632700	-0.81173000
C	0.36984300	-2.01877800	0.49780000
C	-0.25015200	1.65174800	-1.30180000
C	1.05568000	2.86498700	0.30405300
C	-0.70442800	-1.28025600	1.02985700
C	0.09662000	-3.04746200	-0.40112000
C	-1.41490600	2.06180800	-0.68038700
C	-2.01195300	-1.59549400	0.68147100
C	-0.10890900	3.28872200	0.93923600
C	-1.21524900	-3.35298800	-0.77787700
C	-1.41833800	2.88285600	0.52676700
C	-2.26726200	-2.63436300	-0.24119000
C	-2.78595900	0.88610500	-2.24591900
C	-3.10981400	0.13436300	2.00781000
H	1.65772600	-0.66859900	-1.55381000
H	1.86429500	0.42526300	0.99821900
H	3.83768500	-1.68030900	-1.68570900
H	4.30201200	-0.04483100	-2.21061200
H	2.31029100	1.49350500	-2.47251800
H	3.14865100	1.94524300	-0.99572100
H	1.89104100	-1.79288700	2.00560500
H	2.42942000	-2.55070900	0.52300400
H	-0.29921500	1.00374200	-2.17188200
H	2.02086400	3.17934300	0.69990800
H	-0.51081800	-0.44884200	1.69574900
H	0.91512700	-3.62814200	-0.81441600
H	-0.06162600	3.92986800	1.81490700
H	-1.43133000	-4.14723300	-1.48330000
H	-4.09774200	-2.25849100	-0.11711600
H	-2.29283700	-0.08663200	-2.11278300
H	-2.36071700	1.37072400	-3.13781400
H	-3.85288100	0.71979800	-2.40844400
H	-2.38744400	-0.01978700	2.81453800
H	-4.10940400	0.20280000	2.43774800
H	-2.89494800	1.05532500	1.45835200

Frequency and Energy at B3LYP/6-311G(d,p)in gas phase

Zero-point correction= 0.376292 (Hartree/Particle)
Thermal correction to Energy= 0.399934
Thermal correction to Enthalpy= 0.400878
Thermal correction to Gibbs Free Energy= 0.322622
Sum of electronic and zero-point Energies= -1226.093281
Sum of electronic and thermal Energies= -1226.069639
Sum of electronic and thermal Enthalpies= -1226.068695
Sum of electronic and thermal Free Energies= -1226.146950

**Name of cationic radical**	**Matairesinol**

Cartesian Coordinates optimized at B3LYP/6-311G(d,p

1 2

O	4.61118600	-0.45598000	0.14010600
O	4.24089800	0.96601700	-1.56393900
O	-2.84696000	-3.02653200	0.75706300

O	-2.16462300	3.46072200	-1.06806100
O	-3.34186600	-2.52535200	-1.77755300
O	-3.31763000	3.00964400	1.25295400
C	2.37827000	-0.92186800	0.80682900
C	2.39291900	0.32804000	-0.08856000
C	3.85570500	-0.98801000	1.24650600
C	2.00043300	-2.22301400	0.04026600
C	2.13778100	1.68778100	0.63392900
C	3.83050200	0.34403700	-0.63077400
C	0.57422400	-2.29594900	-0.43610300
C	0.68127000	2.02728800	0.80023700
C	-0.45071300	-2.62455100	0.46026000
C	0.24831900	-2.05088300	-1.78963700
C	-0.02591000	2.57682000	-0.27681800
C	0.01133800	1.81663100	2.02743200
C	-1.76565800	-2.70654300	0.02033600
C	-1.36560700	2.91150300	-0.13577100
C	-1.05663400	-2.12180400	-2.24375400
C	-1.32406400	2.13970500	2.18507400
C	-2.07920500	-2.44751900	-1.35096000
C	-2.03092900	2.68786800	1.11188200
C	-2.69188700	-3.37673400	2.14148900
C	-1.63203300	3.79308100	-2.36239600
H	1.71848100	-0.80913400	1.67017900
H	1.71286300	0.25285300	-0.93814700
H	4.05438700	-0.37534800	2.13002600
H	4.21422900	-1.99986200	1.43191400
H	2.19313900	-3.06577300	0.71160100
H	2.67928300	-2.34120100	-0.80904300
H	2.61406500	2.46384700	0.02836100
H	2.63781100	1.68974400	1.60593600
H	-0.21092800	-2.83872500	1.49385300
H	1.04221300	-1.82354600	-2.49152500
H	0.48672500	2.76107300	-1.21223400
H	0.56326500	1.41709300	2.87009400
H	-1.31110400	-1.95042400	-3.28195400
H	-1.84066600	1.99998500	3.12612400
H	-3.90982800	-2.79489800	-1.03863200
H	-3.63808700	3.40434500	0.42623100
H	-2.28325700	-2.53402100	2.70445700
H	-2.04707200	-4.25275900	2.24287500
H	-3.69158800	-3.60959300	2.49818500
H	-0.82521400	4.52283100	-2.26500500
H	-2.46041000	4.22670700	-2.91587400
H	-1.27507200	2.89335400	-2.86878900

Frequency and Energy at B3LYP/6-311G(d,p)in gas phase

Zero-point correction=	0.390454 (Hartree/Particle)
Thermal correction to Energy=	0.414869
Thermal correction to Enthalpy=	0.415814
Thermal correction to Gibbs Free Energy=	0.332223
Sum of electronic and zero-point Energies=	-1226.378444
Sum of electronic and thermal Energies=	-1226.354029
Sum of electronic and thermal Enthalpies=	-1226.353085

Sum of electronic and thermal Free Energies=	-1226.436676

Name of compound (5)	Hydroxymatairesinol
Cartesian Coordinates optimized at B3LYP/6-311G(d,p)	

0 1

O	0.57429400	3.76420200	1.15841900
O	0.87317800	0.98604100	-2.08561800
O	-0.83119000	4.95981700	-0.12111100
O	3.12494300	-3.17455700	0.08276000
O	-4.20540100	-2.26781700	1.25836700
O	5.47505100	-2.24278200	0.85088500
O	-4.04955100	-3.03591300	-1.27167300
C	0.37810400	1.60859600	0.15802300
C	-0.75192900	2.51905700	-0.35307900
C	1.50680500	1.43544800	-0.88163200
C	0.86132000	2.37011300	1.40376500
C	-2.17105600	2.14818100	0.14639600
C	-0.37946800	3.89575400	0.19119400
C	2.58227000	0.46627200	-0.42967700
C	-2.64434800	0.76266100	-0.24557700
C	2.30265100	-0.91139800	-0.40923200
C	3.84001800	0.90732300	-0.02825600
C	-3.18341200	-0.08807400	0.73213900
C	-2.58877800	0.30828700	-1.56332200
C	3.26594800	-1.81166200	0.01720100
C	4.81226600	0.00220800	0.40614800
C	-3.65572100	-1.35074800	0.39319100
C	-3.05796600	-0.95922600	-1.90704000
C	4.53418900	-1.35506100	0.43477900
C	-3.59314800	-1.79627500	-0.93841400
C	1.88577400	-3.74774900	-0.32116400
C	-4.37528300	-1.89885400	2.62015400
H	0.00404400	0.61760900	0.42147200
H	-0.75789200	2.56856000	-1.44097500
H	1.97340900	2.41708600	-1.05184400
H	0.32447600	2.06189600	2.30502800
H	1.93112500	2.28054600	1.58834300
H	-2.85653200	2.90884500	-0.24197800
H	-2.20892300	2.23913100	1.23737200
H	1.56644900	0.80684800	-2.72962000
H	1.33499300	-1.25375000	-0.75171600
H	4.07637300	1.96563500	-0.05880000
H	-3.23649600	0.25293900	1.75878400
H	-2.16348000	0.93579800	-2.33668100
H	5.79438800	0.33596100	0.71822900
H	-3.01694900	-1.31392700	-2.92999200
H	5.08504700	-3.12498400	0.78521400
H	-4.39792500	-3.43631200	-0.46399400
H	1.67595100	-3.53378600	-1.37396600
H	1.06018400	-3.38022200	0.29658200
H	1.99293800	-4.82211500	-0.18327000
H	-3.41223800	-1.68944500	3.09830700

H	-4.84152400	-2.75311900	3.10823800	
H	-5.02744000	-1.02424000	2.71544500	

Frequency and Energy at B3LYP/6-311G(d,p)in gas phase

Zero-point correction=	0.394846 (Hartree/Particle)
Thermal correction to Energy=	0.420414
Thermal correction to Enthalpy=	0.421359
Thermal correction to Gibbs Free Energy=	0.335821
Sum of electronic and zero-point Energies=	-1301.874168
Sum of electronic and thermal Energies=	-1301.848600
Sum of electronic and thermal Enthalpies=	-1301.847656
Sum of electronic and thermal Free Energies=	-1301.933193

Energy at ROB3LYP/6-311++G(2df,2p): in gas phase	HF=-1302.3786668
Energy at ROB3LYP/6-311++G(2df,2p): in H2O	HF=-1302.3999342
Energy at ROB3LYP/6-311++G(2df,2p): in ETHANOL	HF=-1302.3989475
**Name of radical**	**Hydroxymatairesinol-O4-H**

Cartesian Coordinates optimized at B3LYP/6-311G(d,p)

0 2

O	0.74563500	-3.96238600	-0.70077700
O	0.18888100	-0.54378500	1.91665400
O	-1.11688300	-4.87245800	0.16102100
O	3.37848300	2.82338400	-0.89319800
O	-4.08149300	1.42826500	1.71122600
O	5.69382500	2.02889300	0.10134900
O	-3.87355900	3.20566100	-0.57260500
C	0.37156700	-1.64028000	-0.27276000
C	-0.90741800	-2.43623900	0.03377500
C	1.09829600	-1.19183300	1.01949600
C	1.21060100	-2.64991400	-1.08072200
C	-2.11736100	-2.13869200	-0.88757200
C	-0.48924300	-3.89612400	-0.13168400
C	2.32960600	-0.34759600	0.74813500
C	-2.60973400	-0.71479200	-0.79287300
C	2.22686200	0.84691100	0.01101200
C	3.57260000	-0.71469200	1.25544000
C	-3.13496900	-0.22078000	0.39771000
C	-2.53566600	0.14653400	-1.92267000
C	3.34482000	1.64138200	-0.19881300
C	4.70286400	0.07551300	1.03726300
C	-3.59308300	1.09257500	0.51172800
C	-2.96244200	1.44336300	-1.84961400
C	4.59961200	1.25368900	0.31480300
C	-3.50771600	2.01040500	-0.63441400
C	2.16670900	3.33044700	-1.43968500
C	-4.66163400	2.71455100	2.00227900
H	0.15318900	-0.75704600	-0.87759500
H	-1.21501800	-2.28460600	1.06901900
H	1.41256300	-2.09003900	1.55958700
H	1.07022700	-2.53784900	-2.15915700
H	2.27667600	-2.60098200	-0.86046900
H	-2.91619800	-2.83338900	-0.60949700
H	-1.85670700	-2.36221100	-1.92670300
H	0.03918600	0.35389800	1.60188500

H	1.27155900	1.15291300	-0.39886800
H	3.66701600	-1.62536700	1.83613800
H	-3.20867300	-0.85050100	1.27678000
H	-2.14070500	-0.24399700	-2.85492400
H	5.67232800	-0.20785900	1.42852800
H	-2.91626500	2.11304900	-2.70041800
H	5.40792100	2.79638000	-0.41221000
H	1.42902500	3.52506300	-0.65431300
H	1.74475800	2.63894100	-2.17644400
H	2.42685100	4.26621400	-1.93074700
H	-5.50012700	2.92437100	1.33935700
H	-3.92378500	3.50887100	1.90168200
H	-4.99855700	2.62699700	3.03398500

Frequency and Energy at B3LYP/6-311G(d,p)in gas phase

Zero-point correction= 0.381163 (Hartree/Particle)
Thermal correction to Energy= 0.407006
Thermal correction to Enthalpy= 0.407950
Thermal correction to Gibbs Free Energy= 0.320159
Sum of electronic and zero-point Energies= -1301.244890
Sum of electronic and thermal Energies= -1301.219047
Sum of electronic and thermal Enthalpies= -1301.218103
Sum of electronic and thermal Free Energies= -1301.305894

Energy at ROB3LYP/6-311++G(2df,2p): in GAS PHASE	HF=-1301.7315547
Energy at ROB3LYP/6-311++G(2df,2p): in H2O	HF=-1301.755602
Energy at ROB3LYP/6-311++G(2df,2p): in ETHANOL	HF=-1301.754369
**Name of radical**	**Hydroxymatairesinol-O4’-H**

Cartesian Coordinates optimized at B3LYP/6-311G(d,p)

0 2

O	-3.41543300	-2.29922500	-1.02033000
O	0.03218500	-3.86243900	-0.57742100
O	-4.09028700	-1.92919600	1.08123600
O	3.76163700	0.77750900	-1.12312200
O	0.00954700	3.76844600	-0.39073700
O	4.64329500	-0.07992400	1.50066200
O	-2.50522500	4.56098000	-0.59116800
C	-1.08003100	-1.76836900	-0.85755100
C	-1.70324000	-1.81406400	0.54855400
C	0.24934900	-2.53050000	-1.03655800
C	-2.18102000	-2.38054100	-1.75409000
C	-1.43736600	-0.62481500	1.50365600
C	-3.19758500	-2.00546200	0.28543400
C	1.43603600	-1.87431900	-0.35143100
C	-1.72918500	0.75307700	0.94138000
C	2.11442200	-0.84290000	-0.99249400
C	1.86930300	-2.30790600	0.93039400
C	-0.67507100	1.58903100	0.53506700
C	-3.03728600	1.22926000	0.82792300
C	3.21157600	-0.21309600	-0.40599200
C	2.93883800	-1.71250600	1.53876100
C	-0.93169600	2.85726300	0.02735000
C	-3.29749300	2.49813700	0.31004100
C	3.67691000	-0.62710000	0.92603600

C -2.25476800 3.31856100 -0.09409100<br>C 5.00763500 1.41172800 -0.76727400<br>C 1.38401700 3.47974600 -0.16809800<br>H -0.91489500 -0.72744400 -1.14525400<br>H -1.38603700 -2.73274900 1.05459000<br>H 0.46216900 -2.54220200 -2.11652800<br>H -2.31950400 -1.83697800 -2.68937900<br>H -1.98206100 -3.43274900 -1.96496000<br>H -0.39625800 -0.67033200 1.83156000<br>H -2.05605600 -0.79706500 2.38886700<br>H 0.80429600 -4.39355600 -0.79795800<br>H 1.81412300 -0.51051600 -1.98074200<br>H 1.34100300 -3.12102200 1.41219300<br>H 0.34563100 1.24039400 0.63178800<br>H -3.86381600 0.61014200 1.15376100<br>H 3.28711600 -2.02182300 2.51717700<br>H -4.31104300 2.87161000 0.22618400<br>H -1.64929600 4.96563100 -0.78549200<br>H 5.80077400 0.67317100 -0.65604800<br>H 4.91260900 1.97066800 0.16162200<br>H 5.21760800 2.07591700 -1.60424400<br>H 1.58296400 3.30829600 0.89524700<br>H 1.93224200 4.35971700 -0.50151200<br>H 1.71260600 2.61032300 -0.74591900	
Frequency and Energy at B3LYP/6-311G(d,p)in gas phase	
Zero-point correction= 0.382130 (Hartree/Particle)<br>Thermal correction to Energy= 0.407371<br>Thermal correction to Enthalpy= 0.408315<br>Thermal correction to Gibbs Free Energy= 0.324522<br>Sum of electronic and zero-point Energies= -1301.246350<br>Sum of electronic and thermal Energies= -1301.221109<br>Sum of electronic and thermal Enthalpies= -1301.220165<br>Sum of electronic and thermal Free Energies= -1301.303958	
Energy at ROB3LYP/6-311++G(2df,2p): in GAS PHASE	HF=-1301.7316266
Energy at ROB3LYP/6-311++G(2df,2p): in H2O	HF=-1301.7544904
Energy at ROB3LYP/6-311++G(2df,2p): in ETHANOL	HF=-1301.7533654
**Name of radical**	**Hydroxymatairesinol-C7'-H**
Cartesian Coordinates optimized at B3LYP/6-311G(d,p)	
0 2<br>O -0.33679900 4.39111100 0.73041900<br>O -0.11114500 -0.16652600 1.96748600<br>O -1.91896000 4.61156800 -0.84216900<br>O 4.36352000 -2.11433300 0.39662800<br>O -3.26572900 -3.03322800 0.81197400<br>O 5.20007600 -0.55264800 -1.57068300<br>O -2.40502300 -3.84708900 -1.55876400<br>C -0.36452800 2.02634300 1.06673800<br>C -1.11225400 2.37472700 -0.24836700<br>C 0.44161900 0.76474500 1.11555400<br>C 0.41237100 3.33030700 1.35899800<br>C -2.49614300 1.73598000 -0.45781200<br>C -1.20536500 3.90111600 -0.19397000	

C	1.65418500	0.46681400	0.43980100
C	-2.47038300	0.25070800	-0.75099200
C	2.40331100	-0.69858900	0.79676400
C	2.17771100	1.26840000	-0.61093100
C	-2.89908600	-0.67251400	0.21312000
C	-2.02951900	-0.23192000	-1.98366200
C	3.56561900	-1.03059900	0.12977500
C	3.34909400	0.92536300	-1.26798300
C	-2.87645800	-2.03627900	-0.05313800
C	-2.00335300	-1.59922300	-2.25613000
C	4.05432600	-0.22164400	-0.91906600
C	-2.42462900	-2.51009500	-1.29747500
C	3.97179900	-3.01281600	1.42638400
C	-3.80422600	-2.66109300	2.07459000
H	-1.12561100	1.91402700	1.84481400
H	-0.48658000	2.13290200	-1.11365500
H	0.46979100	3.55217100	2.42424100
H	1.42057400	3.32435600	0.94332500
H	-2.97197800	2.27294600	-1.28363500
H	-3.11060100	1.92826000	0.42761600
H	0.07703000	-1.05084700	1.62990500
H	2.09082900	-1.29405000	1.64363200
H	1.65594500	2.15719800	-0.93487900
H	-3.24382300	-0.31258400	1.17360300
H	-1.70784800	0.46380600	-2.75146300
H	3.73425500	1.53989900	-2.07280600
H	-1.66602800	-1.97613900	-3.21414900
H	5.52947400	-1.37021200	-1.17377900
H	-2.76620400	-4.29308500	-0.78125800
H	2.99388500	-3.45661100	1.21180800
H	3.94214500	-2.51101800	2.39924500
H	4.72810700	-3.79541500	1.44661800
H	-3.07115900	-2.10824800	2.67170000
H	-4.05580100	-3.59178300	2.58053300
H	-4.70846200	-2.05482700	1.95625900

Frequency and Energy at B3LYP/6-311G(d,p)in gas phase

Zero-point correction= 0.381620 (Hartree/Particle)
Thermal correction to Energy= 0.407209
Thermal correction to Enthalpy= 0.408154
Thermal correction to Gibbs Free Energy= 0.323096
Sum of electronic and zero-point Energies= -1301.244050
Sum of electronic and thermal Energies= -1301.218461
Sum of electronic and thermal Enthalpies= -1301.217517
Sum of electronic and thermal Free Energies= -1301.302574

Energy at ROB3LYP/6-311++G(2df,2p): in GAS PHASE	HF=-1301.7327597
**Name of anion**	**Hydroxymatairesinol-O4-H**

Cartesian Coordinates optimized at B3LYP/6-311G(d,p)

-1 1

O	-3.61981300	1.74232100	1.37938900
O	-1.15111000	1.68456300	-2.00191300
O	-5.24911400	1.08731100	-0.01595900
O	3.69837000	0.57128200	-0.30577500

O	0.62580400	-3.92634000	1.59251800
O	4.43765400	2.91054300	0.62603300
O	2.05555900	-3.58670000	-0.90019600
C	-1.68399500	0.93939600	0.23618400
C	-2.94449700	0.31642600	-0.38172000
C	-1.04056500	2.05825700	-0.61950400
C	-2.21045200	1.48006500	1.57326600
C	-3.07064300	-1.23067800	-0.13483500
C	-4.08614600	1.06122000	0.28402400
C	0.41143700	2.29993100	-0.25201000
C	-1.78450700	-1.97940600	-0.39161800
C	1.34814300	1.26611000	-0.45052900
C	0.84690500	3.52881600	0.23462300
C	-1.11364800	-2.63483900	0.65575000
C	-1.12715500	-1.93855500	-1.62868500
C	2.68731200	1.47557500	-0.15905400
C	2.19723600	3.74213900	0.53601400
C	0.13645400	-3.21532500	0.50121200
C	0.14017500	-2.48154000	-1.79357900
C	3.11648700	2.72563600	0.34125700
C	0.87423400	-3.13973500	-0.74497500
C	3.35902300	-0.79005900	-0.64604500
C	1.94257000	-3.59125800	2.01603100
H	-0.94019000	0.16193300	0.40226500
H	-2.98300200	0.49289900	-1.45460600
H	-1.59967800	2.99332700	-0.46212000
H	-2.10559700	0.74701400	2.37743400
H	-1.74098600	2.41436600	1.88647000
H	-3.89874500	-1.57856100	-0.76301700
H	-3.38061000	-1.40791300	0.90142900
H	-0.42760600	2.11203900	-2.46958400
H	1.01513600	0.30840200	-0.83427800
H	0.13360900	4.33366300	0.37947600
H	-1.57664500	-2.71423500	1.63742100
H	-1.60106100	-1.44118600	-2.47214300
H	2.54702500	4.69320700	0.92058000
H	0.63095400	-2.44959900	-2.76325300
H	4.86916600	2.06554800	0.43226800
H	2.90158900	-0.84981300	-1.63463100
H	2.67731900	-1.22061600	0.08506400
H	4.29634400	-1.34010700	-0.64299700
H	2.65076400	-3.72027700	1.19614900
H	2.17932400	-4.26337500	2.84581700
H	1.98388000	-2.55362000	2.38462500

Frequency and Energy at B3LYP/6-311G(d,p)in gas phase

Zero-point correction=	0.380339 (Hartree/Particle)
Thermal correction to Energy=	0.405628
Thermal correction to Enthalpy=	0.406572
Thermal correction to Gibbs Free Energy=	0.324470
Sum of electronic and zero-point Energies=	-1301.331402
Sum of electronic and thermal Energies=	-1301.306113
Sum of electronic and thermal Enthalpies=	-1301.305169
Sum of electronic and thermal Free Energies=	-1301.387270

**Name of anion**	**Hydroxymatairesinol-O4’-H**
Cartesian Coordinates optimized at B3LYP/6-311G(d,p)	

-1 1

O	0.16359600	3.88013500	0.73466400
O	0.20165600	0.46784700	-1.90642700
O	-1.63480100	4.62448600	-0.37476000
O	3.39774200	-2.55405100	0.97102000
O	-2.70384000	-2.76923300	-1.26583600
O	5.88414300	-1.43514000	0.10089900
O	-4.78966400	-3.34362800	0.25040500
C	0.07977200	1.54188500	0.24223200
C	-1.25260800	2.20874400	-0.16102200
C	0.99960300	1.25282500	-0.96280600
C	0.71179400	2.60394500	1.15592700
C	-2.48215500	1.85199000	0.69930200
C	-0.97155500	3.70104400	0.01522900
C	2.29012600	0.57175100	-0.62439800
C	-3.06151600	0.45299900	0.55132300
C	2.32434500	-0.65278600	0.08327500
C	3.52400000	1.09403700	-1.04674900
C	-2.54662900	-0.49324400	-0.34591100
C	-4.16271000	0.09918400	1.33492800
C	3.50247000	-1.32229000	0.33900700
C	4.71877300	0.44544600	-0.78456200
C	-3.12493100	-1.75547600	-0.43777600
C	-4.74779600	-1.16261700	1.23723100
C	4.80251500	-0.81959100	-0.09778000
C	-4.23350600	-2.09864000	0.35213000
C	4.22285200	-2.72184400	2.12200800
C	-1.51334700	-2.56666000	-2.02657400
H	-0.08124400	0.61189500	0.78869900
H	-1.48595500	2.03140000	-1.21173600
H	1.23294400	2.21294800	-1.44702900
H	0.45497600	2.46030200	2.20893600
H	1.79477600	2.65531300	1.05385900
H	-3.26364300	2.58180200	0.45991400
H	-2.23691000	2.01654800	1.75689900
H	0.85157700	-0.06848200	-2.37346100
H	1.40805400	-1.11892500	0.43804800
H	3.53609700	2.03389200	-1.59770500
H	-1.68799700	-0.25126700	-0.95970100
H	-4.57488800	0.81907000	2.03528000
H	5.66223500	0.85936400	-1.12897400
H	-5.60098500	-1.43790100	1.84654900
H	-4.25241300	-3.82594200	-0.39267300
H	3.92248500	-2.02741100	2.92138800
H	5.27069100	-2.55928100	1.86524400
H	4.06140000	-3.74654900	2.46769700
H	-1.63827000	-1.74923700	-2.74296300
H	-1.33916400	-3.50086800	-2.55892200
H	-0.66140000	-2.34419800	-1.37913400

Frequency and Energy at B3LYP/6-311G(d,p)in gas phase	
Zero-point correction= 0.380423 (Hartree/Particle)<br>Thermal correction to Energy= 0.405834<br>Thermal correction to Enthalpy= 0.406779<br>Thermal correction to Gibbs Free Energy= 0.322775<br>Sum of electronic and zero-point Energies= -1301.336574<br>Sum of electronic and thermal Energies= -1301.311163<br>Sum of electronic and thermal Enthalpies= -1301.310219<br>Sum of electronic and thermal Free Energies= -1301.394222	
Name of anion	**Hydroxymatairesinol-C7'-H**
Cartesian Coordinates optimized at B3LYP/6-311G(d,p	

-1 1

O	0.30078400	4.35192100	-0.76948100
O	0.14025800	-0.35929100	-1.91022300
O	1.67525200	4.61243800	0.97274500
O	-4.19222400	-2.30520000	-0.12740000
O	3.07407900	-2.84689900	-1.03743700
O	-5.29388900	-0.51218000	1.52608400
O	2.82342900	-3.82828500	1.41672900
C	0.38075000	1.95121000	-1.09571200
C	0.99547700	2.35039600	0.29379100
C	-0.48967600	0.74609300	-1.24762900
C	-0.32551400	3.26224300	-1.50975800
C	2.39694800	1.80210900	0.63692100
C	1.04825500	3.87387300	0.25285500
C	-1.66945300	0.47606000	-0.54147400
C	2.49741300	0.30903800	0.85555400
C	-2.33106000	-0.80023800	-0.69033900
C	-2.31587500	1.37862800	0.37869000
C	2.75984400	-0.54564800	-0.22575900
C	2.35589600	-0.25715200	2.12301600
C	-3.50295800	-1.09558600	-0.01821000
C	-3.49779900	1.04712800	1.03275700
C	2.85588900	-1.91788400	-0.04056800
C	2.46658100	-1.63523600	2.31760900
C	-4.11603400	-0.18332600	0.85364300
C	2.71837800	-2.47388500	1.24175300
C	-3.63155800	-3.31116600	-0.94209800
C	3.05249800	-2.38901800	-2.38810100
H	1.22482000	1.81058800	-1.78159700
H	0.29497100	2.06488000	1.08719500
H	-0.20826200	3.48267000	-2.57138300
H	-1.38561300	3.25123000	-1.26081700
H	2.74077300	2.32709500	1.53270600
H	3.08245300	2.09243900	-0.16885100
H	0.41158600	-0.98324400	-1.21783500
H	-1.90836500	-1.50483000	-1.39177800
H	-1.88453100	2.34818700	0.59214000
H	2.85742900	-0.12422200	-1.21615200
H	2.15254700	0.38377800	2.97423800
H	-3.95420500	1.75364800	1.72051000
H	2.35045900	-2.07582000	3.30103700

H	-5.49464400	-1.41394100	1.25047800
H	2.94161400	-4.20437700	0.53449700
H	-2.62455900	-3.58706400	-0.60615400
H	-3.57427300	-2.99774500	-1.99200900
H	-4.29139800	-4.17731900	-0.86077500
H	2.14021300	-1.82515100	-2.60438300
H	3.09355300	-3.28444600	-3.00787500
H	3.92591200	-1.76220600	-2.60297500

Frequency and Energy at B3LYP/6-311G(d,p)in gas phase

Zero-point correction= 0.378481 (Hartree/Particle)

Thermal correction to Energy= 0.404279

Thermal correction to Enthalpy= 0.405223

Thermal correction to Gibbs Free Energy= 0.321226

Sum of electronic and zero-point Energies= -1301.276318

Sum of electronic and thermal Energies= -1301.250520

Sum of electronic and thermal Enthalpies= -1301.249576

Sum of electronic and thermal Free Energies= -1301.333573

**Name of cationic radical**	**Hydroxymatairesinol**

Cartesian Coordinates optimized at B3LYP/6-311G(d,p

1 2

O	-0.73070800	3.56893800	-0.72423000
O	-0.86396500	0.13742900	1.91759000
O	0.86924100	4.49222100	0.55809500
O	-4.22951600	-2.92071300	-0.52469900
O	5.74658900	-1.44209300	-0.91147100
O	-6.31836400	-1.36901100	-0.92405800
O	4.85895500	-2.96900500	1.03514500
C	-0.51795200	1.27345500	-0.13843000
C	0.69315100	2.05395400	0.41512500
C	-1.54416200	0.98427600	0.98523300
C	-1.05541500	2.24870400	-1.20108400
C	2.05745400	1.76894700	-0.23546900
C	0.32688900	3.51889300	0.13002000
C	-2.82422000	0.34158700	0.48712300
C	2.75302800	0.47096600	0.10042100
C	-2.86513300	-1.03441700	0.23336800
C	-3.97705600	1.12263400	0.27362400
C	3.90756000	0.14120600	-0.62495000
C	2.32046200	-0.39109200	1.13995700
C	-4.03417000	-1.61551800	-0.23995000
C	-5.14789100	0.55656800	-0.20283700
C	4.62409300	-1.00473300	-0.31871000
C	3.01898900	-1.54179800	1.45124800
C	-5.19230400	-0.81264000	-0.46888200
C	4.17651300	-1.86663500	0.73820700
C	-3.16320500	-3.85797200	-0.32549300
C	6.34349400	-0.67644500	-1.97437600

H	-0.23596100	0.31996300	-0.59175300
H	0.78155700	1.94655300	1.49667200
H	-1.80199700	1.93732400	1.46613800
H	-0.56844300	2.09726700	-2.16820000
H	-2.13268000	2.20574700	-1.34763700
H	2.72308100	2.58855000	0.06655000
H	1.98059800	1.85132800	-1.32681500
H	-1.41227000	0.04354500	2.70482200
H	-1.98870600	-1.63473700	0.43508600
H	-3.95367300	2.18351400	0.49635700
H	4.24429100	0.79758100	-1.41713700
H	1.41801200	-0.16044900	1.69088600
H	-6.04149600	1.14511400	-0.36660700
H	2.69870900	-2.20582100	2.24396000
H	-6.17518900	-2.32078200	-1.04206700
H	5.63398500	-3.03171900	0.45349000
H	-2.86437500	-3.88056800	0.72556100
H	-2.30820300	-3.60561800	-0.95806800
H	-3.56449200	-4.82535500	-0.61605600
H	5.65278200	-0.59551400	-2.81663100
H	7.22862200	-1.23202600	-2.27141300
H	6.62725900	0.31433600	-1.61298100

Frequency and Energy at B3LYP/6-311G(d,p)in gas phase

Zero-point correction= 0.395023 (Hartree/Particle)
Thermal correction to Energy= 0.420505
Thermal correction to Enthalpy= 0.421450
Thermal correction to Gibbs Free Energy= 0.336414
Sum of electronic and zero-point Energies= -1301.614345
Sum of electronic and thermal Energies= -1301.588863
Sum of electronic and thermal Enthalpies= -1301.587919
Sum of electronic and thermal Free Energies= -1301.672955

**Name of compound (6)**	**Nortrachelogenin**

Cartesian Coordinates optimized at B3LYP/6-311G(d,p)

0 1

O	-0.60627700	3.83360400	-0.41080900
O	1.53515500	2.13485300	1.27719100
O	1.49848400	4.58434100	-0.61235400
O	-5.21308800	-0.65777800	1.50670600
O	4.35404800	-2.06171100	1.48709600
O	-5.46684900	-2.32014500	-0.71572500
O	3.85069000	-3.68041100	-0.54128800
C	-0.23501000	1.47551300	-0.34549800
C	1.10336100	2.20575600	-0.08908700
C	-1.27279100	2.58682300	-0.11206000
C	-0.49005000	0.20131800	0.47935900
C	2.29426000	1.77319300	-0.95704400
C	0.74410500	3.67862000	-0.41082700
C	-1.80791400	-0.46945400	0.14841000
C	2.70585400	0.32286700	-0.84351000
C	-2.94612600	-0.25954900	0.93282300
C	-1.92593600	-1.30817700	-0.96208500

C 3.34865300 -0.14915100 0.31411800<br>C 2.47230100 -0.57028400 -1.88754200<br>C -4.16890200 -0.86022700 0.64291700<br>C 3.72403200 -1.48063300 0.41394600<br>C -3.13982900 -1.91778300 -1.26744000<br>C 2.85044700 -1.91217600 -1.79124100<br>C -4.26542700 -1.71042000 -0.47233800<br>C 3.47404800 -2.37697700 -0.64442000<br>C -6.38950300 -0.04540000 0.96414400<br>C 4.66254800 -1.24803500 2.61251300<br>H -0.25929600 1.21513900 -1.40899900<br>H -2.14476600 2.51475200 -0.75938500<br>H -1.61714700 2.62441600 0.92731100<br>H -0.46350300 0.45005800 1.54371100<br>H 0.33266500 -0.49440900 0.30502000<br>H 3.12029100 2.43692300 -0.68709300<br>H 2.04635000 1.99763900 -1.99798700<br>H 0.84994200 2.48963600 1.85360600<br>H -2.90543100 0.36634700 1.81784600<br>H -1.05996400 -1.50613700 -1.58438100<br>H 3.52957400 0.53753500 1.12908100<br>H 1.99828400 -0.22011800 -2.79839600<br>H -3.21205900 -2.58324900 -2.12357600<br>H 2.67705500 -2.60697000 -2.60424600<br>H -5.37745200 -2.88954200 -1.48685000<br>H 4.26890300 -3.78692100 0.32368800<br>H -7.06433100 0.10038100 1.80694700<br>H -6.86476800 -0.68344600 0.21751600<br>H -6.14855000 0.92774500 0.52119500<br>H 3.75497500 -0.82367500 3.05396100<br>H 5.34746700 -0.43868800 2.33935300<br>H 5.14573100 -1.90437600 3.33431500	
Frequency and Energy at B3LYP/6-311G(d,p)in gas phase	
Zero-point correction= 0.393133 (Hartree/Particle)<br>Thermal correction to Energy= 0.419219<br>Thermal correction to Enthalpy= 0.420163<br>Thermal correction to Gibbs Free Energy= 0.334386<br>Sum of electronic and zero-point Energies= -1301.863830<br>Sum of electronic and thermal Energies= -1301.837743<br>Sum of electronic and thermal Enthalpies= -1301.836799<br>Sum of electronic and thermal Free Energies= -1301.922576	
Energy at ROB3LYP/6-311++G(2df,2p): in gas phase	HF=-1302.3655569
Energy at ROB3LYP/6-311++G(2df,2p): in H2O	HF=-1302.3885278
Energy at ROB3LYP/6-311++G(2df,2p): in ETHANOL	HF-1302.3873787
**Name of radical**	**Nortrachelogenin-O4-H**
Cartesian Coordinates optimized at B3LYP/6-311G(d,p)	
0 2<br>O -0.54424800 3.78315400 -0.42601600<br>O 1.56152400 2.05367600 1.25223400<br>O 1.57182200 4.49551700 -0.65724700<br>O -5.20289700 -0.59872400 1.54605000<br>O 4.42942300 -2.02439500 1.53311100	

O	-5.55105200	-2.22436900	-0.69326000
O	3.90390500	-3.68717500	-0.49934900
C	-0.21019700	1.41959000	-0.37691500
C	1.13796700	2.12872900	-0.11455200
C	-1.22830700	2.54543700	-0.12562600
C	-0.48282100	0.13952400	0.43286600
C	2.33168800	1.68621900	-0.97640900
C	0.80158400	3.60678800	-0.44223700
C	-1.82473400	-0.49074700	0.11829600
C	2.75335500	0.24524800	-0.83549800
C	-2.94000800	-0.25889700	0.92898200
C	-1.98909200	-1.31007100	-1.00040300
C	3.40910600	-0.19369200	0.32668000
C	2.50949300	-0.67181100	-1.88831900
C	-4.18556900	-0.81960200	0.65668300
C	3.80470500	-1.51578800	0.46447600
C	-3.22609700	-1.88035700	-1.28844300
C	2.88790000	-1.98503800	-1.78526900
C	-4.32890200	-1.65195000	-0.46755600
C	3.55569400	-2.50011800	-0.60779400
C	-6.38249600	0.03788100	1.03832700
C	4.72370100	-1.16748000	2.63453400
H	-0.24425500	1.17608300	-1.44434100
H	-2.10766100	2.49055900	-0.76412300
H	-1.56071600	2.58309000	0.91734200
H	-0.42816600	0.37089300	1.49978600
H	0.31623700	-0.57737200	0.23367100
H	3.15536000	2.35467200	-0.70965100
H	2.08553100	1.89464400	-2.02056200
H	0.88184500	2.42309100	1.82618200
H	-2.86312600	0.35165700	1.82223800
H	-1.14268700	-1.52597800	-1.64341600
H	3.58229200	0.52180000	1.11791400
H	2.02365800	-0.31653600	-2.79148200
H	-3.33439800	-2.53260600	-2.15077900
H	2.71437700	-2.69721000	-2.58343000
H	-5.49275200	-2.79313800	-1.46790000
H	-7.02675500	0.19995300	1.90168800
H	-6.89442600	-0.59113800	0.30876700
H	-6.13430200	1.00458800	0.58553200
H	3.80837500	-0.74545900	3.06069600
H	5.39650600	-0.35798400	2.33496000
H	5.21543300	-1.79825100	3.37177700

Frequency and Energy at B3LYP/6-311G(d,p)in gas phase

Zero-point correction=	0.380050 (Hartree/Particle)
Thermal correction to Energy=	0.405972
Thermal correction to Enthalpy=	0.406917
Thermal correction to Gibbs Free Energy=	0.320295
Sum of electronic and zero-point Energies=	-1301.233731
Sum of electronic and thermal Energies=	-1301.207809
Sum of electronic and thermal Enthalpies=	-1301.206865
Sum of electronic and thermal Free Energies=	-1301.293486

Energy at ROB3LYP/6-311++G(2df,2p): in GAS PHASE			HF=-1301.7187569
**Name of radical**		**Nortrachelogenin-O4'-H**	
Cartesian Coordinates optimized at B3LYP/6-311G(d,p)			
0 2			
O	-0.44591300	3.91834000	-0.30209300
O	1.57458000	2.05072100	1.34815900
O	1.69671500	4.56976400	-0.44808500
O	-5.16876800	-0.53508400	1.49442800
O	4.19363300	-2.27270400	1.45834700
O	-5.50727800	-1.88358000	-1.05367900
O	3.62552000	-3.80265100	-0.62166900
C	-0.19233800	1.54641200	-0.32925300
C	1.17673900	2.19724900	-0.02247700
C	-1.17808800	2.69630200	-0.06170300
C	-0.51547800	0.25540400	0.44587900
C	2.35886700	1.73435100	-0.88677600
C	0.89521100	3.69652700	-0.29262900
C	-1.85730800	-0.32859800	0.07412300
C	2.69508700	0.26185800	-0.81360800
C	-2.95223000	-0.21005400	0.92079400
C	-2.01700600	-1.00318500	-1.17107900
C	3.30034900	-0.27691300	0.33516300
C	2.42595700	-0.58678300	-1.88603600
C	-4.20839400	-0.72308800	0.58149200
C	3.60475600	-1.62870200	0.39838400
C	-3.22626600	-1.51695100	-1.53978500
C	2.73187200	-1.94903600	-1.82626600
C	-4.40341800	-1.41157400	-0.70114400
C	3.31856100	-2.47947000	-0.68836700
C	-6.52291500	-0.99899000	1.32716000
C	4.55004700	-1.50650700	2.60284600
H	-0.21166700	1.33181100	-1.40279400
H	-2.04369100	2.69576400	-0.72181200
H	-1.53367200	2.70774700	0.97435400
H	-0.48499800	0.46244100	1.51779300
H	0.27191000	-0.47422300	0.24294400
H	3.21190100	2.34691500	-0.58260500
H	2.13919200	2.00347200	-1.92346200
H	0.94696500	2.50342000	1.92162900
H	-2.85928100	0.28115200	1.88326000
H	-1.15520700	-1.11724200	-1.82038100
H	3.50941100	0.37473900	1.17191900
H	1.98315600	-0.18552400	-2.79155300
H	-3.36681900	-2.03925300	-2.47876500
H	2.53104600	-2.60949400	-2.66119400
H	4.03304200	-3.95738700	0.24112900
H	-7.02539500	-0.68872000	2.24217100
H	-6.55425000	-2.08186400	1.21668700
H	-6.98811300	-0.54106800	0.45561200
H	3.66875900	-1.04359100	3.05866100
H	5.27826300	-0.73007200	2.34694300
H	4.99750400	-2.20696800	3.30588000

Frequency and Energy at B3LYP/6-311G(d,p)in gas phase	
Zero-point correction= 0.380868 (Hartree/Particle)<br>Thermal correction to Energy= 0.406530<br>Thermal correction to Enthalpy= 0.407474<br>Thermal correction to Gibbs Free Energy= 0.321667<br>Sum of electronic and zero-point Energies= -1301.244912<br>Sum of electronic and thermal Energies= -1301.219250<br>Sum of electronic and thermal Enthalpies= -1301.218306<br>Sum of electronic and thermal Free Energies= -1301.304114	
Energy at ROB3LYP/6-311++G(2df,2p): in GAS PHASE	HF=-1301.7298524
Energy at ROB3LYP/6-311++G(2df,2p): in H2O	HF=-1301.7525299
Energy at ROB3LYP/6-311++G(2df,2p): in ETHANOL	HF=-1301.7513976
**Name of radical**	**Nortrachelogenin-O8-H**
Cartesian Coordinates optimized at B3LYP/6-311G(d,p)	

0 2

O	-0.63952200	3.77629900	-0.20679700
O	1.35538000	2.09028000	1.23826200
O	1.44228600	4.53552700	-0.57527200
O	-5.10528600	-0.86312500	1.62910100
O	4.36919500	-1.94238600	1.60806500
O	-5.66720100	-2.11094700	-0.79305700
O	4.16915000	-3.56812900	-0.46625600
C	-0.27710500	1.43483100	-0.41434900
C	1.09628800	2.09968400	-0.07318300
C	-1.26788000	2.49368000	0.06717900
C	-0.49028100	0.05338900	0.23082700
C	2.30123700	1.77346500	-0.98649100
C	0.68186400	3.65852200	-0.32124600
C	-1.85955700	-0.52718300	-0.05431600
C	2.79889300	0.35297700	-0.85800000
C	-2.89666400	-0.42531600	0.87874800
C	-2.13281700	-1.16268700	-1.26792100
C	3.35700800	-0.08764200	0.35455800
C	2.71928500	-0.54158600	-1.92292000
C	-4.16738100	-0.93911300	0.63349600
C	3.80805900	-1.39232200	0.48427300
C	-3.39763800	-1.68130000	-1.53161500
C	3.17781700	-1.85619000	-1.79780900
C	-4.42052500	-1.58436400	-0.59009400
C	3.72031700	-2.29134000	-0.59973900
C	-6.29513600	-0.12044800	1.33588400
C	4.50079000	-1.12727600	2.76816500
H	-0.35203300	1.35047000	-1.50441900
H	-2.21807700	2.49198100	-0.46181500
H	-1.43976300	2.43211900	1.14310400
H	-0.33998300	0.15505300	1.30862300
H	0.28229100	-0.62564500	-0.13527700
H	3.08455600	2.48664900	-0.71605400
H	2.02943800	1.97732100	-2.02600800
H	-2.73186000	0.04091800	1.84412800
H	-1.34926800	-1.27245800	-2.01001600
H	3.41551700	0.60329600	1.18481000

H 2.30351700 -0.21509300 -2.87023000
H -3.59081600 -2.18882900 -2.47293300
H 3.12411300 -2.55328600 -2.62535400
H -5.68711000 -2.54636300 -1.65147800
H 4.50747400 -3.65780800 0.43488500
H -6.87729300 -0.11542000 2.25670700
H -6.87276700 -0.58906700 0.53750700
H -6.04749300 0.91070700 1.05905900
H 3.52452200 -0.77571500 3.11647600
H 5.14991600 -0.26745900 2.57398500
H 4.95367000 -1.76019500 3.52925700

Frequency and Energy at B3LYP/6-311G(d,p)in gas phase

Zero-point correction= 0.379909 (Hartree/Particle)
Thermal correction to Energy= 0.405567
Thermal correction to Enthalpy= 0.406511
Thermal correction to Gibbs Free Energy= 0.321042
Sum of electronic and zero-point Energies= -1301.213457
Sum of electronic and thermal Energies= -1301.187799
Sum of electronic and thermal Enthalpies= -1301.186855
Sum of electronic and thermal Free Energies= -1301.272324

Energy at ROB3LYP/6-311++G(2df,2p): in GAS PHASE	HF=-1301.698279
**Name of anion**	**Nortrachelogenin-O4’-H**

Cartesian Coordinates optimized at B3LYP/6-311G(d,p

-1 1
O -0.76836900 3.61027200 -0.32109600
O 1.33454900 1.89570500 1.12943500
O 1.31394300 4.33166300 -0.72584700
O -5.31782600 -0.75179100 1.75368600
O 5.10340500 -1.41406500 1.43842400
O -5.96952200 -2.04074500 -0.72640900
O 4.57947300 -3.45597800 -0.16363900
C -0.44465600 1.25273100 -0.46113700
C 0.91940200 1.94515800 -0.24565800
C -1.42467000 2.34604100 -0.01861600
C -0.69768000 -0.10053500 0.23733400
C 2.07437900 1.55277700 -1.18960400
C 0.55796000 3.43547000 -0.46451700
C -2.08942400 -0.64163700 -0.00770000
C 2.72201200 0.21354300 -0.92068800
C -3.12210900 -0.46645500 0.93194400
C -2.42788000 -1.28822000 -1.20453800
C 3.60791600 0.06850300 0.16163900
C 2.48238700 -0.89417200 -1.73270400
C -4.41298300 -0.91379100 0.70928900
C 4.21901200 -1.15205100 0.41367300
C -3.71810100 -1.73361100 -1.45203300
C 3.09718700 -2.12259200 -1.48291100
C -4.80392300 -1.59857300 -0.51377500
C 3.96681300 -2.26155300 -0.41261600
C -6.54704800 -0.12775200 1.39428200
C 5.39422600 -0.37003200 2.35596000
H -0.57075000 1.10636100 -1.53966900

H	-2.37103100	2.32815900	-0.55269200
H	-1.61588900	2.31395400	1.05607000
H	-0.55542500	0.02539000	1.31935000
H	0.05865500	-0.81633200	-0.10363700
H	2.81686200	2.35128200	-1.10027100
H	1.70390300	1.58295100	-2.21853700
H	1.25592800	0.97657200	1.40840600
H	-2.92280800	0.01449600	1.88835300
H	-1.65575200	-1.45687100	-1.95591300
H	3.80238100	0.92487200	0.79295500
H	1.80336500	-0.80549600	-2.57335200
H	-3.95631700	-2.25485100	-2.37551600
H	2.90646100	-2.98446300	-2.11106300
H	5.11355600	-3.33277200	0.63193200
H	-7.16881100	-0.12980900	2.29452700
H	-7.03031700	-0.68076300	0.58665900
H	-6.37954900	0.91561300	1.08323300
H	4.48987600	-0.02920800	2.87112500
H	5.86504400	0.48195100	1.85362500
H	6.08847500	-0.79101500	3.08223900

Frequency and Energy at B3LYP/6-311G(d,p)in gas phase

Zero-point correction= 0.379286 (Hartree/Particle)
Thermal correction to Energy= 0.404840
Thermal correction to Enthalpy= 0.405784
Thermal correction to Gibbs Free Energy= 0.320744
Sum of electronic and zero-point Energies= -1301.324601
Sum of electronic and thermal Energies= -1301.299048
Sum of electronic and thermal Enthalpies= -1301.298103
Sum of electronic and thermal Free Energies= -1301.383144

**Name of cationic radical**	**Nortrachelogenin**

Cartesian Coordinates optimized at B3LYP/6-311G(d,p

1 2

O	-0.37565200	3.91691900	-0.27138400
O	1.42539900	1.93775600	1.35837600
O	1.79642900	4.50411400	-0.27702500
O	-5.07842500	-0.66451200	1.64367200
O	4.33809500	-2.23688200	1.42853800
O	-5.73295900	-1.70671400	-0.95543500
O	3.78831700	-3.76418200	-0.64082100
C	-0.18149800	1.54867100	-0.39548900
C	1.18953700	2.14567800	-0.03767700
C	-1.13309200	2.70613300	-0.04744700
C	-0.52891200	0.21850300	0.29653200
C	2.39829300	1.70708200	-0.89423700
C	0.94527400	3.66422200	-0.22955700
C	-1.91002000	-0.29407200	-0.03360800
C	2.75876000	0.24816800	-0.80179200
C	-2.92293500	-0.28297900	0.90926600
C	-2.19998500	-0.80751600	-1.32148100
C	3.38564700	-0.26508300	0.34147400
C	2.49393400	-0.62174800	-1.88631500

C	-4.22015600	-0.74708600	0.62645900
C	3.73127200	-1.60759400	0.40441400
C	-3.46432400	-1.27108600	-1.63229100
C	2.83197400	-1.96197700	-1.84029000
C	-4.49175200	-1.25365100	-0.68370900
C	3.45193200	-2.47476200	-0.69852600
C	-6.47345200	-1.03677200	1.58897200
C	4.71141600	-1.49439300	2.60122700
H	-0.20125300	1.41367500	-1.48264300
H	-2.01752400	2.75503400	-0.67954300
H	-1.43570400	2.68468700	1.00241500
H	-0.42596900	0.34810900	1.37434300
H	0.21125400	-0.53025600	-0.00703700
H	3.24144900	2.33124800	-0.58075900
H	2.19603500	1.97219100	-1.93536600
H	2.04339300	2.61540100	1.66205700
H	-2.75135800	0.08695400	1.91285400
H	-1.42025900	-0.85182800	-2.07288500
H	3.59144300	0.38997200	1.17494300
H	2.03602800	-0.22151100	-2.78345600
H	-3.67385100	-1.66687200	-2.62104400
H	2.64693100	-2.62916600	-2.67243700
H	-5.78535000	-2.02245000	-1.86645700
H	4.22297300	-3.93954000	0.20890700
H	-6.85022100	-0.80652200	2.58205900
H	-6.58015600	-2.09998700	1.37828400
H	-6.99970800	-0.45002900	0.83702000
H	3.82636100	-1.06791400	3.07914800
H	5.42271600	-0.70703900	2.34137200
H	5.18166100	-2.21457100	3.26530600

Frequency and Energy at B3LYP/6-311G(d,p)in gas phase

Zero-point correction=	0.394033 (Hartree/Particle)
Thermal correction to Energy=	0.419749
Thermal correction to Enthalpy=	0.420693
Thermal correction to Gibbs Free Energy=	0.335193
Sum of electronic and zero-point Energies=	-1301.609148
Sum of electronic and thermal Energies=	-1301.583432
Sum of electronic and thermal Enthalpies=	-1301.582488
Sum of electronic and thermal Free Energies=	-1301.667988

**Name of compound (7)**	**Pinoresinol**

Cartesian Coordinates optimized at B3LYP/6-311G(d,p)

0 1

O	1.19315500	-2.17030200	0.13458700
O	-1.18367700	1.28471400	-0.80382500
O	6.14327200	1.15903300	-0.86793300
O	-5.92793900	1.38370300	1.08264300
O	6.92722800	0.18273600	1.46349600
O	-7.16977800	-0.64081500	-0.07965600
C	0.57781400	0.09352100	0.27858200
C	-0.71972800	-0.75098600	0.27882900
C	1.63756100	-0.88363200	-0.29382600

C	-1.57390600	-0.09090000	-0.83422500	
C	-0.23174100	-2.19226200	0.02627800	
C	0.22668500	1.32642600	-0.57838900	
C	3.05596900	-0.64140200	0.16173600	
C	-3.06842900	-0.21336400	-0.66855600	
C	3.91619400	0.13433600	-0.62859500	
C	-3.76298700	0.69912300	0.13900100	
C	3.51631800	-1.13906500	1.37833100	
C	-3.76742600	-1.25301700	-1.27480400	
C	5.20476800	0.41547900	-0.19236100	
C	-5.12891000	0.55614000	0.33133300	
C	4.81244200	-0.86458200	1.81362600	
C	-5.14098600	-1.40471000	-1.07369300	
C	5.66144800	-0.08561400	1.04044100	
C	-5.82913500	-0.50689400	-0.27252500	
C	5.80042700	1.69358500	-2.13885600	
C	-5.32837600	2.50316000	1.72208300	
H	0.86976900	0.37819400	1.28993400	
H	-1.24876500	-0.66892900	1.22897900	
H	1.60327200	-0.83470000	-1.39517900	
H	-1.28914500	-0.52802400	-1.80479700	
H	-0.60328000	-2.91127200	0.75677800	
H	-0.52446400	-2.53756800	-0.97593900	
H	0.45197700	2.27488500	-0.08956800	
H	0.75789900	1.30100600	-1.54125900	
H	3.57249500	0.50442800	-1.58669000	
H	-3.21852400	1.52358800	0.57779500	
H	2.86398900	-1.76241400	1.97599200	
H	-3.24613600	-1.95315000	-1.91879300	
H	5.18419900	-1.25439800	2.75370900	
H	-5.69426200	-2.20938500	-1.54258300	
H	7.34718200	0.72237600	0.78059500	
H	-7.44849700	0.08779200	0.49100300	
H	4.95708100	2.38855200	-2.06306900	
H	5.55541100	0.89843600	-2.85111500	
H	6.68058900	2.23159200	-2.48702100	
H	-4.87744200	3.18185800	0.99079100	
H	-4.56695700	2.18619600	2.44257000	
H	-6.13248500	3.01631600	2.24690900	
Frequency and Energy at B3LYP/6-311G(d,p)in gas phase				
Zero-point correction= 0.391564 (Hartree/Particle)<br>Thermal correction to Energy= 0.415124<br>Thermal correction to Enthalpy= 0.416068<br>Thermal correction to Gibbs Free Energy= 0.334852<br>Sum of electronic and zero-point Energies= -1226.596160<br>Sum of electronic and thermal Energies= -1226.572600<br>Sum of electronic and thermal Enthalpies= -1226.571656<br>Sum of electronic and thermal Free Energies= -1226.652872				
Energy at ROB3LYP/6-311++G(2df,2p): in gas phase				HF=-1227.0903209
Energy at ROB3LYP/6-311++G(2df,2p): in H2O				HF=-1227.1064914
Energy at ROB3LYP/6-311++G(2df,2p): in ETHANOL				HF=-1227.1056805
**Name of radical**		**Pinoresinol-O4-H**		

Cartesian Coordinates optimized at B3LYP/6-311G(d,p)			
0 2			
O	1.03693400	-2.04145300	-0.37554400
O	-1.11472400	1.18570300	-0.81309000
O	5.69887100	1.62456000	-0.88971900
O	-5.97104400	1.53895500	0.72531600
O	6.91595200	-0.23341500	0.97900800
O	-7.11776900	-0.72622200	-0.01252900
C	0.57285800	0.14038400	0.49634800
C	-0.69783800	-0.72188600	0.54670300
C	1.49417100	-0.68641300	-0.44755300
C	-1.50391100	-0.18925100	-0.66686400
C	-0.16590800	-2.14111100	0.40170700
C	0.08139700	1.46890500	-0.07480800
C	2.96144800	-0.58958000	-0.10249600
C	-3.00495300	-0.31090700	-0.52937600
C	3.73179100	0.43408000	-0.64146200
C	-3.75244800	0.73147300	0.03600300
C	3.55351100	-1.51482700	0.79909800
C	-3.65711100	-1.47494100	-0.92867300
C	5.07608000	0.59516600	-0.30065600
C	-5.12256200	0.59220200	0.20457600
C	4.86621600	-1.39104800	1.15315000
C	-5.03355000	-1.62188400	-0.74788600
C	5.71543900	-0.33507700	0.64089900
C	-5.77356300	-0.59632700	-0.17977600
C	7.09027700	1.93218600	-0.67960300
C	-5.43211000	2.80281800	1.09127200
H	1.04070300	0.26118400	1.47533100
H	-1.26602700	-0.58289600	1.46867000
H	1.35676700	-0.33283000	-1.47781200
H	-1.19157400	-0.73997000	-1.56498700
H	0.06212700	-2.57626400	1.38259500
H	-0.85387600	-2.81344600	-0.11674700
H	-0.15179400	2.17921000	0.72677900
H	0.80762400	1.94128600	-0.74346900
H	3.30904800	1.13361900	-1.35456300
H	-3.24610600	1.64921300	0.30085600
H	2.94930900	-2.33022500	1.17581600
H	-3.09805000	-2.27602600	-1.40003200
H	5.34484300	-2.08481300	1.83439700
H	-5.54991800	-2.52234200	-1.05765900
H	-7.43700400	0.10050600	0.37313100
H	7.72527900	1.10391400	-0.99094300
H	7.28801100	2.15490300	0.36774900
H	7.26629900	2.80837400	-1.30212900
H	-4.97041300	3.29926800	0.23151700
H	-4.69376000	2.70195500	1.89379400
H	-6.27264500	3.39657900	1.44655000
Frequency and Energy at B3LYP/6-311G(d,p)in gas phase			
Zero-point correction=	0.378258 (Hartree/Particle)		
Thermal correction to Energy=	0.400925		

Thermal correction to Enthalpy= 0.401869<br>Thermal correction to Gibbs Free Energy= 0.322703<br>Sum of electronic and zero-point Energies= -1225.969326<br>Sum of electronic and thermal Energies= -1225.946659<br>Sum of electronic and thermal Enthalpies= -1225.945715<br>Sum of electronic and thermal Free Energies= -1226.024880		
Energy at ROB3LYP/6-311++G(2df,2p): in GAS PHASE		HF=-1226.4446367
Energy at ROB3LYP/6-311++G(2df,2p): in H2O		HF=-1226.4615343
Energy at ROB3LYP/6-311++G(2df,2p): in ETHANOL		HF=-1226.4607073
**Name of radical**	**Pinoresinol-C2-H**	
Cartesian Coordinates optimized at B3LYP/6-311G(d,p		

0 2			
O	1.28962700	-1.44063100	-1.55683400
O	-1.36084300	1.47479100	-0.11166400
O	6.31771500	1.37970200	0.31606900
O	-6.44811400	1.25409700	0.39797000
O	6.88815100	-0.97487500	1.38019300
O	-7.06414200	-1.31694300	0.47142900
C	0.70161200	0.27289500	-0.00320900
C	-0.47295300	-0.72305600	-0.17810800
C	1.79351600	-0.21924200	-1.00662700
C	-1.69033800	0.13950100	-0.10063300
C	-0.12985900	-1.37217600	-1.54021700
C	0.05116000	1.62790800	-0.33234100
C	3.15938700	-0.44832800	-0.40136600
C	-3.03980900	-0.22940300	0.04377500
C	4.08425400	0.60589300	-0.37508600
C	-4.05962400	0.76506300	0.14711400
C	3.50751400	-1.67115900	0.16427700
C	-3.43529000	-1.59271800	0.09375100
C	5.32498100	0.43355400	0.22415800
C	-5.38011400	0.39527800	0.28828500
C	4.75631800	-1.84776500	0.76121600
C	-4.76857300	-1.94474700	0.23647100
C	5.66865300	-0.80385200	0.80058800
C	-5.75440400	-0.96722200	0.33400600
C	6.09161200	2.65943000	-0.25780900
C	-6.19515100	2.65210100	0.36268200
H	1.08372000	0.26708600	1.01689400
H	-0.46471500	-1.49560700	0.59574100
H	1.88874400	0.51890100	-1.81820600
H	-0.51467900	-2.38527900	-1.65778000
H	-0.50535600	-0.75437300	-2.36904300
H	0.39535200	2.44579800	0.30215000
H	0.20923500	1.90660600	-1.38104500
H	3.82829000	1.55345500	-0.83410000
H	-3.77019800	1.80503700	0.11286500
H	2.80718400	-2.49453300	0.11733900
H	-2.68923500	-2.37452700	0.01930300
H	5.04121300	-2.79768900	1.19744900
H	-5.07095700	-2.98459900	0.27448400

H	7.36646500	-0.14002300	1.29006900
H	-7.57068300	-0.49529000	0.52171600
H	5.24031300	3.16130200	0.21486900
H	5.91911200	2.58555200	-1.33694900
H	6.99682000	3.23613800	-0.07538000
H	-5.73592200	2.94688900	-0.58673800
H	-5.54620900	2.95750500	1.19017600
H	-7.16500700	3.13679100	0.46331800

Frequency and Energy at B3LYP/6-311G(d,p)in gas phase (HF= -1226.4532325)

Zero-point correction= 0.378440 (Hartree/Particle)
Thermal correction to Energy= 0.402110
Thermal correction to Enthalpy= 0.403054
Thermal correction to Gibbs Free Energy= 0.321944
Sum of electronic and zero-point Energies= -1225.974611
Sum of electronic and thermal Energies= -1225.950942
Sum of electronic and thermal Enthalpies= -1225.949998
Sum of electronic and thermal Free Energies= -1226.031108

**Name of anion**	**Pinoresinol-O4-H**

Cartesian Coordinates optimized at B3LYP/6-311G(d,p

-1 1

O	1.22527600	-1.95705400	0.53834300
O	-1.68003400	-1.17011200	-1.82001300
O	6.51591700	-0.54286200	0.20868500
O	-5.68012400	1.76109000	-0.58737500
O	6.09839600	2.15825800	1.07528700
O	-6.74430300	0.59245700	1.53523100
C	0.46128800	-0.43664200	-1.08425400
C	-0.52133700	-0.44969300	0.12365900
C	1.74796900	-1.11887400	-0.55923400
C	-1.73562600	-1.27244000	-0.39460400
C	0.29051000	-1.14657800	1.22851000
C	-0.29281000	-1.24624400	-2.15529600
C	2.86602800	-0.20208000	-0.16301100
C	-3.08408800	-0.81215700	0.10046700
C	4.19079400	-0.68637000	-0.17799300
C	-3.73000500	0.26002300	-0.53229400
C	2.69209500	1.12468600	0.26811500
C	-3.68750800	-1.40355700	1.20667500
C	5.27018100	0.07737900	0.21608200
C	-4.94479900	0.72414800	-0.05262700
C	3.76357400	1.91138300	0.66058100
C	-4.90960900	-0.93394600	1.69515600
C	5.12781700	1.44819400	0.68814200
C	-5.54447300	0.12879300	1.07260100
C	7.53661700	0.16092300	-0.49165700
C	-5.13282900	2.46557700	-1.69301600
H	0.66145100	0.57260300	-1.43961700
H	-0.84223600	0.55257600	0.41384800
H	2.12510600	-1.85645600	-1.27448500
H	-1.58513600	-2.32403000	-0.10631300
H	0.79980700	-0.40612300	1.85776300

H	-0.31403800	-1.79548600	1.86936800
H	-0.17703800	-0.85586700	-3.16814700
H	0.03423500	-2.29586000	-2.13950200
H	4.39281800	-1.70661200	-0.49796500
H	-3.26672900	0.69229800	-1.40840600
H	1.69549000	1.56220700	0.29363800
H	-3.20627200	-2.24293000	1.69679400
H	3.60597000	2.93317300	0.99586900
H	-5.38453100	-1.38888200	2.55647500
H	-6.99472900	1.32684600	0.95942400
H	7.65097200	1.16798700	-0.08621500
H	7.30690000	0.21707800	-1.56755800
H	8.45619900	-0.41717700	-0.35959900
H	-5.00228600	1.80768900	-2.55869400
H	-4.16965500	2.91921200	-1.43674000
H	-5.85005300	3.24839100	-1.93721500

Frequency and Energy at B3LYP/6-311G(d,p)in gas phase

Zero-point correction= 0.377430 (Hartree/Particle)
Thermal correction to Energy= 0.400527
Thermal correction to Enthalpy= 0.401471
Thermal correction to Gibbs Free Energy= 0.322595
Sum of electronic and zero-point Energies= -1226.046031
Sum of electronic and thermal Energies= -1226.022933
Sum of electronic and thermal Enthalpies= -1226.021989
Sum of electronic and thermal Free Energies= -1226.100865

Name of anion	**Pinoresinol-C2-H**

Cartesian Coordinates optimized at B3LYP/6-311G(d,p

-1 1

O	1.40531400	-1.95633600	-1.33674700
O	-1.27822000	1.33936000	-0.66121600
O	5.93954300	1.72649900	0.47550800
O	-6.25229700	1.46853500	0.51738100
O	6.85045700	-0.49882000	1.58746800
O	-7.01171200	-1.03303800	1.08461500
C	0.69438800	0.10475900	-0.35628500
C	-0.49149800	-0.90321700	-0.34160500
C	1.83946000	-0.61511600	-1.11499900
C	-1.69640200	-0.05612000	-0.63393300
C	-0.03146200	-1.93639000	-1.39496200
C	0.06103800	1.32538900	-1.05908800
C	3.17687600	-0.61554800	-0.40461200
C	-2.99788000	-0.29892600	-0.18636900
C	3.91155300	0.57842800	-0.33396000
C	-3.96399900	0.76027900	-0.04972400
C	3.69612900	-1.75987500	0.19203900
C	-3.46626400	-1.61019000	0.16340300
C	5.12787900	0.61584700	0.33339800
C	-5.25742600	0.49692500	0.36428400
C	4.92407400	-1.72418400	0.85850700
C	-4.77134400	-1.83533100	0.58161400
C	5.64536700	-0.54326900	0.93622900
C	-5.69276900	-0.79781400	0.68535800

C	5.48382300	2.95764400	-0.06015500
C	-5.91896500	2.80201600	0.20293800
H	0.99880100	0.36926800	0.65776200
H	-0.54617100	-1.42419400	0.63161500
H	1.97411800	-0.12463600	-2.09566500
H	-0.37376800	-2.95543000	-1.19904100
H	-0.37252800	-1.62543500	-2.39160200
H	0.52037600	2.27973100	-0.77532100
H	0.15199600	1.19857900	-2.15600300
H	3.51471400	1.47112200	-0.80265500
H	-3.64060900	1.76764700	-0.26924400
H	3.13048800	-2.67918300	0.12563200
H	-2.78920300	-2.45477200	0.08444000
H	5.33467800	-2.61149500	1.32697300
H	-5.10051700	-2.83933700	0.83613200
H	7.15970200	0.41397400	1.52411500
H	-7.42857400	-0.16377300	1.08155200
H	4.53292700	3.26184700	0.39094700
H	5.36295900	2.89999700	-1.14784400
H	6.25009200	3.69495300	0.17829500
H	-5.60407600	2.90512400	-0.84297300
H	-5.11383900	3.18070200	0.84495400
H	-6.82173300	3.39388300	0.36940300

Frequency and Energy at B3LYP/6-311G(d,p)in gas phase

Zero-point correction= 0.374590 (Hartree/Particle)
Thermal correction to Energy= 0.398602
Thermal correction to Enthalpy= 0.399546
Thermal correction to Gibbs Free Energy= 0.318208
Sum of electronic and zero-point Energies= -1225.989820
Sum of electronic and thermal Energies= -1225.965808
Sum of electronic and thermal Enthalpies= -1225.964864
Sum of electronic and thermal Free Energies= -1226.046202

**Name of cationic radical**	**Pinoresinol**

Cartesian Coordinates optimized at B3LYP/6-311G(d,p

1 2

O	1.17633300	-1.85698100	-1.11655600
O	-1.07432000	0.96883900	-0.89075200
O	5.91612000	1.73694600	0.03486600
O	-5.89354700	1.69418200	0.46476600
O	6.93263900	-0.46182800	1.05877200
O	-7.00727800	-0.68541500	0.62876300
C	0.55837600	-0.09827500	0.40778900
C	-0.59811500	-1.10698800	0.23852600
C	1.56982000	-0.54774500	-0.69849300
C	-1.54531800	-0.37719400	-0.77120000
C	0.10170200	-2.34499900	-0.30929400
C	-0.11226900	1.24031700	0.13208000
C	3.01009600	-0.52521400	-0.24885300
C	-3.00557300	-0.43292700	-0.38218000
C	3.75596600	0.65474900	-0.36103200
C	-3.72458700	0.73706400	-0.13516000
C	3.61207400	-1.68265500	0.28963900

C	-3.65623000	-1.68640300	-0.29893200
C	5.07199400	0.68653200	0.07873000
C	-5.06966200	0.66058700	0.20515900
C	4.92128400	-1.66479800	0.73136000
C	-4.99116900	-1.77703900	0.04236600
C	5.66673800	-0.48679400	0.63815600
C	-5.71624600	-0.60982900	0.30110600
C	5.47993100	2.97787600	-0.53848400
C	-5.39638300	3.03884400	0.37752100
H	1.00780500	-0.14578500	1.40107400
H	-1.10875400	-1.30784600	1.18206600
H	1.46079400	0.11249900	-1.56648600
H	-1.43737900	-0.83417400	-1.76263700
H	0.48664900	-2.95920500	0.51530400
H	-0.52266900	-2.97760200	-0.94305600
H	-0.60153300	1.61758800	1.03969700
H	0.55776200	2.01479800	-0.24533200
H	3.30967200	1.53432500	-0.80695500
H	-3.22244400	1.68805000	-0.23825700
H	3.04236800	-2.60107500	0.32671800
H	-3.10431500	-2.59455400	-0.51146800
H	5.39836600	-2.54575500	1.14151500
H	-5.50206000	-2.72889700	0.11032800
H	7.29869500	0.42372000	0.90755100
H	-7.35291700	0.21142600	0.76072100
H	4.64063500	3.38721600	0.02951300
H	5.20031600	2.83710300	-1.58542700
H	6.33267500	3.64813600	-0.47037800
H	-5.04436900	3.24935400	-0.63498700
H	-4.59242100	3.19519200	1.10068100
H	-6.24104100	3.67887900	0.61810800

Frequency and Energy at B3LYP/6-311G(d,p)in gas phase

Zero-point correction=	0.391947 (Hartree/Particle)
Thermal correction to Energy=	0.415384
Thermal correction to Enthalpy=	0.416328
Thermal correction to Gibbs Free Energy=	0.335406
Sum of electronic and zero-point Energies=	-1226.344197
Sum of electronic and thermal Energies=	-1226.320761
Sum of electronic and thermal Enthalpies=	-1226.319816
Sum of electronic and thermal Free Energies=	-1226.400738

**Name of compound (8)**	***α*-Conidendrin**

Cartesian Coordinates optimized at B3LYP/6-311G(d,p)

0 1

C	1.00734700	-1.62758900	0.47690900
C	2.13641800	-2.42134600	0.39978200
C	3.37819400	-1.82579300	0.11013500
C	3.45321600	-0.45705900	-0.08420900
C	-0.23168300	0.57695900	0.43204900
C	2.30883100	0.35517000	-0.00163300
C	1.06673500	-0.23940800	0.27634700
C	1.08709500	4.05971100	-0.00158900

C	-1.01596700	3.08895700	0.15653600
H	-1.88614400	3.12619100	-0.49663400
H	-1.34768400	3.06139000	1.20062300
C	1.27800000	2.59039100	0.33143600
C	-0.02357800	1.97158300	-0.16240400
H	1.29530000	2.53067500	1.43117100
H	0.03967300	1.87863500	-1.25403700
O	-0.25766600	4.30960200	-0.05672300
O	2.04966700	-3.76499200	0.60145500
H	2.93982100	-4.12645100	0.49721300
H	4.40634500	0.01021400	-0.30045900
C	2.48108700	1.85373700	-0.22438800
H	3.40437400	2.20377200	0.24473200
H	2.57840400	2.06975200	-1.29580400
H	-0.40139700	0.70519900	1.51056800
C	5.72206600	-2.21748700	-0.23661300
H	5.75017700	-1.74123400	-1.22238000
H	6.38345900	-3.08219700	-0.23332500
H	6.05391800	-1.50282500	0.52397700
O	4.42292100	-2.71474900	0.05710200
H	0.06143300	-2.11154800	0.68721100
C	-1.47189300	-0.10143500	-0.13603100
C	-1.53938400	-0.49565400	-1.47280500
C	-2.59445600	-0.30540300	0.68023600
C	-2.69568800	-1.07313300	-1.99427100
H	-0.67677800	-0.36905500	-2.11690200
C	-3.74979600	-0.88315600	0.16487600
C	-3.80651700	-1.27018300	-1.18544300
H	-2.75111800	-1.38413800	-3.03055800
O	-4.93494500	-1.83648200	-1.69201800
H	-5.57580900	-1.89112700	-0.97063800
H	-2.55189900	-0.01314900	1.72235900
C	-4.95804400	-0.80073100	2.24146500
H	-4.20062000	-1.34863200	2.81200200
H	-5.94941300	-1.09107300	2.58498700
H	-4.82260000	0.27558400	2.39267100
O	-4.90624800	-1.13719100	0.86138000
O	1.90860300	4.91021500	-0.18483000

Frequency and Energy at B3LYP/6-311G(d,p)in gas phase	
Zero-point correction= 0.369108 (Hartree/Particle)<br>Thermal correction to Energy= 0.392026<br>Thermal correction to Enthalpy= 0.392970<br>Thermal correction to Gibbs Free Energy= 0.316052<br>Sum of electronic and zero-point Energies= -1225.452369<br>Sum of electronic and thermal Energies= -1225.429452<br>Sum of electronic and thermal Enthalpies= -1225.428508<br>Sum of electronic and thermal Free Energies= -1225.505425	
Energy at ROB3LYP/6-311++G(2df,2p): in gas phase	HF=-1225.9233543
Energy at ROB3LYP/6-311++G(2df,2p): in H2O	HF=-1225.9431971
Energy at ROB3LYP/6-311++G(2df,2p): in ETHANOL	HF=-1225.9422574
**Name of radical**	***α*-Conidendrin-O4-H**
Cartesian Coordinates optimized at B3LYP/6-311G(d,p)	

0 2			
C	1.11807300	-1.51190900	0.35186700
C	2.30708000	-2.32984400	0.27555600
C	3.56093800	-1.60739300	0.03809400
C	3.53945300	-0.22085400	-0.08634300
C	-0.20835800	0.63074000	0.39801600
C	2.36692700	0.52399100	0.00099900
C	1.11438800	-0.14597200	0.22795200
C	0.96980400	4.16853900	0.03309600
C	-1.09328900	3.10769100	0.12884000
H	-1.94802900	3.11664900	-0.54498700
H	-1.44852100	3.05129000	1.16380100
C	1.21388300	2.70373200	0.35500900
C	-0.04686400	2.03754600	-0.18081400
H	1.20423600	2.63227400	1.45402100
H	0.04553300	1.96219000	-1.27186200
O	-0.38189500	4.36142800	-0.05048000
O	2.25601900	-3.57351700	0.40925100
H	4.48666400	0.27886800	-0.25834400
C	2.46333500	2.03076200	-0.17870400
H	3.36325200	2.41198300	0.31008600
H	2.57456200	2.26246400	-1.24603000
H	-0.36292000	0.74773200	1.47978900
C	4.96913100	-3.60564300	0.02582800
H	4.65931000	-3.98168400	0.99978800
H	6.04196100	-3.73078200	-0.11363600
H	4.41484500	-4.13329900	-0.74911500
O	4.76475600	-2.18295000	-0.06885700
H	0.19816100	-2.06049500	0.51483900
C	-1.43225500	-0.08721400	-0.15299500
C	-1.53932600	-0.41360700	-1.50528700
C	-2.49965600	-0.40359100	0.70048600
C	-2.68258900	-1.03387600	-2.00576900
H	-0.71962700	-0.19959000	-2.18162600
C	-3.64111500	-1.02562100	0.20635900
C	-3.73942400	-1.34263600	-1.16022000
H	-2.76885000	-1.29239600	-3.05420300
O	-4.85407500	-1.94925700	-1.64690500
H	-5.45514900	-2.08535200	-0.90230600
H	-2.42510900	-0.16430400	1.75417100
C	-4.73961100	-1.15330700	2.34058700
H	-3.92101400	-1.69245400	2.82880800
H	-5.69190400	-1.52815600	2.71138800
H	-4.65926100	-0.08400300	2.56347400
O	-4.74450400	-1.38766100	0.93810000
O	1.76078800	5.05194000	-0.12278400
Frequency and Energy at B3LYP/6-311G(d,p)in gas phase			
Zero-point correction=	0.356012 (Hartree/Particle)		
Thermal correction to Energy=	0.378785		
Thermal correction to Enthalpy=	0.379729		
Thermal correction to Gibbs Free Energy=	0.302109		
Sum of electronic and zero-point Energies=	-1224.824959		
Sum of electronic and thermal Energies=	-1224.802186		

Sum of electronic and thermal Enthalpies= -1224.801242<br>Sum of electronic and thermal Free Energies= -1224.878862		
Energy at ROB3LYP/6-311++G(2df,2p): in GAS PHASE		HF=-1225.2780464
Energy at ROB3LYP/6-311++G(2df,2p): in H2O		HF=-1225.2994159
Energy at ROB3LYP/6-311++G(2df,2p): in ETHANOL		HF=-1225.2983803
**Name of radical**	***α*-Conidendrin-O4'-H**	
Cartesian Coordinates optimized at B3LYP/6-311G(d,p)		

0 2

C	-0.98236700	-1.63092300	-0.58645400
C	-2.07333800	-2.47037500	-0.46011100
C	-3.31680600	-1.92996600	-0.08037300
C	-3.43237200	-0.56927500	0.14882500
C	0.17591300	0.61362200	-0.54550400
C	-2.32696900	0.28920000	0.01877200
C	-1.08320300	-0.25109900	-0.34548300
C	-1.25099200	4.03812100	-0.00274600
C	0.87727700	3.15544400	-0.28771500
H	1.78074600	3.22354500	0.31601800
H	1.15062100	3.14924100	-1.34859300
C	-1.40244600	2.56465400	-0.34055600
C	-0.05129400	1.99654600	0.07543800
H	-1.47992900	2.51285600	-1.43788000
H	-0.05294300	1.89438400	1.16807500
O	0.08377300	4.34106700	-0.01852300
O	-1.94778200	-3.80301700	-0.70036600
H	-2.81523400	-4.20352900	-0.55466200
H	-4.38854700	-0.14416200	0.42880200
C	-2.54176100	1.77638600	0.27558300
H	-3.50331500	2.09659100	-0.13375500
H	-2.58356900	1.97565800	1.35374800
H	0.31323300	0.75398100	-1.62592600
C	-5.61837100	-2.42195300	0.39760100
H	-5.60412700	-1.96765300	1.39381500
H	-6.24317100	-3.31312600	0.41468000
H	-6.02391200	-1.70676200	-0.32563000
O	-4.32016400	-2.85957900	0.01456900
H	-0.03583100	-2.07291400	-0.87396400
C	1.44457800	-0.02640300	-0.01002900
C	1.52023300	-0.45671300	1.34722000
C	2.56177500	-0.16032500	-0.82271100
C	2.67401600	-0.98170000	1.85040500
H	0.64062500	-0.37476800	1.97577100
C	3.76325500	-0.69703000	-0.34580800
C	3.87286900	-1.13746500	1.05053400
H	2.75205300	-1.31543000	2.87842000
O	4.92516100	-1.61757200	1.52699400
H	2.53221600	0.14774800	-1.86218600
C	6.05917300	-1.29688900	-0.94092400
H	6.54378400	-0.72127500	-0.15390000
H	6.60566300	-1.20853300	-1.87870900
H	5.99293400	-2.33788600	-0.62815600
O	4.75434500	-0.76418500	-1.24213000

O -2.09487200 4.85189700 0.23423600	
Frequency and Energy at B3LYP/6-311G(d,p)in gas phase	
Zero-point correction= 0.356078 (Hartree/Particle)<br>Thermal correction to Energy= 0.378855<br>Thermal correction to Enthalpy= 0.379799<br>Thermal correction to Gibbs Free Energy= 0.302290<br>Sum of electronic and zero-point Energies= -1224.825828<br>Sum of electronic and thermal Energies= -1224.803051<br>Sum of electronic and thermal Enthalpies= -1224.802106<br>Sum of electronic and thermal Free Energies= -1224.879616	
Energy at ROB3LYP/6-311++G(2df,2p): in GAS PHASE	HF=-1225.2790513
Energy at ROB3LYP/6-311++G(2df,2p): in H2O	HF=-1225.3000299
Energy at ROB3LYP/6-311++G(2df,2p): in ETHANOL	HF=-1225.2990429
**Name of radical**	***α*-Conidendrin-C7'-H**
Cartesian Coordinates optimized at B3LYP/6-311G(d,p	
0 2<br>C -0.80241400 -1.56799600 0.50335400<br>C -1.87299300 -2.43067400 0.44523200<br>C -3.16306200 -1.92816200 0.17417500<br>C -3.33742500 -0.56430100 -0.01854800<br>C 0.19669100 0.71686600 0.37888500<br>C -2.26182900 0.32484100 0.03708600<br>C -0.94188300 -0.16818700 0.28359500<br>C -1.22256700 4.03008000 -0.56485800<br>C 0.87367900 3.27284600 0.08118900<br>H 1.54665300 3.58658900 0.87553000<br>H 1.46017600 2.99640800 -0.79980400<br>C -1.25989100 2.51563100 -0.52147900<br>C -0.13566000 2.18407400 0.46629300<br>H -0.93495900 2.17851300 -1.51774700<br>H -0.50953500 2.42629800 1.47532600<br>O 0.04538100 4.42350600 -0.24301000<br>O -1.69284600 -3.76420400 0.66211100<br>H -2.56211500 -4.18249900 0.60746600<br>H -4.32532900 -0.16783000 -0.21996100<br>C -2.54544000 1.80575800 -0.15988200<br>H -3.30537000 1.95333100 -0.93189000<br>H -2.95350900 2.23738200 0.76344500<br>C -5.48673000 -2.47949000 -0.08089200<br>H -5.82691600 -1.79633700 0.70422200<br>H -6.08421700 -3.38898200 -0.05488700<br>H -5.59775400 -1.99871200 -1.05830700<br>O -4.14149800 -2.88667000 0.14091700<br>H 0.15985300 -1.98622600 0.76270900<br>C 1.58369900 0.26692600 0.43024200<br>C 2.51339200 0.87348600 1.29585400<br>C 2.05835900 -0.76981700 -0.41252200<br>C 3.84362600 0.46709000 1.33882400<br>H 2.18442600 1.64642100 1.97930700<br>C 3.38245000 -1.16928400 -0.37242300<br>C 4.29256000 -0.55200000 0.51052700<br>H 4.54866300 0.92672000 2.02097400	

O	5.59052200	-0.94947100	0.54572000
H	5.68891100	-1.66058600	-0.10207500
H	1.37703700	-1.22651400	-1.11622100
C	3.12623600	-2.86439400	-2.05756400
H	2.31660300	-3.38087200	-1.53211600
H	3.77316800	-3.59635200	-2.53787600
H	2.70389700	-2.19860800	-2.81737300
O	3.95356900	-2.14679500	-1.14879100
O	-2.09318600	4.80514600	-0.83735300

Frequency and Energy at B3LYP/6-311G(d,p)in gas phase (HF = -1225.2824055)

Zero-point correction= 0.356018 (Hartree/Particle)
Thermal correction to Energy= 0.378907
Thermal correction to Enthalpy= 0.379851
Thermal correction to Gibbs Free Energy= 0.302854
Sum of electronic and zero-point Energies= -1224.826906
Sum of electronic and thermal Energies= -1224.804017
Sum of electronic and thermal Enthalpies= -1224.803073
Sum of electronic and thermal Free Energies= -1224.880070

**Name of anion**	***α*-Conidendrin-O4-H**

Cartesian Coordinates optimized at B3LYP/6-311G(d,p

-1 1

C	1.25137300	-1.53383700	0.36506300
C	2.44762400	-2.31559100	0.21719900
C	3.61067100	-1.50944400	-0.12067800
C	3.52714200	-0.14311600	-0.29420000
C	-0.18825800	0.53743000	0.41134800
C	2.32531900	0.57920500	-0.16761900
C	1.16802500	-0.15474000	0.16937500
C	0.76259800	4.14612200	0.01968200
C	-1.22205300	2.96617000	0.26096000
H	-2.13255600	2.93572300	-0.33681800
H	-1.48359700	2.87261300	1.32183400
C	1.12001000	2.69432700	0.27163600
C	-0.14840600	1.96257600	-0.15297000
H	1.22572100	2.59418800	1.36447900
H	-0.14553100	1.90162600	-1.24911700
O	-0.60589100	4.26655000	0.05236500
O	2.48393300	-3.57143100	0.36469000
H	4.44562100	0.38116200	-0.55084700
C	2.34028300	2.08397200	-0.39352900
H	3.25800900	2.52711100	0.00695100
H	2.32127900	2.34143200	-1.46471700
H	-0.32892900	0.62691800	1.49952800
C	5.31023800	-2.96526000	0.68411000
H	5.50678300	-2.40254300	1.61014700
H	6.25178500	-3.39275800	0.32708400
H	4.57405800	-3.74749400	0.87690600
O	4.84935300	-2.09575400	-0.34715200
H	0.36005800	-2.10251700	0.61389500
C	-1.39835100	-0.21542500	-0.12747100
C	-1.45059100	-0.66506100	-1.44836900
C	-2.51712900	-0.42717900	0.68983500

C	-2.58851700	-1.29246500	-1.95171600
H	-0.57791600	-0.55177700	-2.07997300
C	-3.65155800	-1.06129300	0.19352400
C	-3.69621600	-1.49351900	-1.13908200
H	-2.62592700	-1.65219300	-2.97347400
O	-4.81479800	-2.11691400	-1.62490100
H	-5.43112200	-2.18489000	-0.88438400
H	-2.48048200	-0.10412900	1.72323400
C	-4.81282600	-1.04284900	2.29587600
H	-4.01559200	-1.58101400	2.82037000
H	-5.78134100	-1.37436800	2.66992800
H	-4.70370800	0.03195200	2.48182100
O	-4.80491100	-1.33210100	0.90833900
O	1.47070400	5.09725200	-0.16267800

Frequency and Energy at B3LYP/6-311G(d,p)in gas phase

Zero-point correction= 0.354251 (Hartree/Particle)
Thermal correction to Energy= 0.377008
Thermal correction to Enthalpy= 0.377953
Thermal correction to Gibbs Free Energy= 0.300990
Sum of electronic and zero-point Energies= -1224.902375
Sum of electronic and thermal Energies= -1224.879617
Sum of electronic and thermal Enthalpies= -1224.878673
Sum of electronic and thermal Free Energies= -1224.955636

**Name of anion**	***α*-Conidendrin-O4'-H**

Cartesian Coordinates optimized at B3LYP/6-311G(d,p

-1 1

C	0.82672100	-1.59233300	0.15078500
C	1.82773400	-2.53623200	-0.03145700
C	3.15226200	-2.10868200	-0.21263500
C	3.41333600	-0.74537800	-0.23080500
C	-0.09482100	0.74822000	0.39819600
C	2.40815600	0.21556700	-0.06993700
C	1.08420400	-0.21811700	0.14139400
C	1.67585800	4.04305100	0.16450600
C	-0.53871300	3.33926100	0.23692100
H	-1.37850900	3.52375300	-0.42900700
H	-0.89817900	3.29758500	1.27011000
C	1.67088400	2.54633200	0.41133500
C	0.30799800	2.12558300	-0.12997800
H	1.65073500	2.42515200	1.50660000
H	0.37239000	2.07595300	-1.22472100
O	0.38165800	4.46948700	0.11259900
O	1.56760300	-3.88575800	-0.05294100
H	0.61341400	-4.00250900	0.01102200
H	4.44221500	-0.43949600	-0.39355200
C	2.78776700	1.69096900	-0.15865000
H	3.73095500	1.87549400	0.36404900
H	2.95856500	1.96734500	-1.20688200
H	-0.17598800	0.84361700	1.49195200
C	4.48697700	-3.88121700	0.63279800
H	4.75235700	-3.32374900	1.53993000

H	5.34709300	-4.46960700	0.30963100
H	3.64790200	-4.54723800	0.84654200
O	4.18806600	-2.99044500	-0.44172600
H	-0.20355400	-1.91685400	0.27147700
C	-1.44381800	0.26608800	-0.09479900
C	-1.71916900	0.04195200	-1.45352000
C	-2.48457300	-0.00215100	0.81429200
C	-2.95938500	-0.40880900	-1.87664300
H	-0.93939800	0.21114900	-2.19487000
C	-3.73294400	-0.44279100	0.41039800
C	-4.06667100	-0.66555400	-0.98781400
H	-3.16012200	-0.56309900	-2.93348800
O	-5.20545600	-1.04231500	-1.38491900
H	-2.33229700	0.14661100	1.88206800
C	-5.36451900	-1.85037100	1.41242900
H	-5.83110600	-2.03608900	0.44332300
H	-6.11809600	-1.79275400	2.20326800
H	-4.66465100	-2.66713700	1.65089200
O	-4.69254000	-0.59421900	1.40579800
O	2.60599100	4.79319200	0.04003400

Frequency and Energy at B3LYP/6-311G(d,p)in gas phase

Zero-point correction= 0.353796 (Hartree/Particle)
Thermal correction to Energy= 0.376733
Thermal correction to Enthalpy= 0.377677
Thermal correction to Gibbs Free Energy= 0.300492
Sum of electronic and zero-point Energies= -1224.901661
Sum of electronic and thermal Energies= -1224.878724
Sum of electronic and thermal Enthalpies= -1224.877780
Sum of electronic and thermal Free Energies= -1224.954965

Name of anion	Cyclolariciresinol-O4’-H

Cartesian Coordinates optimized at B3LYP/6-311G(d,p

-1 1

C	-1.42778600	-1.55497500	-0.52175400
C	-2.70906200	-2.06309600	-0.56729400
C	-3.80539600	-1.27667500	-0.18310400
C	-3.56918300	0.02927700	0.22818700
C	0.22307300	0.27738800	-0.02424200
C	-2.27809700	0.55081600	0.30701400
C	-1.12668000	-0.23307100	-0.06175500
C	-0.47939400	3.97102500	0.09617600
C	1.36210300	2.68496300	-0.45125100
H	2.37733000	2.66986600	-0.05948000
H	1.37211300	2.50672300	-1.53297600
C	-0.91217600	2.53265300	-0.03809200
C	0.36910800	1.75623800	0.27353000
H	-1.11998200	2.38730700	-1.11162500
H	0.58610800	1.92125100	1.35048700
O	0.85267900	4.03372000	-0.20436000
O	-2.92715100	-3.34794500	-1.02002600
H	-3.88191700	-3.48149400	-0.97213700
H	-4.39853400	0.67178700	0.50709300
C	-2.09571400	1.99491300	0.73841800

H	-2.99873500	2.58173400	0.54107400
H	-1.88917700	2.08012600	1.81488500
C	-6.15889700	-1.24552700	0.26996200
H	-5.99975000	-1.03303000	1.33606100
H	-7.01459000	-1.91432200	0.15861000
H	-6.37976000	-0.29948600	-0.24349700
O	-5.05312000	-1.90502900	-0.29855000
H	-0.63312500	-2.19660600	-0.87868700
C	1.38525400	-0.58112000	0.13968600
C	1.33656900	-1.85829200	0.76584400
C	2.69285700	-0.17211000	-0.26474400
C	2.46464000	-2.64478100	0.96460800
H	0.38732800	-2.22301900	1.13465400
C	3.81704600	-0.95861000	-0.04691600
C	3.72381800	-2.21139600	0.56647000
H	2.38426900	-3.60746300	1.46009700
O	4.85036000	-2.98923800	0.77109500
H	5.58536500	-2.47732600	0.41246600
H	2.80955600	0.74933700	-0.81348600
C	5.32571900	0.63213800	-1.04242700
H	4.78991400	0.70665700	-1.99669300
H	6.39842200	0.70646900	-1.22784800
H	5.01238300	1.46104300	-0.39649800
O	5.11595000	-0.61862800	-0.42126300
O	-1.11547000	4.95361000	0.37655700

Frequency and Energy at B3LYP/6-311G(d,p)in gas phase

Zero-point correction= 0.353139 (Hartree/Particle)
Thermal correction to Energy= 0.376381
Thermal correction to Enthalpy= 0.377325
Thermal correction to Gibbs Free Energy= 0.299284
Sum of electronic and zero-point Energies= -1224.868255
Sum of electronic and thermal Energies= -1224.845013
Sum of electronic and thermal Enthalpies= -1224.844069
Sum of electronic and thermal Free Energies= -1224.922110

**Name of cationic radical**	***α*-Conidendrin**

Cartesian Coordinates optimized at B3LYP/6-311G(d,p

1 2

C	-0.92976000	-1.62502900	-0.51548700
C	-2.03943800	-2.44885800	-0.42917400
C	-3.31047700	-1.88281800	-0.09972700
C	-3.41747000	-0.51191000	0.11811800
C	0.23452700	0.61238500	-0.47168100
C	-2.30091300	0.31786600	0.03702800
C	-1.02463800	-0.25014900	-0.29325100
C	-1.18781900	4.05264000	-0.00218300
C	0.94039500	3.16332800	-0.25848700
H	1.83868900	3.23884800	0.35173500
H	1.22054900	3.13919600	-1.31747700
C	-1.33808500	2.57238900	-0.33209500
C	0.00349900	2.01233700	0.11810300
H	-1.40268300	2.51028600	-1.42892500
H	-0.01358900	1.93687800	1.21234200

O	0.14741300	4.34828700	-0.01077300
O	-1.92378600	-3.76073300	-0.66278300
H	-2.79371900	-4.17895500	-0.57422800
H	-4.37910400	-0.07477800	0.35399800
C	-2.48739500	1.80328800	0.28584400
H	-3.44963600	2.13267100	-0.11286300
H	-2.52360900	1.99515900	1.36604300
H	0.38648700	0.73341100	-1.55240800
C	-5.64810400	-2.36393100	0.24583900
H	-5.69326300	-1.91648300	1.24079600
H	-6.24504900	-3.27114600	0.21820500
H	-6.00128600	-1.66009300	-0.51062100
O	-4.30135500	-2.78190400	-0.05216700
H	0.01801800	-2.08470600	-0.76575200
C	1.47189900	-0.04525100	0.10318000
C	1.51228800	-0.41510000	1.46790800
C	2.59337300	-0.27623000	-0.70094400
C	2.63831700	-0.99235100	2.02217800
H	0.64578200	-0.24253800	2.09465600
C	3.73538100	-0.85569700	-0.15745500
C	3.76142200	-1.22259500	1.22350400
H	2.68276000	-1.27077500	3.06731100
O	4.85295100	-1.77443800	1.75227900
H	5.53538800	-1.85452800	1.06725800
H	2.57000300	-0.00113300	-1.74744900
C	5.02411900	-0.80938700	-2.19369500
H	4.29296800	-1.36479700	-2.78587500
H	6.03017200	-1.11760200	-2.46470800
H	4.90954100	0.26562900	-2.35142300
O	4.88563100	-1.12812100	-0.79896900
O	-2.03774900	4.85639500	0.22669500

Frequency and Energy at B3LYP/6-311G(d,p)in gas phase

Zero-point correction= 0.369154 (Hartree/Particle)
Thermal correction to Energy= 0.392080
Thermal correction to Enthalpy= 0.393024
Thermal correction to Gibbs Free Energy= 0.315713
Sum of electronic and zero-point Energies= -1225.194310
Sum of electronic and thermal Energies= -1225.171385
Sum of electronic and thermal Enthalpies= -1225.170441
Sum of electronic and thermal Free Energies= -1225.247751

**Name of compound (9)**	**Lariciresinol**

Cartesian Coordinates optimized at B3LYP/6-311G(d,p)

0 1

O	0.39574800	-1.29417000	0.07943600
O	0.53955500	3.46464700	0.48010000
O	6.02384900	0.25049000	-1.41342700
O	-5.62114400	-1.69210500	-1.23294000
O	6.75331300	-1.50073900	0.62108700
O	-7.16569600	-0.00157700	0.34447400
C	0.77650500	1.01977300	0.46671000
C	-0.72475500	0.70909500	0.68367400

C	1.27630200	-0.22976200	-0.30586500	
C	-0.64352500	-0.79780800	0.93733400	
C	-1.63015600	1.08340900	-0.51356500	
C	1.11255300	2.34917800	-0.20184900	
C	2.72375900	-0.59597300	-0.04792600	
C	-3.09738400	0.79906100	-0.27402300	
C	3.74262300	-0.01627400	-0.80914700	
C	3.07652100	-1.49164400	0.96007000	
C	-3.71979200	-0.32958900	-0.81626700	
C	-3.87245500	1.65798800	0.50900400	
C	5.08540800	-0.30146200	-0.57947800	
C	4.41551000	-1.78957900	1.19997100	
C	5.42758400	-1.20795100	0.43918400	
C	-5.06864400	-0.60545700	-0.60476100	
C	-5.22145100	1.39675600	0.73364300	
C	-5.83403800	0.27518200	0.17799300	
C	6.99077200	1.11355000	-0.80467700	
C	-6.15159900	-2.71737100	-0.38559600	
H	1.25917700	1.00764100	1.45378600	
H	-1.09431100	1.23233100	1.56914300	
H	1.15209400	-0.06209800	-1.38523900	
H	-0.38805500	-1.00237200	1.98625300	
H	-1.56328000	-1.33328700	0.69772200	
H	-1.29942500	0.54877600	-1.40934100	
H	-1.50777000	2.15113900	-0.71155600	
H	0.70796200	2.38722100	-1.21539600	
H	2.20147200	2.45997600	-0.28231700	
H	3.51565300	0.65931600	-1.62701200	
H	2.29949600	-1.98091400	1.53286800	
H	0.92316600	3.49932600	1.36211300	
H	-3.16700700	-1.02020200	-1.44381800	
H	-3.42595400	2.54951400	0.93675500	
H	4.68121600	-2.50281400	1.97604700	
H	-5.81535700	2.08298100	1.33181800	
H	6.83245000	-2.16060100	1.31752700	
H	-7.56378600	0.69628500	0.87466000	
H	6.49671100	1.94800900	-0.29343700	
H	7.59977000	1.50202400	-1.62044900	
H	7.62355100	0.57161400	-0.09964500	
H	-6.99599400	-2.35319600	0.20247700	
H	-5.37474000	-3.10691500	0.28234600	
H	-6.48446400	-3.51284100	-1.05172500	
Frequency and Energy at B3LYP/6-311G(d,p)in gas phase				
Zero-point correction= 0.411364 (Hartree/Particle)<br>Thermal correction to Energy= 0.437198<br>Thermal correction to Enthalpy= 0.438143<br>Thermal correction to Gibbs Free Energy= 0.352014<br>Sum of electronic and zero-point Energies= -1227.773787<br>Sum of electronic and thermal Energies= -1227.747952<br>Sum of electronic and thermal Enthalpies= -1227.747008<br>Sum of electronic and thermal Free Energies= -1227.833137				
Energy at ROB3LYP/6-311++G(2df,2p): in gas phase				HF=-1228.2879624

Energy at ROB3LYP/6-311++G(2df,2p): in H2O		HF=-1228.3092214
Energy at ROB3LYP/6-311++G(2df,2p): in ETHANOL		HF=-1228.3081139
**Name of radical**	**Lariciresinol-O4-H**	
Cartesian Coordinates optimized at B3LYP/6-311G(d,p)		

0 2

O	0.42967200	-1.22321400	0.09390600
O	0.51495900	3.54499500	0.37381000
O	5.98583300	0.31332100	-1.35432000
O	-5.54232000	-1.76645000	-1.23764000
O	6.67196300	-1.30204100	0.95782900
O	-7.13072400	-0.10264000	0.32637500
C	0.78563800	1.10657800	0.44392800
C	-0.71087700	0.77486900	0.66898700
C	1.30555000	-0.15998500	-0.29310000
C	-0.60810500	-0.72429700	0.95471100
C	-1.62305900	1.11048600	-0.53441500
C	1.09985300	2.41687300	-0.27301300
C	2.74639700	-0.50011000	0.01008400
C	-3.08314400	0.79239500	-0.29284000
C	3.76452700	0.00844300	-0.78695700
C	3.07283100	-1.31677200	1.12734900
C	-3.67672600	-0.35523300	-0.82740400
C	-3.87955400	1.63777800	0.48328500
C	5.10995000	-0.23967000	-0.50502700
C	4.37629000	-1.58035300	1.43498100
C	5.48186800	-1.05975700	0.65634100
C	-5.01871200	-0.66275500	-0.61565800
C	-5.22188900	1.34485600	0.70847900
C	-5.80635200	0.20478400	0.16011300
C	7.41126500	0.13641800	-1.24752200
C	-6.05795900	-2.79683400	-0.38671600
H	1.26609000	1.13753200	1.43125800
H	-1.08583400	1.31087100	1.54425400
H	1.20682200	-0.01630300	-1.37793000
H	-0.33832100	-0.90423700	2.00434000
H	-1.52087100	-1.27868300	0.73443000
H	-1.27834600	0.57220900	-1.42282500
H	-1.52599300	2.17809400	-0.74636900
H	0.68673800	2.41171500	-1.28372400
H	2.18660300	2.53916300	-0.36638400
H	3.54015700	0.60660900	-1.66362900
H	2.26671400	-1.74356900	1.71047400
H	0.92494200	3.63687600	1.23973900
H	-3.10651600	-1.03680100	-1.44928800
H	-3.45641900	2.54353200	0.90481300
H	4.65247200	-2.20204000	2.27867300
H	-5.83306500	2.02059000	1.30106500
H	-7.54706800	0.58817200	0.85182600
H	7.78730300	0.54058000	-0.30891600
H	7.81258700	0.68956800	-2.09562500
H	7.68093600	-0.91686700	-1.31148800
H	-6.91650100	-2.44744500	0.18945600

H -5.27914200 -3.16253500 0.29222600<br>H -6.36510400 -3.60507700 -1.04969100	
Frequency and Energy at B3LYP/6-311G(d,p)in gas phase	
Zero-point correction= 0.399124 (Hartree/Particle)<br>Thermal correction to Energy= 0.424513<br>Thermal correction to Enthalpy= 0.425457<br>Thermal correction to Gibbs Free Energy= 0.339791<br>Sum of electronic and zero-point Energies= -1227.155547<br>Sum of electronic and thermal Energies= -1227.130158<br>Sum of electronic and thermal Enthalpies= -1227.129214<br>Sum of electronic and thermal Free Energies= -1227.214880	
Energy at ROB3LYP/6-311++G(2df,2p): in GAS PHASE	HF=-1227.653005
Energy at ROB3LYP/6-311++G(2df,2p): in H2O	HF=-1227.6735548
Energy at ROB3LYP/6-311++G(2df,2p): in ETHANOL	HF=-1227.6725211
**Name of radical**	**Lariciresinol-O4'-H**
Cartesian Coordinates optimized at B3LYP/6-311G(d,p)	
0 2<br>O 0.41202200 -1.34639900 0.05005600<br>O 0.44854200 3.41571500 0.30996100<br>O 6.04168000 0.29548600 -1.37194600<br>O -5.62206800 -1.76385200 -1.02335900<br>O 6.77017300 -1.46582100 0.65488700<br>O -7.07954400 0.07735200 0.68312700<br>C 0.76863300 0.98192400 0.39080700<br>C -0.72128200 0.64618100 0.64578600<br>C 1.28722000 -0.28154700 -0.34857700<br>C -0.60513800 -0.85230500 0.93258400<br>C -1.65492600 0.96457800 -0.54890800<br>C 1.05736400 2.29516400 -0.33052700<br>C 2.73684700 -0.62495700 -0.06918700<br>C -3.10921400 0.70759000 -0.24545600<br>C 3.75735800 -0.01877700 -0.80751500<br>C 3.08894000 -1.52529500 0.93451400<br>C -3.77064200 -0.40464500 -0.75089400<br>C -3.82634900 1.61903000 0.58347900<br>C 5.10107900 -0.28257400 -0.55918900<br>C 4.42922600 -1.80212700 1.19284300<br>C 5.44292800 -1.19448500 0.45521600<br>C -5.11606500 -0.65689000 -0.46455400<br>C -5.13953600 1.40741100 0.88770200<br>C -5.87541800 0.26098300 0.39403100<br>C 6.98854700 1.16380300 -0.73881900<br>C -6.98875700 -2.18348400 -0.85081300<br>H 1.26441800 1.01670800 1.37019600<br>H -1.08402500 1.18588800 1.52366500<br>H 1.17031700 -0.14200600 -1.43250200<br>H -0.31264000 -1.02514300 1.97755100<br>H -1.52070700 -1.41230800 0.73634200<br>H -1.35181300 0.37880700 -1.42125900<br>H -1.53667500 2.02168100 -0.80147900<br>H 0.64680300 2.27822400 -1.34241900<br>H 2.14101300 2.43958600 -0.42271700	

H 3.53262900 0.66144500 -1.62216200
H 2.31215400 -2.03501700 1.48939700
H 0.84900400 3.51407100 1.17963100
H -3.25647700 -1.10958600 -1.39474700
H -3.31265400 2.49680200 0.96233100
H 4.69472400 -2.51925200 1.96529500
H -5.70352200 2.09190500 1.51046300
H 6.85097100 -2.13181000 1.34533900
H 6.47577100 1.98221300 -0.22020800
H 7.59996900 1.57438900 -1.54175400
H 7.62184300 0.62066400 -0.03536100
H -7.68010600 -1.43055800 -1.22673300
H -7.21071200 -2.37358600 0.19822600
H -7.06161000 -3.10044800 -1.43405900

Frequency and Energy at B3LYP/6-311G(d,p)in gas phase

Zero-point correction= 0.399180 (Hartree/Particle)
Thermal correction to Energy= 0.424580
Thermal correction to Enthalpy= 0.425524
Thermal correction to Gibbs Free Energy= 0.339424
Sum of electronic and zero-point Energies= -1227.155777
Sum of electronic and thermal Energies= -1227.130377
Sum of electronic and thermal Enthalpies= -1227.129432
Sum of electronic and thermal Free Energies= -1227.215533

Energy at ROB3LYP/6-311++G(2df,2p): in GAS PHASE	HF=-1227.6531929
Energy at ROB3LYP/6-311++G(2df,2p): in H2O	HF=-1227.6738182
Energy at ROB3LYP/6-311++G(2df,2p): in ETHANOL	HF=-1227.6727878
**Name of radical**	**Lariciresinol-11**

Cartesian Coordinates optimized at B3LYP/6-311G(d,p)

0 2
O 0.39087200 -1.29022100 0.14456800
O 0.69186100 3.45718200 0.52751200
O 5.98481500 0.24194400 -1.46777700
O -5.61747500 -1.64016600 -1.28415000
O 6.76276000 -1.44671200 0.60529200
O -7.16646400 0.01781800 0.32386500
C 0.77876500 1.01783000 0.52435000
C -0.72765200 0.72496300 0.72726300
C 1.26571300 -0.22809600 -0.25603200
C -0.66094100 -0.78343800 0.98310000
C -1.62308300 1.10190200 -0.47761900
C 1.13118300 2.35096800 -0.13991600
C 2.71733500 -0.58591500 -0.01732300
C -3.09304400 0.81916100 -0.25104700
C 3.71833400 -0.02340300 -0.81447600
C 3.09346500 -1.44694500 1.01223600
C -3.71537400 -0.29410600 -0.82395700
C -3.86917300 1.66115900 0.54889500
C 5.06768700 -0.29183300 -0.60018700
C 4.43810100 -1.72966900 1.23534900
C 5.43304500 -1.16548800 0.43881400
C -5.06629500 -0.57072400 -0.62705000
C -5.22026100 1.39909000 0.75893300

C	-5.83341200	0.29371200	0.17221400
C	6.97021500	1.11512500	-0.90317100
C	-6.16116900	-2.68236400	-0.46568900
H	1.25269200	1.00967500	1.51177600
H	-1.09440000	1.25305600	1.61107600
H	1.12764600	-0.06107000	-1.33438000
H	-0.42467900	-0.98831100	2.03570600
H	-1.58100700	-1.31101500	0.72775600
H	-1.28629700	0.56764100	-1.37133400
H	-1.50183700	2.16956700	-0.68405000
H	0.84348200	2.38696600	-1.20566800
H	2.23755500	2.46337400	-0.15619400
H	3.47222000	0.62170400	-1.65157900
H	2.33044100	-1.92067800	1.61614000
H	-3.16094300	-0.97209200	-1.46368300
H	-3.42351100	2.53953800	1.00389400
H	4.72279200	-2.41681700	2.02795700
H	-5.81491000	2.07206900	1.37108400
H	6.85914600	-2.08000900	1.32393000
H	-7.56581100	0.70267900	0.86991300
H	6.49165800	1.95785500	-0.39102500
H	7.55313400	1.48952800	-1.74407900
H	7.62411900	0.58481800	-0.20906600
H	-7.00919300	-2.32746100	0.12260400
H	-5.39250100	-3.09115400	0.20011800
H	-6.49137100	-3.46020800	-1.15349300

Frequency and Energy at B3LYP/6-311G(d,p)in gas phase

Zero-point correction= 0.396503 (Hartree/Particle)
Thermal correction to Energy= 0.422077
Thermal correction to Enthalpy= 0.423021
Thermal correction to Gibbs Free Energy= 0.336994
Sum of electronic and zero-point Energies= -1227.116635
Sum of electronic and thermal Energies= -1227.091062
Sum of electronic and thermal Enthalpies= -1227.090118
Sum of electronic and thermal Free Energies= -1227.176144

Energy at ROB3LYP/6-311++G(2df,2p): in GAS PHASE	HF=-1227.6128588
**Name of anion**	**Lariciresinol-O4-H**

Cartesian Coordinates optimized at B3LYP/6-311G(d,p

-1 1

O	0.42617700	-1.07522200	0.25543600
O	0.43170600	3.69747300	-0.18756400
O	5.93398000	-1.10760600	-1.51777900
O	-5.47681500	-2.08015300	-0.90287300
O	6.77683700	-0.95353300	1.22239900
O	-7.13680900	-0.22318900	0.30887100
C	0.72146700	1.29557200	0.28289400
C	-0.76091200	0.94391600	0.54436900
C	1.35678700	-0.03180200	-0.21399200
C	-0.58166400	-0.48877500	1.06313100
C	-1.65742000	1.02690900	-0.71310800
C	1.01217500	2.46618900	-0.64459600
C	2.78097000	-0.25795000	0.18654200

C	-3.11083600	0.70507200	-0.44545300
C	3.75777400	-0.54701200	-0.78556500
C	3.21838600	-0.20355300	1.52151000
C	-3.65564600	-0.54418700	-0.76130700
C	-3.95495800	1.64816400	0.14765200
C	5.08458000	-0.76812600	-0.46769900
C	4.54231500	-0.42026600	1.86063800
C	5.57469800	-0.73210200	0.90111400
C	-4.98895400	-0.85537800	-0.50785000
C	-5.28981600	1.35183900	0.41238400
C	-5.82021700	0.10578300	0.08632700
C	7.12080700	-0.32646300	-1.60802900
C	-5.84487500	-2.96615600	0.15679600
H	1.17631000	1.52870600	1.25419200
H	-1.18658900	1.59485600	1.31512400
H	1.30324600	-0.07177100	-1.31010300
H	-0.27777000	-0.46842600	2.12228800
H	-1.48530900	-1.09973000	0.98470500
H	-1.26809800	0.34867500	-1.47773000
H	-1.59217400	2.04189100	-1.11462100
H	0.59012500	2.29811000	-1.63798700
H	2.09777200	2.56573700	-0.76340800
H	3.47848800	-0.61649300	-1.83534400
H	2.49974400	-0.00469300	2.31560300
H	0.80247100	3.87024000	0.68366200
H	-3.04475400	-1.30825000	-1.22944000
H	-3.56806100	2.63005200	0.39936700
H	4.86101900	-0.39171600	2.89951900
H	-5.93456900	2.10111900	0.86597900
H	-7.57046900	0.53299700	0.71637200
H	6.88331700	0.72404700	-1.84009700
H	7.70383100	-0.74444400	-2.43439800
H	7.68169500	-0.37900100	-0.67290800
H	-6.66987400	-2.55998600	0.74766100
H	-4.98799900	-3.16980100	0.80931700
H	-6.16092900	-3.89433000	-0.32022500

Frequency and Energy at B3LYP/6-311G(d,p)in gas phase

Zero-point correction=	0.397389 (Hartree/Particle)
Thermal correction to Energy=	0.422735
Thermal correction to Enthalpy=	0.423680
Thermal correction to Gibbs Free Energy=	0.338640
Sum of electronic and zero-point Energies=	-1227.228521
Sum of electronic and thermal Energies=	-1227.203174
Sum of electronic and thermal Enthalpies=	-1227.202230
Sum of electronic and thermal Free Energies=	-1227.287270

**Name of anion**	**Lariciresinol-O4’-**

Cartesian Coordinates optimized at B3LYP/6-311G(d,p

-1 1

O	0.36790800	-1.28258600	0.23565200
O	0.69079300	3.50132400	0.47719100
O	5.96223200	0.14355300	-1.48602400

O	-5.59432700	-1.71855700	-1.27324500
O	6.72125300	-1.50379300	0.61470800
O	-7.18429200	-0.00902200	0.39541400
C	0.75185200	1.04158000	0.48356200
C	-0.76087400	0.77517000	0.68794200
C	1.22080800	-0.24574100	-0.24243700
C	-0.73522400	-0.72478100	0.98988000
C	-1.67109900	1.13779200	-0.51475400
C	1.14626700	2.33741000	-0.21438800
C	2.67556800	-0.60741100	-0.01267100
C	-3.13408800	0.83533300	-0.28176300
C	3.68117400	-0.07728000	-0.82653900
C	3.04913100	-1.45693400	1.02849300
C	-3.75202100	-0.30633600	-0.82482700
C	-3.93821300	1.66550200	0.51230600
C	5.02636700	-0.36249900	-0.61214600
C	4.39107900	-1.75350400	1.25265800
C	5.38800500	-1.21602700	0.44169200
C	-5.08701800	-0.60527800	-0.60706700
C	-5.27379100	1.37591700	0.75378600
C	-5.95584600	0.23247900	0.20336000
C	6.88311800	1.08306200	-0.92842200
C	-6.28214500	-2.65041500	-0.44528400
H	1.23366000	1.04017100	1.47431200
H	-1.12876500	1.32738700	1.55645400
H	1.06282400	-0.12481600	-1.32495800
H	-0.56145900	-0.91861400	2.05683600
H	-1.65356600	-1.22690500	0.68571000
H	-1.32489800	0.60283800	-1.40821600
H	-1.54000900	2.20689700	-0.71696700
H	0.70147100	2.39331100	-1.20977600
H	2.23861200	2.37323700	-0.33535300
H	3.43737100	0.56031300	-1.66949500
H	2.27764900	-1.90610900	1.64009700
H	0.90758200	3.38674800	1.40769400
H	-3.19024700	-0.98401000	-1.46551100
H	-3.50622600	2.57012500	0.94156500
H	4.67082200	-2.42819000	2.05857900
H	-5.88567100	2.04455900	1.35427600
H	6.80258200	-2.11970800	1.34967500
H	6.35252900	1.94464600	-0.50579500
H	7.51107800	1.41859100	-1.75434900
H	7.50706000	0.62224400	-0.15886200
H	-7.09052200	-2.15222200	0.09375400
H	-5.59145700	-3.12237900	0.27191900
H	-6.67725700	-3.42372200	-1.11144800

Frequency and Energy at B3LYP/6-311G(d,p)in gas phase

Zero-point correction=	0.397278 (Hartree/Particle)
Thermal correction to Energy=	0.422625
Thermal correction to Enthalpy=	0.423570
Thermal correction to Gibbs Free Energy=	0.339241
Sum of electronic and zero-point Energies=	-1227.225053
Sum of electronic and thermal Energies=	-1227.199706

Sum of electronic and thermal Enthalpies= -1227.198761<br>Sum of electronic and thermal Free Energies= -1227.283090			
**Name of cationic radical**	**Lariciresinol**		
Cartesian Coordinates optimized at B3LYP/6-311G(d,p			
1 2			
O	0.51373000	-1.31075100	-0.05910600
O	0.27467300	3.41725400	0.34322300
O	6.04479500	0.56211000	-1.20022900
O	-5.59690300	-1.83264200	-0.96278400
O	6.78532000	-1.27387800	0.88906100
O	-7.11497200	0.01184000	0.64023500
C	0.76547200	1.01940200	0.39332000
C	-0.71219200	0.60040700	0.60529000
C	1.35317400	-0.19752700	-0.37731400
C	-0.53471800	-0.90122700	0.83295300
C	-1.64162500	0.92611000	-0.59418600
C	0.99534900	2.36673500	-0.29117600
C	2.79759300	-0.50021400	-0.05060100
C	-3.09344000	0.67049000	-0.28467800
C	3.82326700	0.12161700	-0.74247300
C	3.12408400	-1.42279800	0.96840400
C	-3.75413900	-0.45744700	-0.73874200
C	-3.81722300	1.60227400	0.50021900
C	5.17439500	-0.11953500	-0.44895100
C	4.44585700	-1.68076100	1.27896900
C	5.48713200	-1.04369900	0.59728300
C	-5.10792900	-0.70287300	-0.44681400
C	-5.14779200	1.38888200	0.80657100
C	-5.81701100	0.24866100	0.35282100
C	7.47852000	0.40149900	-1.15684700
C	-6.96976300	-2.26676100	-0.85835000
H	1.23274200	1.05425200	1.38594600
H	-1.10747900	1.08877800	1.49879900
H	1.27729400	-0.01674800	-1.45768800
H	-0.24421300	-1.10312400	1.87258300
H	-1.41621300	-1.50084800	0.60301100
H	-1.34112500	0.34293800	-1.46868600
H	-1.52621800	1.98329700	-0.84226100
H	0.63378700	2.33654300	-1.32172900
H	2.06795800	2.59112400	-0.33349600
H	3.62159200	0.80788100	-1.55651700
H	2.33006200	-1.94723900	1.48195500
H	0.72375900	3.65015700	1.16188100
H	-3.25351200	-1.19096800	-1.35917500
H	-3.32353300	2.50060400	0.85265700
H	4.69376500	-2.39380100	2.05921100
H	-5.69466300	2.11299800	1.40236900
H	6.85728600	-1.92008500	1.60271800
H	-7.47412600	0.72843600	1.17845300
H	7.87396100	0.70572500	-0.18861000
H	7.84555400	1.06044300	-1.93968700
H	7.75880400	-0.63002500	-1.36806700

H	-7.64211600	-1.52898700	-1.29476000
H	-7.23876800	-2.44993000	0.18113000
H	-7.00181500	-3.19230300	-1.42744000
Frequency and Energy at B3LYP/6-311G(d,p)in gas phase			
Zero-point correction=	0.412087 (Hartree/Particle)		
Thermal correction to Energy=	0.437703		
Thermal correction to Enthalpy=	0.438647		
Thermal correction to Gibbs Free Energy=	0.352636		
Sum of electronic and zero-point Energies=	-1227.522241		
Sum of electronic and thermal Energies=	-1227.496625		
Sum of electronic and thermal Enthalpies=	-1227.495681		
Sum of electronic and thermal Free Energies=	-1227.581692		

**Name of compound (10)**	**Lignan-A**		
Cartesian Coordinates optimized at B3LYP/6-311G(d,p)			
0 1			
C	0.77030900	-1.03387700	-0.37483200
H	1.17467400	-1.43032000	-1.31559200
C	-0.67233100	0.79072200	-1.07646600
H	-1.22431200	1.43371600	-0.38503100
H	-1.07032600	0.93812300	-2.08180300
C	1.41778600	0.35863200	-0.18224300
O	0.70243900	1.18429500	-1.10113800
H	2.17589300	-2.13556400	0.82835900
H	0.69232800	-1.69483700	1.68317100
O	0.49057600	-3.31997700	0.49884200
C	1.08740300	-2.03906200	0.72712800
C	2.89755900	0.43061100	-0.47178900
C	3.81785800	0.39671600	0.58584300
C	3.37311600	0.50091400	-1.77963300
C	5.18326400	0.41418800	0.32882300
H	3.45402800	0.36765500	1.60568900
C	4.74276000	0.52524900	-2.03950400
H	2.66536400	0.56309300	-2.59626000
C	5.65501100	0.47691000	-0.99464400
H	5.12258900	0.58883900	-3.05229800
O	6.99333400	0.50078000	-1.24572500
H	7.44279300	0.47230300	-0.39069500
C	5.82057300	0.35444600	2.64476400
H	6.75459200	0.34307000	3.20422800
H	5.24426000	-0.54633500	2.88221300
H	5.24113600	1.24157100	2.92225200
O	6.18431000	0.38605900	1.27197300
H	1.22730800	0.69646000	0.84661100
C	-1.66261500	-0.93266800	0.58755900
H	-1.53799500	-1.98122900	0.87848200
C	-0.72919600	-0.69153300	-0.61633900
H	-1.11389600	-1.32176300	-1.42012300
C	-3.13104100	-0.71431400	0.25154900
C	-3.87266100	-1.73741000	-0.33713100
C	-3.75752400	0.51359200	0.51116000
C	-5.21103400	-1.54803100	-0.68039300

H	-3.40718600	-2.69900100	-0.52654400
C	-5.09166700	0.70375300	0.17379600
H	-3.18963900	1.30154400	0.98662900
C	-5.82896800	-0.33081300	-0.43088400
H	-5.79422200	-2.33952700	-1.13555700
H	0.86272200	-3.67582600	-0.31446300
O	-7.13647100	-0.14242200	-0.75773500
H	-7.36643600	0.76098500	-0.50249200
C	-5.15943200	2.97901600	0.94159200
H	-5.90451900	3.77147400	0.98693500
H	-4.32042400	3.30182100	0.31653900
H	-4.79846300	2.76168400	1.95241500
O	-5.81767900	1.85254000	0.37636300
O	-1.22336000	-0.07979400	1.65639500
H	-1.77046600	-0.27203100	2.42540600

Frequency and Energy at B3LYP/6-311G(d,p)in gas phase

Zero-point correction=	0.418001 (Hartree/Particle)
Thermal correction to Energy=	0.444307
Thermal correction to Enthalpy=	0.445252
Thermal correction to Gibbs Free Energy=	0.359509
Sum of electronic and zero-point Energies=	-1303.024042
Sum of electronic and thermal Energies=	-1302.997735
Sum of electronic and thermal Enthalpies=	-1302.996791
Sum of electronic and thermal Free Energies=	-1303.082533

Energy at ROB3LYP/6-311++G(2df,2p): in gas phase	HF=-1303.5512601
Energy at ROB3LYP/6-311++G(2df,2p): in H2O	HF=-1303.5704633
Energy at ROB3LYP/6-311++G(2df,2p): in ETHANOL	HF=-1303.5695482

**Name of radical**	**Lignan-A-O4-H**

Cartesian Coordinates optimized at B3LYP/6-311G(d,p)

0 2

C	0.83088000	-1.00503900	-0.46744100
H	1.24676100	-1.32093000	-1.43337700
C	-0.63427300	0.82058300	-1.11156700
H	-1.26238900	1.43095200	-0.45829100
H	-0.95498100	0.97516300	-2.14344000
C	1.44198100	0.38646800	-0.15988900
O	0.72487400	1.26774800	-1.01810400
H	2.24204500	-2.19149000	0.65212400
H	0.76032500	-1.81165800	1.53758000
O	0.55489900	-3.34519200	0.23812200
C	1.15364300	-2.08722400	0.55871400
C	2.91929000	0.50041900	-0.43216500
C	3.83151600	0.34256700	0.62163200
C	3.39009600	0.72927000	-1.74839800
C	5.19950200	0.38591200	0.38990100
H	3.44776800	0.19949400	1.62442000
C	4.73269600	0.77875000	-2.00912900
H	2.66257700	0.88410500	-2.53498600
C	5.72849500	0.60425100	-0.97103700
H	5.11600400	0.95615800	-3.00724500
O	6.94941700	0.63984000	-1.19562800

C	5.75720000	0.04945300	2.68205900
H	6.68441000	-0.02883600	3.24558500
H	5.18071700	-0.87452700	2.79471400
H	5.17165600	0.89590600	3.05508700
O	6.14731300	0.25109300	1.32724600
H	1.24024700	0.63617500	0.89068000
C	-1.59055000	-0.95791100	0.51358200
H	-1.46301700	-2.01681700	0.76266900
C	-0.67234300	-0.67186900	-0.69281000
H	-1.05507200	-1.28530300	-1.51012000
C	-3.06320200	-0.72759000	0.20681800
C	-3.80926100	-1.72248800	-0.42315800
C	-3.68975200	0.48300800	0.53800200
C	-5.15299600	-1.52105600	-0.73698200
H	-3.34380500	-2.67157900	-0.66757300
C	-5.02958400	0.68450600	0.23089200
H	-3.11817600	1.24829500	1.04506200
C	-5.77171200	-0.32101600	-0.41586300
H	-5.73996500	-2.29050100	-1.22386600
H	0.94643500	-3.65943100	-0.58316600
O	-7.08385400	-0.12229700	-0.71353400
H	-7.31506600	0.76488400	-0.40740600
C	-5.09707200	2.91462300	1.12221100
H	-5.84544300	3.69910400	1.21982100
H	-4.26730300	3.27615500	0.50615100
H	-4.72334400	2.64349200	2.11523800
O	-5.75660800	1.81728400	0.50290600
O	-1.13332800	-0.14599400	1.60598000
H	-1.67940700	-0.35236200	2.37219100

Frequency and Energy at B3LYP/6-311G(d,p)in gas phase

Zero-point correction=	0.405082 (Hartree/Particle)
Thermal correction to Energy=	0.431148
Thermal correction to Enthalpy=	0.432092
Thermal correction to Gibbs Free Energy=	0.346255
Sum of electronic and zero-point Energies=	-1302.395081
Sum of electronic and thermal Energies=	-1302.369016
Sum of electronic and thermal Enthalpies=	-1302.368071
Sum of electronic and thermal Free Energies=	-1302.453908

Energy at ROB3LYP/6-311++G(2df,2p): in GAS PHASE	HF=-1302.9056059
Energy at ROB3LYP/6-311++G(2df,2p): in H2O	HF=-1302.931184
Energy at ROB3LYP/6-311++G(2df,2p): in ETHANOL	HF=-1302.9299447

**Name of radical**	**Lignan-A-O4’-H**

Cartesian Coordinates optimized at B3LYP/6-311G(d,p)

0 2

C	0.92992600	1.80372200	0.09352500
H	1.49262100	1.60002300	1.01115800
C	-0.53391200	0.81061100	-1.52827500
H	-1.24191700	1.58205900	-1.84355300
H	-0.90632900	-0.16180500	-1.85940500
C	1.73200100	1.34457200	-1.15449700
O	0.74128400	1.06486300	-2.14594400

H	1.63251800	3.83580800	-0.08082200
H	0.08844000	3.55913200	-0.88606000
O	-0.06893100	3.85505300	1.10650400
C	0.67214900	3.31322300	0.00143900
C	2.64872100	0.15457000	-0.89845000
C	3.74881100	0.30383400	-0.03675000
C	2.44186200	-1.07065000	-1.52074200
C	4.60178900	-0.76411000	0.20133300
H	3.93675800	1.26104300	0.43562100
C	3.29795700	-2.14857000	-1.27989400
H	1.61690500	-1.17719200	-2.21226600
C	4.37551300	-2.00830100	-0.42101400
H	3.14192900	-3.10693900	-1.76042300
O	5.21281900	-3.05437300	-0.18453500
H	5.89455500	-2.74347400	0.42567700
C	6.05745100	0.45980900	1.67116500
H	6.95551500	0.24356400	2.24728900
H	5.26267900	0.78912500	2.34962600
H	6.27229600	1.25350500	0.94744900
O	5.70737600	-0.75144700	1.01768500
H	2.33997700	2.16932400	-1.54581500
C	-1.56142400	1.15841300	0.82982700
H	-1.24002000	1.24688100	1.87949300
C	-0.29531600	0.85949500	-0.01454900
H	0.06852400	-0.12314300	0.30135900
C	-2.54060700	-0.00052400	0.76282400
C	-2.18298300	-1.25599700	1.31819400
C	-3.79464300	0.16734300	0.16748500
C	-3.04537500	-2.31681200	1.26764500
H	-1.21597300	-1.37054200	1.79767100
C	-4.69217600	-0.89124200	0.09849800
H	-4.04368500	1.14467900	-0.21866300
C	-4.35356200	-2.21505800	0.65214200
H	-2.79254100	-3.28206000	1.69023200
H	0.42665500	3.70143300	1.91756200
O	-5.14523500	-3.17107200	0.59633200
C	-6.35071100	0.40742700	-1.01423200
H	-7.35301000	0.21610900	-1.39144100
H	-5.70013300	0.71853200	-1.83743900
H	-6.38573600	1.19663700	-0.25697400
O	-5.91199100	-0.82647300	-0.45065900
O	-2.22212400	2.34519400	0.42926500
H	-1.68842500	3.08280300	0.76648700

Frequency and Energy at B3LYP/6-311G(d,p)in gas phase

Zero-point correction=	0.405605 (Hartree/Particle)
Thermal correction to Energy=	0.431179
Thermal correction to Enthalpy=	0.432123
Thermal correction to Gibbs Free Energy=	0.347215
Sum of electronic and zero-point Energies=	-1302.396665
Sum of electronic and thermal Energies=	-1302.371091
Sum of electronic and thermal Enthalpies=	-1302.370147
Sum of electronic and thermal Free Energies=	-1302.455055

Energy at ROB3LYP/6-311++G(2df,2p): in GAS PHASE		HF=-1302.9061393
Energy at ROB3LYP/6-311++G(2df,2p): in H2O		HF=-1302.933271
Energy at ROB3LYP/6-311++G(2df,2p): in ETHANOL		HF=-1302.9318901
**Name of radical**	**Lignan-A-C7-H**	
Cartesian Coordinates optimized at B3LYP/6-311G(d,p)		

0 2			
C	-0.82694200	0.96780400	-0.25825500
H	-1.20366600	1.54416200	-1.12005100
C	0.59597700	-0.87924200	-0.92779000
H	1.42375200	-1.47895900	-0.55696700
H	0.53112000	-0.99259000	-2.01595300
C	-1.51004800	-0.37625900	-0.21958500
O	-0.60857600	-1.39615300	-0.34599600
H	-2.14989400	1.91592000	1.18415200
H	-0.61917200	1.36895800	1.86146100
O	-0.49041800	3.13660200	0.87706000
C	-1.07592900	1.83597600	0.99124800
C	-2.88416100	-0.69645600	-0.17443000
C	-3.89800700	0.31015500	-0.18658900
C	-3.30887100	-2.05296600	-0.14033100
C	-5.23417600	-0.03887100	-0.15490100
H	-3.62085400	1.35222600	-0.25219800
C	-4.65322200	-2.38286300	-0.10547900
H	-2.55947900	-2.83213100	-0.14123000
C	-5.63132100	-1.39191100	-0.10739800
H	-4.97271600	-3.41810200	-0.07598200
O	-6.95348600	-1.72297000	-0.06981100
H	-7.45159000	-0.89507100	-0.08469500
C	-6.02532200	2.23094600	-0.20006200
H	-6.99439000	2.72768100	-0.19369200
H	-5.48050400	2.51262000	-1.10811700
H	-5.45228700	2.54182500	0.68054900
O	-6.29559600	0.83849000	-0.17258600
C	1.65735100	0.86975800	0.63609900
H	1.50249500	1.90388100	0.95901600
C	0.67563400	0.60554000	-0.52835900
H	1.04370700	1.19470600	-1.36948800
C	3.11242300	0.72873000	0.20837000
C	3.76194400	1.78615300	-0.42650800
C	3.81822000	-0.46171300	0.43612300
C	5.08652000	1.66680000	-0.84629800
H	3.23533200	2.72063400	-0.59050800
C	5.13924400	-0.58206600	0.02301200
H	3.32190100	-1.27394800	0.94969900
C	5.78285900	0.48631400	-0.62820600
H	5.59915500	2.48558000	-1.33673700
H	-0.91682000	3.59137600	0.14366700
O	7.07732400	0.36650100	-1.02973400
H	7.37144000	-0.52038400	-0.78241700
C	5.38618800	-2.83442100	0.82334200
H	6.17841200	-3.58095000	0.83595600
H	4.52843700	-3.22259000	0.26441200

H	5.08076800	-2.61105300	1.85088800
O	5.93762800	-1.68736600	0.18865900
O	1.32235800	-0.03118000	1.69772500
H	1.88080300	0.18637100	2.45167900

Frequency and Energy at B3LYP/6-311G(d,p)in gas phase

Zero-point correction= 0.404804 (Hartree/Particle)
Thermal correction to Energy= 0.431043
Thermal correction to Enthalpy= 0.431987
Thermal correction to Gibbs Free Energy= 0.346404
Sum of electronic and zero-point Energies= -1302.397049
Sum of electronic and thermal Energies= -1302.370809
Sum of electronic and thermal Enthalpies= -1302.369865
Sum of electronic and thermal Free Energies= -1302.455448

Energy at ROB3LYP/6-311++G(2df,2p): in GAS PHASE	HF=-1302.9088836
**Name of anion**	**Lignan-A-O4-H**

Cartesian Coordinates optimized at B3LYP/6-311G(d,p

-1 1

C	-0.75844400	1.07815100	0.10387200
H	-1.11501200	1.80755200	-0.63263500
C	0.57534700	-0.66817400	-0.93397300
H	0.89861500	-1.45731700	-0.24046900
H	1.15622700	-0.75246500	-1.85557700
C	-1.55164100	-0.22853300	-0.16952600
O	-0.79168600	-0.82896000	-1.25875400
H	-2.12544600	1.70399400	1.62632800
H	-0.61673700	1.03096800	2.26554100
O	-0.45401400	2.96300100	1.67471600
C	-1.04000000	1.65995700	1.48247900
C	-2.99892400	-0.07582100	-0.49678600
C	-3.98164600	-0.73780300	0.27916300
C	-3.45223800	0.71512700	-1.56069300
C	-5.33176700	-0.60185700	0.02039000
H	-3.64269300	-1.36570300	1.09844500
C	-4.80273200	0.85626700	-1.83774700
H	-2.72467100	1.20959600	-2.20236000
C	-5.84953400	0.22430500	-1.07202200
H	-5.13590800	1.46831700	-2.67198600
O	-7.07427700	0.35575200	-1.30520200
C	-5.92348000	-2.05969700	1.81281100
H	-6.83729100	-2.46171500	2.25742600
H	-5.36229700	-1.51538100	2.58936200
H	-5.29680900	-2.89826700	1.47001900
O	-6.32552200	-1.22838100	0.76078500
H	-1.44855900	-0.88390900	0.71076500
C	1.73307800	0.76542700	0.90515400
H	1.61567100	1.73919600	1.39266900
C	0.71806900	0.72299400	-0.25344100
H	1.08288800	1.44717900	-0.98674600
C	3.17614600	0.64029300	0.43381000
C	3.91256400	1.76992600	0.08155800
C	3.78298700	-0.61962900	0.32807900
C	5.22519800	1.65881400	-0.38106400

H	3.46063100	2.75245800	0.16924000
C	5.08662800	-0.73351500	-0.13391400
H	3.21613000	-1.49134200	0.62410700
C	5.81972700	0.41085700	-0.49366900
H	5.80312600	2.53385800	-0.65479000
H	-0.84876500	3.54045500	1.01329600
O	7.10522000	0.28949400	-0.94125400
H	7.30289200	-0.65626900	-0.95450900
C	5.10284100	-3.13218300	-0.04022300
H	5.81305800	-3.92682300	-0.26542900
H	4.22451000	-3.23115900	-0.68615800
H	4.79088600	-3.21015200	1.00678400
O	5.78739400	-1.91098400	-0.28587300
O	1.40360800	-0.28002800	1.83713300
H	1.93069900	-0.13166800	2.62872200

Frequency and Energy at B3LYP/6-311G(d,p)in gas phase

Zero-point correction= 0.403220 (Hartree/Particle)
Thermal correction to Energy= 0.429267
Thermal correction to Enthalpy= 0.430211
Thermal correction to Gibbs Free Energy= 0.344840
Sum of electronic and zero-point Energies= -1302.461764
Sum of electronic and thermal Energies= -1302.435717
Sum of electronic and thermal Enthalpies= -1302.434773
Sum of electronic and thermal Free Energies= -1302.520144

**Name of anion**	**Lignan-A-O4'-H**

Cartesian Coordinates optimized at B3LYP/6-311G(d,p

-1 1

C	0.79687400	0.78527600	0.80853300
H	1.21086600	0.72648300	1.82693300
C	-0.69529400	-1.12199900	0.76297800
H	-1.31553600	-1.46035600	-0.06739800
H	-1.03327300	-1.62047100	1.67370600
C	1.39499400	-0.41631600	0.03717000
O	0.66982500	-1.53126400	0.54162500
H	2.25556200	2.22259700	0.14398500
H	0.72304500	2.28251100	-0.74821600
O	0.67401200	3.23739200	1.03726600
C	1.16243700	2.15171200	0.24085400
C	2.87662500	-0.64148400	0.23350100
C	3.79062800	-0.09403400	-0.67848200
C	3.36433400	-1.36715900	1.31881500
C	5.15705800	-0.25567100	-0.48831200
H	3.41711500	0.44783800	-1.53862400
C	4.73639600	-1.53662100	1.50729400
H	2.65787900	-1.81962400	2.00240000
C	5.64022100	-0.98090900	0.61329300
H	5.12320000	-2.10617700	2.34458700
O	6.98678100	-1.14393000	0.79111800
H	7.41752000	-0.68956200	0.05539700
C	5.76589900	0.96687900	-2.46378400
H	6.69059000	1.25632100	-2.96191100
H	5.20166800	1.86619200	-2.19374700

H	5.16364700	0.35390300	-3.14327000
O	6.15153100	0.23370400	-1.31141300
H	1.18224500	-0.28137800	-1.03230000
C	-1.61047000	1.12420900	-0.16997300
H	-1.48860100	2.20084700	0.00399700
C	-0.71568300	0.42169800	0.87934600
H	-1.11500500	0.72275200	1.85005900
C	-3.08361500	0.81278800	-0.07335100
C	-3.94223200	1.64951200	0.65543200
C	-3.66841000	-0.31827400	-0.68276000
C	-5.29395400	1.37463900	0.78545600
H	-3.53313100	2.54345900	1.12555100
C	-5.02032100	-0.59306200	-0.58640900
H	-3.07248500	-1.00077700	-1.28302300
C	-5.94254800	0.24763000	0.16319200
H	-5.94527700	2.04704300	1.33763000
H	0.93130100	3.06128100	1.94776000
O	-7.18347100	0.02356300	0.24463500
C	-6.25558400	-2.62198400	-0.56466200
H	-6.60123900	-3.37615400	-1.27787300
H	-7.10118600	-2.12410700	-0.08666800
H	-5.63365400	-3.11616400	0.19805800
O	-5.48844500	-1.68319300	-1.31322300
O	-1.06856700	0.79774500	-1.47530500
H	-1.81270900	0.87043400	-2.08343600

Frequency and Energy at B3LYP/6-311G(d,p)in gas phase

Zero-point correction=	0.403326 (Hartree/Particle)
Thermal correction to Energy=	0.429494
Thermal correction to Enthalpy=	0.430438
Thermal correction to Gibbs Free Energy=	0.345052
Sum of electronic and zero-point Energies=	-1302.474081
Sum of electronic and thermal Energies=	-1302.447912
Sum of electronic and thermal Enthalpies=	-1302.446968
Sum of electronic and thermal Free Energies=	-1302.532354

**Name of anion**	**Lignan-A-O11-H**

Cartesian Coordinates optimized at B3LYP/6-311G(d,p

-1 1

C	0.94656900	1.56106200	0.04354400
H	1.48706800	1.38991400	0.97957700
C	-0.50676800	0.51924900	-1.52936600
H	-1.11439500	1.35708100	-1.88387500
H	-0.97449900	-0.42098500	-1.83172700
C	1.76016500	1.06661500	-1.18133700
O	0.81114800	0.57040900	-2.12999600
H	1.69696300	3.56677600	-0.05981300
H	0.28651000	3.29085000	-1.08678200
O	-0.07644500	3.63774100	0.92917300
C	0.70400400	3.08736200	-0.08433000
C	2.82305900	0.02026600	-0.86957000
C	3.89298000	0.35752700	-0.02413700
C	2.78987500	-1.25377700	-1.42670000
C	4.88687500	-0.56811600	0.25620400

H	3.92891300	1.34885400	0.41134100
C	3.79183700	-2.18765800	-1.14593900
H	1.97262100	-1.50332200	-2.09012900
C	4.84279500	-1.85539500	-0.30727900
H	3.76759300	-3.18354100	-1.57410900
O	5.83220100	-2.76195500	-0.02492600
H	6.43861500	-2.31875100	0.58225800
C	6.07772400	0.88654900	1.74969400
H	6.98043300	0.83351700	2.35812600
H	5.21074900	1.05737600	2.39706200
H	6.16616400	1.71904800	1.04253500
O	5.98186800	-0.35735900	1.07538800
H	2.27231600	1.92030600	-1.65015000
C	-1.57615000	1.09375100	0.76353600
H	-1.24314600	1.13150700	1.83089400
C	-0.29707100	0.63556200	-0.02585900
H	0.02117500	-0.34450300	0.35504500
C	-2.63300400	-0.03596600	0.72133600
C	-2.42819400	-1.33032100	1.20252500
C	-3.88719500	0.28950600	0.19488100
C	-3.44340200	-2.28989500	1.14965600
H	-1.46980500	-1.60254100	1.63424300
C	-4.89648200	-0.66025600	0.14063400
H	-3.99165000	1.31700400	-0.13627300
C	-4.68234400	-1.96207200	0.61644400
H	-3.29033800	-3.29783000	1.52091600
O	-5.69420400	-2.89428200	0.55927500
H	-6.44511100	-2.43876900	0.15752200
C	-6.47093100	0.84173300	-0.86016000
H	-7.50282700	0.80364100	-1.21189100
H	-5.80911300	1.10899000	-1.69103900
H	-6.37708800	1.60414700	-0.07921300
O	-6.17544200	-0.45143500	-0.35948400
O	-2.07525900	2.27636600	0.32197000
H	-1.00814300	3.21099800	0.75161100

Frequency and Energy at B3LYP/6-311G(d,p)in gas phase

Zero-point correction= 0.402585 (Hartree/Particle)
Thermal correction to Energy= 0.427804
Thermal correction to Enthalpy= 0.428748
Thermal correction to Gibbs Free Energy= 0.345180
Sum of electronic and zero-point Energies= -1302.477064
Sum of electronic and thermal Energies= -1302.451846
Sum of electronic and thermal Enthalpies= -1302.450901
Sum of electronic and thermal Free Energies= -1302.534470

Name of anion	**Lignan-A-C7-H**

Cartesian Coordinates optimized at B3LYP/6-311G(d,p

-1 1

C	-0.88455600	0.89115100	-0.01193200
H	-1.33368000	1.09389500	-1.01116600
C	0.63177200	-0.63322800	1.03011700
H	0.89214200	-0.03436300	1.92056300
H	1.36881500	-1.43290000	0.91915400

C	-1.60042600	-0.20921200	0.71971500
O	-0.64519300	-1.21262900	1.14831500
H	-2.02404400	2.42003700	0.98554800
H	-0.44692600	2.13219200	1.72660600
O	-0.38211700	3.36515000	0.09752200
C	-0.97228800	2.20817300	0.77529300
C	-2.91910300	-0.61717000	0.53484700
C	-3.90322200	0.21780800	-0.11396900
C	-3.39798800	-1.90279000	0.97118800
C	-5.21394600	-0.20808200	-0.27019700
H	-3.61118200	1.19511700	-0.47594700
C	-4.71656400	-2.28934600	0.79637400
H	-2.69412400	-2.57890100	1.43920600
C	-5.65260500	-1.45828100	0.18150200
H	-5.04992100	-3.26636100	1.13711500
O	-6.98734700	-1.85147700	0.03322300
H	-7.41411000	-1.11270600	-0.41522800
C	-5.90257200	1.84843000	-1.31516500
H	-6.81582000	2.26524700	-1.74540200
H	-5.11460100	1.84329300	-2.07944400
H	-5.57335300	2.48247800	-0.48157700
O	-6.22118300	0.54559500	-0.88557900
C	1.75026800	1.18018600	-0.50810700
H	1.53900900	1.70367500	-1.45417500
C	0.53478400	0.27243100	-0.20613700
H	0.43803500	-0.39878500	-1.06847600
C	3.01585100	0.36131300	-0.72776700
C	3.16790300	-0.44178500	-1.85768300
C	4.05224900	0.40277200	0.21290000
C	4.32313500	-1.20034700	-2.05219600
H	2.37851800	-0.48145900	-2.60057400
C	5.20026900	-0.35406700	0.02228600
H	3.93162300	1.04578600	1.07253600
C	5.34504800	-1.16409400	-1.11494500
H	4.44528800	-1.82670200	-2.92830600
H	-0.66689200	3.31289000	-0.82179700
O	6.48544700	-1.90184500	-1.29424400
H	7.03891400	-1.73433000	-0.52037400
C	6.20745300	0.33970700	2.08689800
H	7.13794600	0.14622600	2.62034800
H	5.35979800	0.01136700	2.69764300
H	6.11435500	1.41411800	1.89467300
O	6.28657500	-0.39718900	0.87623100
O	2.01467300	2.14448500	0.50519400
H	1.30060100	2.80204600	0.42566200

Frequency and Energy at B3LYP/6-311G(d,p)in gas phase	
Zero-point correction=	0.401493 (Hartree/Particle)
Thermal correction to Energy=	0.427829
Thermal correction to Enthalpy=	0.428773
Thermal correction to Gibbs Free Energy=	0.343227
Sum of electronic and zero-point Energies=	-1302.421121
Sum of electronic and thermal Energies=	-1302.394785

Sum of electronic and thermal Enthalpies=	-1302.393840		
Sum of electronic and thermal Free Energies=	-1302.479387		
**Name of cationic radical**	**Lignan-A**		
Cartesian Coordinates optimized at B3LYP/6-311G(d,p			
1 2			
C	0.89634500	1.23446300	0.27163300
H	1.40228500	0.93537700	1.19436900
C	-0.57016000	0.31739700	-1.38583800
H	-0.93634500	1.23885000	-1.85354800
H	-1.20633200	-0.51127100	-1.69696600
C	1.69235100	0.70820100	-0.98716300
O	0.76106500	0.01921200	-1.81355300
H	1.87882200	3.15545800	0.18395000
H	0.34496900	3.11352200	-0.68122100
O	0.14829500	3.37009200	1.30927300
C	0.85388000	2.76912600	0.22054200
C	2.85599300	-0.19164900	-0.64997600
C	4.10097800	0.37217600	-0.33950500
C	2.70255200	-1.59215300	-0.63437900
C	5.16818900	-0.44742200	-0.00188500
H	4.23327800	1.44586600	-0.38517100
C	3.75866600	-2.41990300	-0.29794300
H	1.74822200	-2.01348700	-0.91820700
C	4.99821500	-1.86502000	0.02618100
H	3.65876200	-3.49788600	-0.28860300
O	6.02382400	-2.65828900	0.34853700
H	6.80303400	-2.10851400	0.52521000
C	6.76346500	1.33398900	0.27891700
H	7.81553400	1.38543800	0.54684500
H	6.16874100	1.89019100	1.00823200
H	6.61583100	1.74259300	-0.72393100
O	6.42321100	-0.05852500	0.30923800
H	2.08164300	1.57551000	-1.53973400
C	-1.71110000	1.03575100	0.84776900
H	-1.46874400	1.12155300	1.91646800
C	-0.44872600	0.47032300	0.12983600
H	-0.27225700	-0.52934100	0.54108200
C	-2.86557700	0.06176000	0.72308100
C	-2.79107000	-1.21380400	1.33400900
C	-4.01362600	0.42295300	0.01748300
C	-3.83258700	-2.11502000	1.24112800
H	-1.90894000	-1.48630800	1.90154300
C	-5.06944700	-0.47404200	-0.08642600
H	-4.06588000	1.41086900	-0.41544200
C	-4.98301500	-1.76402100	0.52743900
H	-3.79307100	-3.08856500	1.71250200
H	0.65671300	3.28024900	2.12167300
O	-5.99653600	-2.62381500	0.42940300
H	-6.70946600	-2.21493800	-0.08677300
C	-6.48214400	0.98912800	-1.37369900
H	-7.47719100	0.90561200	-1.80216100
H	-5.74656000	1.15662100	-2.16349500

H	-6.45349200	1.80398100	-0.64715700
O	-6.23687400	-0.26946400	-0.72141100
O	-2.11562900	2.28825200	0.33871800
H	-1.56747300	2.95553200	0.78166600
Frequency and Energy at B3LYP/6-311G(d,p)in gas phase			
Zero-point correction= 0.418675 (Hartree/Particle)<br>Thermal correction to Energy= 0.444486<br>Thermal correction to Enthalpy= 0.445430<br>Thermal correction to Gibbs Free Energy= 0.359950<br>Sum of electronic and zero-point Energies= -1302.775816<br>Sum of electronic and thermal Energies= -1302.750005<br>Sum of electronic and thermal Enthalpies= -1302.749061<br>Sum of electronic and thermal Free Energies= -1302.834541			

Table S4: Cartesian coordinates of all of the Int, TS of the selective compounds and HOO• optimized at B3LYP/6-311G(d,p) level of theory in the gas phase

**Name of compound**			**Int1-1-C7'-H-OOH**
Cartesian Coordinates optimized at B3LYP/6-311G(d,p)			
0 2			
C	0.77957800	-1.54666500	0.00950300
C	1.81630100	-2.45197400	-0.16004300
C	3.11331200	-1.97378900	-0.43093100
C	3.33106600	-0.61041000	-0.53001900
C	-0.18027700	0.80055400	0.15425100
C	2.28045000	0.30962300	-0.36347800
C	0.99244100	-0.15654800	-0.09739800
C	1.87868600	4.13975000	0.02922100
C	-0.98085700	3.22245700	0.27520300
H	-1.14253300	3.02992000	1.34831900
C	1.53758100	2.66133900	0.23230300
C	0.11799300	2.27508600	-0.25126100
H	1.60977500	2.45953800	1.30979700
H	0.10390500	2.35773400	-1.34568000
O	1.58306300	-3.78783500	-0.06403800
H	2.42612800	-4.23745800	-0.21111000
H	4.32516000	-0.23022500	-0.73175700
C	2.57137200	1.78583400	-0.48737000
H	3.56695700	2.00756000	-0.10030900
H	2.58796000	2.06917600	-1.55017600
H	-0.32423500	0.82124300	1.24259100
C	5.39475700	-2.58930700	-0.85468300
H	5.46190800	-2.04623200	-1.80314200
H	5.95717600	-3.51837500	-0.92812500
H	5.81093400	-1.97402900	-0.05021300
O	4.05184000	-2.96417600	-0.57202100
H	-0.21679300	-1.94239300	0.16826900
C	-1.47816100	0.25163400	-0.43387200
C	-1.67039800	0.12557700	-1.81109800
C	-2.50066400	-0.17945000	0.42818300
C	-2.85238400	-0.40414900	-2.32535400
H	-0.89239600	0.44029900	-2.49655600
C	-3.67968300	-0.71417200	-0.08176700
C	-3.86290200	-0.82703600	-1.47143400
H	-3.00774000	-0.49914400	-3.39331000
O	-5.01461100	-1.34794000	-1.97286000
H	-5.57403000	-1.58029200	-1.21954200
H	-2.34671600	-0.11432700	1.49760400
C	-4.64892000	-1.12164200	2.07785500
H	-3.80241200	-1.71152900	2.44307800
H	-5.57826400	-1.54645900	2.45299900
H	-4.55205900	-0.08900200	2.42847700
O	-4.73941300	-1.17444300	0.65757000
O	3.24208300	4.33527000	0.40907800
O	0.88867500	-1.27415800	3.12459300
H	0.98766900	-1.36558500	2.14719600
O	-0.35699700	-0.86566400	3.32354800

H	3.44509400	5.27009600	0.31668800
O	-2.20012500	3.16784600	-0.44982500
H	-2.52814300	2.26277200	-0.41932500
H	-0.66072800	4.25964300	0.18143800
H	1.72145500	4.41276700	-1.02609000
H	1.23005600	4.77281600	0.64364100

Frequency and Energy at B3LYP/6-311G(d,p)in gas phase

Zero-point correction= 0.429084 (Hartree/Particle)

Thermal correction to Energy= 0.458787

Thermal correction to Enthalpy= 0.459731

Thermal correction to Gibbs Free Energy= 0.365084

Sum of electronic and zero-point Energies= -1378.742756

Sum of electronic and thermal Energies= -1378.713053

Sum of electronic and thermal Enthalpies= -1378.712109

Sum of electronic and thermal Free Energies= -1378.806756

Name of compound	TS-1-C7’-H-OOH

Cartesian Coordinates optimized at B3LYP/6-311G(d,p)

0 2

C	0.87921700	-1.68779100	0.13591400
C	1.96827300	-2.53348000	0.13227800
C	3.25236600	-2.01054900	-0.13775500
C	3.40711100	-0.65516200	-0.39202900
C	-0.16301600	0.58290400	-0.04634100
C	2.30585600	0.20847100	-0.38528900
C	1.01899900	-0.30560400	-0.13060800
C	1.68013800	4.02278000	-0.44385600
C	-1.13475200	2.95206000	-0.00056300
H	-1.32290300	2.75878000	1.06372700
C	1.42265800	2.56267900	-0.06136800
C	0.02495900	2.05361100	-0.49204500
H	1.50577100	2.48515300	1.02870400
H	-0.02195600	2.08434200	-1.59191300
O	1.81340500	-3.86130700	0.39042700
H	2.68703300	-4.26964400	0.32904300
H	4.38905400	-0.24508000	-0.59324400
C	2.50186700	1.67035800	-0.68892700
H	3.48659400	1.99792800	-0.35344900
H	2.48553700	1.81904900	-1.77971700
H	-0.26235600	0.69948300	1.24573800
C	5.58511500	-2.53336800	-0.37149600
H	5.67578900	-2.10671000	-1.37543400
H	6.19655300	-3.43106200	-0.30129100
H	5.92250200	-1.80230500	0.37001400
O	4.24821500	-2.94838100	-0.11032000
H	-0.09686400	-2.11245500	0.33304700
C	-1.47556500	-0.05976000	-0.45351000
C	-1.68565700	-0.45318900	-1.77840100

C	-2.50706400	-0.27012700	0.47977100
C	-2.89058100	-1.02984100	-2.17604300
H	-0.90090800	-0.30767500	-2.51142600
C	-3.70846300	-0.84967000	0.08447700
C	-3.90871400	-1.23231800	-1.25416600
H	-3.05688100	-1.33179600	-3.20313800
O	-5.08331300	-1.79858000	-1.63830200
H	-5.64481300	-1.85048800	-0.85307200
H	-2.34801300	0.00285600	1.51414700
C	-4.69196100	-0.74841800	2.27450800
H	-3.87541900	-1.28031800	2.77297600
H	-5.64107000	-1.03841900	2.72176600
H	-4.54811100	0.33062400	2.38938500
O	-4.77974400	-1.11181400	0.90043300
O	3.03259600	4.33781600	-0.10536300
O	1.01053900	0.53070200	2.90591100
H	1.07550000	-0.43626300	2.93436700
O	-0.31534200	0.75088900	2.50601700
H	3.17562300	5.26913600	-0.29426800
O	-2.32134600	2.83538100	-0.77591600
H	-2.65546300	1.93811200	-0.67594500
H	-0.85514100	4.00036200	-0.09378600
H	1.50535700	4.15818200	-1.52293100
H	0.99979400	4.68904600	0.09630700

Frequency and Energy at B3LYP/6-311G(d,p)in gas phase

Zero-point correction= 0.423745 (Hartree/Particle)
Thermal correction to Energy= 0.452752
Thermal correction to Enthalpy= 0.453696
Thermal correction to Gibbs Free Energy= 0.362301
Sum of electronic and zero-point Energies= -1378.724799
Sum of electronic and thermal Energies= -1378.695792
Sum of electronic and thermal Enthalpies= -1378.694848
Sum of electronic and thermal Free Energies= -1378.786243

**Name of compound**	**Int2-1-C7’-H-OOH**

Cartesian Coordinates optimized at B3LYP/6-311G(d,p)

0 2

C	0.73388800	-1.59428900	-0.52110500
C	1.79170300	-2.47851200	-0.48015100
C	3.11706800	-1.99178400	-0.43644700
C	3.34652200	-0.61879500	-0.46498100
C	-0.13992600	0.74894200	-0.55812300
C	2.28957800	0.28613400	-0.51971100
C	0.94076500	-0.17919500	-0.52272300
C	1.81056600	4.08644900	-0.11392000
C	-0.95234500	2.79655200	0.69893800
H	-1.03098500	2.09168300	1.53548700
C	1.50864800	2.60127500	0.08130600
C	0.06499800	2.24285700	-0.34287300
H	1.60976000	2.38170800	1.15196800
H	-0.16540500	2.77270000	-1.28169300
O	1.56236800	-3.82073100	-0.47919200

H	2.42357700	-4.25803100	-0.45098800
H	4.35954700	-0.23492500	-0.46278300
C	2.54237200	1.75792000	-0.67182100
H	3.54630100	2.02178800	-0.33490200
H	2.49080100	2.02068700	-1.73985800
H	0.61229800	-1.10284200	1.78024500
C	5.44135800	-2.59375800	-0.38112000
H	5.69965200	-2.04592500	-1.29333900
H	6.01160800	-3.52013900	-0.34043500
H	5.68003900	-1.98035700	0.49399400
O	4.07125100	-2.97528700	-0.38231600
H	-0.26488900	-2.00439800	-0.58063700
C	-1.53784900	0.27361500	-0.75508100
C	-2.23496800	0.56204000	-1.93413100
C	-2.19906000	-0.46557600	0.24818200
C	-3.54542600	0.12018700	-2.12435200
H	-1.74870300	1.13050000	-2.71796800
C	-3.50297900	-0.90323100	0.05958300
C	-4.18724900	-0.61341100	-1.13697900
H	-4.08330700	0.33663200	-3.03950700
O	-5.46324200	-1.04594700	-1.31733400
H	-5.71628400	-1.52404700	-0.51591300
H	-1.68185200	-0.68431400	1.17276100
C	-3.65390000	-1.94625400	2.21754700
H	-2.75885100	-2.56178100	2.08828100
H	-4.40863800	-2.50757800	2.76547500
H	-3.39125500	-1.04119400	2.77346800
O	-4.24478700	-1.62295200	0.96028500
O	3.09837500	4.35065700	0.44833200
O	0.33732000	0.29870400	3.03444900
H	1.03984600	0.27165500	3.69800100
O	0.24213800	-1.10573600	2.68045300
H	3.29974000	5.28009800	0.30900300
O	-2.22372400	3.11534200	0.15388100
H	-2.63493300	2.29376600	-0.13508200
H	-0.57648800	3.73771300	1.10210700
H	1.79859900	4.31922100	-1.18975700
H	1.04964700	4.70797500	0.37138800

Frequency and Energy at B3LYP/6-311G(d,p)in gas phase

Zero-point correction= 0.428039 (Hartree/Particle)
Thermal correction to Energy= 0.458275
Thermal correction to Enthalpy= 0.459219
Thermal correction to Gibbs Free Energy= 0.363746
Sum of electronic and zero-point Energies= -1378.747005
Sum of electronic and thermal Energies= -1378.716769
Sum of electronic and thermal Enthalpies= -1378.715825
Sum of electronic and thermal Free Energies= -1378.811298

**Name of compound**	**Int1-1-O4-H-OOH**
Cartesian Coordinates optimized at B3LYP/6-311G(d,p)	
0 2	

C	1.10860300	-0.60337400	0.42660700
C	2.47643000	-0.78869600	0.31196800
C	3.28567300	0.31265100	-0.03385800
C	2.70349600	1.55274000	-0.24329100
C	-1.00436700	0.79244500	0.41069700
C	1.31583100	1.73595300	-0.12242400
C	0.50883000	0.64685700	0.20877000
C	-1.16422700	4.70237900	0.05490500
C	-3.02884000	2.35936200	0.39812000
H	-3.09010800	2.15056800	1.47911000
C	-0.62189800	3.30094100	0.34722300
C	-1.57574800	2.15863900	-0.08079700
H	-0.45914400	3.23817600	1.43206600
H	-1.61202700	2.15118700	-1.17788800
O	3.00798000	-2.01679100	0.53574200
H	3.96922600	-1.99923100	0.39520400
H	3.31894100	2.40717900	-0.49768000
C	0.73087400	3.10889600	-0.35033700
H	1.42627800	3.87667800	-0.00781000
H	0.59172400	3.28341800	-1.42795100
H	-1.17417200	0.77710600	1.49693200
C	5.51628100	1.07048200	-0.47769000
H	5.28135900	1.47823800	-1.46750800
H	6.50886500	0.62279600	-0.49736500
H	5.49760800	1.88064200	0.26008200
O	4.62469400	0.02651500	-0.12318300
H	0.50844700	-1.46865900	0.68089600
C	-1.76057100	-0.40494200	-0.16130300
C	-1.70194700	-0.72138900	-1.52015400
C	-2.52713000	-1.22723300	0.68147400
C	-2.39114900	-1.81647400	-2.03556500
H	-1.10260400	-0.11251500	-2.18663500
C	-3.21485800	-2.32528300	0.17104700
C	-3.15052400	-2.62532800	-1.20017000
H	-2.34774000	-2.06262200	-3.08974100
O	-3.82082100	-3.69766200	-1.70272600
H	-4.27549900	-4.12150500	-0.96270100
H	-2.57222000	-1.00245000	1.73992900
C	-4.12552200	-2.99063200	2.29344700
H	-3.15581900	-3.04700200	2.79915600
H	-4.76908600	-3.79054000	2.65569400
H	-4.59380100	-2.02355600	2.50542900
O	-3.98895600	-3.20014100	0.89395600
O	-0.15514300	5.65114100	0.40786600
O	5.78064400	-4.07260700	0.29114600
H	6.69972800	-4.36648500	0.14546100
O	5.81712300	-2.74988200	0.19964500
H	-0.50137600	6.53026500	0.23220700
O	-3.99545400	1.61139600	-0.32639800
H	-3.75776900	0.67971000	-0.26969000
H	-3.33386800	3.39571900	0.25777300
H	-1.42172500	4.78187600	-1.01288900
H	-2.07117100	4.89729800	0.63752200

Frequency and Energy at B3LYP/6-311G(d,p)in gas phase
Zero-point correction= 0.407683 (Hartree/Particle)<br>Thermal correction to Energy= 0.435410<br>Thermal correction to Enthalpy= 0.436355<br>Thermal correction to Gibbs Free Energy= 0.342227<br>Sum of electronic and zero-point Energies= -1377.531879<br>Sum of electronic and thermal Energies= -1377.504152<br>Sum of electronic and thermal Enthalpies= -1377.503208<br>Sum of electronic and thermal Free Energies= -1377.597335

**Name of compound**	**TS-1-O4-H-OOH**
Cartesian Coordinates optimized at B3LYP/6-311G(d,p)	

```
0 2
 C    -1.07799000   -0.70786700   -0.34892900
 C    -2.44988600   -1.01711300   -0.24364300
 C    -3.34930100    0.06172800    0.05827100
 C    -2.85655300    1.34744100    0.22283400
 C     0.91228500    0.84820800   -0.38613200
 C    -1.48757000    1.62446100    0.10515000
 C    -0.58439400    0.57895200   -0.18194800
 C     0.77063300    4.76104500   -0.09326300
 C     2.81639500    2.55511700   -0.35564200
 H     2.91895000    2.31652000   -1.42713400
 C     0.34540600    3.31650000   -0.37003400
 C     1.37140300    2.25683900    0.09924400
 H     0.21326300    3.22392300   -1.45682300
 H     1.38605800    2.26841400    1.19668600
 O    -2.85973200   -2.23780300   -0.42410900
 H    -3.96837900   -2.50787300   -0.29547400
 H    -3.53264400    2.16499700    0.44098800
 C    -1.00564400    3.03789800    0.30191300
 H    -1.75078000    3.74511800   -0.06567500
 H    -0.90307300    3.24059300    1.37918500
 H     1.07175500    0.85144100   -1.47379900
 C    -5.62848400    0.67307600    0.45705600
 H    -5.43801600    1.13272200    1.43256800
 H    -6.57491600    0.13722900    0.48271800
 H    -5.66543400    1.45026900   -0.31347600
 O    -4.64491400   -0.30635200    0.15090000
 H    -0.41550400   -1.53703000   -0.56397700
 C     1.76527900   -0.28802800    0.17198000
 C     1.77164800   -0.59433900    1.53440800
 C     2.55369800   -1.06748800   -0.69091400
 C     2.54473200   -1.64016000    2.03347800
 H     1.16133200   -0.01711200    2.21884600
 C     3.32457600   -2.11637800   -0.19721100
 C     3.32421300   -2.40805300    1.17808800
 H     2.55188500   -1.87915600    3.09004800
 O     4.07559900   -3.43082000    1.66492100
 H     4.52895200   -3.83633000    0.91375000
 H     2.54970000   -0.84985200   -1.75174300
 C     4.18979600   -2.75486300   -2.34676100
```

H	3.20497500	-2.88265000	-2.80806200
H	4.86764300	-3.51861700	-2.72377000
H	4.58549900	-1.76401900	-2.59365300
O	4.12687800	-2.94671900	-0.93889200
O	-0.30228900	5.61835400	-0.48638500
O	-5.04210900	-4.29834700	-0.38657100
H	-5.94506000	-4.59003500	-0.19859300
O	-5.13251300	-2.92573700	-0.15604700
H	-0.03005600	6.52675800	-0.33017800
O	3.81780300	1.90911500	0.41588900
H	3.66644800	0.95911500	0.37003300
H	3.03631900	3.61613900	-0.24458800
H	0.99754600	4.87995500	0.97774000
H	1.67230300	5.01410800	-0.66093100

Frequency and Energy at B3LYP/6-311G(d,p)in gas phase

Zero-point correction= 0.423810 (Hartree/Particle)
Thermal correction to Energy= 0.452809
Thermal correction to Enthalpy= 0.453753
Thermal correction to Gibbs Free Energy= 0.360631
Sum of electronic and zero-point Energies= -1378.728711
Sum of electronic and thermal Energies= -1378.699712
Sum of electronic and thermal Enthalpies= -1378.698768
Sum of electronic and thermal Free Energies= -1378.791890

**Name of compound**	**Int2-1-O4-H-OOH**		
Cartesian Coordinates optimized at B3LYP/6-311G(d,p)			
0 2			
C	-0.96133500	-0.82835900	-0.29752400
C	-2.34892300	-1.19990100	-0.19501700
C	-3.29444900	-0.10826300	0.07892300
C	-2.83801000	1.19011600	0.21597000
C	0.96545000	0.79976800	-0.36591300
C	-1.47165600	1.49468700	0.10158600
C	-0.51939700	0.46787500	-0.15987800
C	0.68617600	4.70367700	-0.05829200
C	2.81536300	2.56362300	-0.29554100
H	2.95005600	2.30916800	-1.35948800
C	0.32106900	3.24571900	-0.35081200
C	1.37235400	2.21938800	0.13471600
H	0.21537800	3.15537000	-1.44062400
H	1.36940700	2.22732500	1.23227700
O	-2.74377100	-2.37709900	-0.33680000
H	-4.59019000	-2.89586900	-0.05461900
H	-3.53252700	1.99777300	0.41320100
C	-1.03420300	2.92049100	0.29130300
H	-1.79693800	3.60180800	-0.08863900
H	-0.96201700	3.12437200	1.37121000
H	1.11313000	0.82827700	-1.45474300
C	-5.61299800	0.41937600	0.37805300
H	-5.50374100	0.91010100	1.35021400
H	-6.52147900	-0.17649800	0.35374800
H	-5.63444700	1.17082900	-0.41734600

O	-4.55877200	-0.51591800	0.16095500
H	-0.27300700	-1.64225600	-0.48911600
C	1.86927100	-0.30973900	0.16342000
C	1.90051400	-0.64281700	1.51927100
C	2.68670100	-1.03272500	-0.72118100
C	2.72675000	-1.66063800	1.99099000
H	1.27093400	-0.10853900	2.22095000
C	3.51186200	-2.05229500	-0.25497200
C	3.53602700	-2.37195000	1.11444600
H	2.75358300	-1.92051500	3.04222400
O	4.33945900	-3.36550900	1.57499500
H	4.80768900	-3.73388900	0.81382800
H	2.66439100	-0.79340600	-1.77700100
C	4.39254700	-2.60170300	-2.42267300
H	3.41303000	-2.77161300	-2.88158900
H	5.10723000	-3.32081200	-2.81867600
H	4.73439300	-1.58648000	-2.64995500
O	4.34781000	-2.82600600	-1.01857200
O	-0.40991500	5.52001400	-0.47208900
O	-6.40947800	-2.67165900	-0.57672200
H	-6.68632500	-3.36634200	-1.18642100
O	-5.40180300	-3.38515400	0.18912700
H	-0.17761300	6.43826800	-0.30884400
O	3.82086600	1.97000400	0.51051800
H	3.71761000	1.01368300	0.46444500
H	2.99278500	3.63397400	-0.20047900
H	0.88497400	4.82554900	1.01785600
H	1.58985300	4.99234300	-0.60497100

Frequency and Energy at B3LYP/6-311G(d,p)in gas phase

Zero-point correction=	0.428649 (Hartree/Particle)
Thermal correction to Energy=	0.458492
Thermal correction to Enthalpy=	0.459437
Thermal correction to Gibbs Free Energy=	0.364261
Sum of electronic and zero-point Energies=	-1378.748516
Sum of electronic and thermal Energies=	-1378.718673
Sum of electronic and thermal Enthalpies=	-1378.717729
Sum of electronic and thermal Free Energies=	-1378.812904

**Name of compound**	**Int1-7-C2-H-OOH**		
Cartesian Coordinates optimized at B3LYP/6-311G(d,p)			
0 2			
O	1.09241700	0.82435300	-1.35714500
O	-1.56265900	-1.01751500	1.43312700
O	6.18687500	0.15337000	1.09767600
O	-6.45900200	-1.38727000	-0.01635200
O	6.74772600	-1.80947200	-0.58557100
O	-7.27508100	1.11327600	-0.27763900
C	0.23918600	-0.99644800	-0.13668100
C	-0.96272400	-0.18526800	-0.67410100
C	1.39609900	0.01452400	-0.18223400
C	-1.76050100	0.13718900	0.61611800

C	-0.33854900	1.00963200	-1.41439300
C	-0.20573700	-1.43963600	1.27443400
C	2.80331400	-0.51145100	-0.28849100
C	-3.23277300	0.39915900	0.42218400
C	3.80437300	0.07981000	0.49690300
C	-4.13026400	-0.67437900	0.32076600
C	3.15291900	-1.52686000	-1.17802100
C	-3.71163700	1.69998600	0.29718700
C	5.12023900	-0.34876400	0.39721100
C	-5.47625200	-0.43387600	0.09042700
C	4.47214200	-1.96450600	-1.27629000
C	-5.06517800	1.94350000	0.05459400
C	5.46089100	-1.38593200	-0.49129500
C	-5.95338400	0.88475200	-0.05160200
C	5.96856400	1.27613100	1.94774000
C	-6.09526900	-2.75380800	0.13371100
H	0.46128500	-1.85172600	-0.77416800
H	-1.58982800	-0.78433700	-1.33547800
H	1.33268900	0.67216700	0.69325700
H	-1.30615900	1.01490600	1.10260900
H	-0.61349000	1.05537600	-2.46808900
H	-0.60438500	1.96239100	-0.94090600
H	-0.18101400	-2.52119900	1.41190600
H	0.42417400	-0.98072100	2.04856600
H	3.54127700	0.89708400	1.15537100
H	-3.75723200	-1.67994600	0.45682000
H	2.40211700	-1.97758400	-1.81575900
H	-3.03244400	2.53971100	0.39830900
H	4.75481200	-2.75266300	-1.96353900
H	-5.44811500	2.95219600	-0.04140800
H	7.27050500	-1.27042100	0.02371400
H	-7.71319200	0.25198500	-0.30044800
H	5.29543400	1.02025900	2.77262500
H	5.55605200	2.12085500	1.38756500
H	6.94501600	1.54290500	2.34792500
H	-5.66866900	-2.94318300	1.12404200
H	-5.37912500	-3.05916900	-0.63648300
H	-7.01541900	-3.32425500	0.01916600
O	2.47229700	3.11263700	-1.15149500
H	2.00703200	2.24953200	-1.34560900
O	2.47316800	3.20103300	0.17377400

Frequency and Energy at B3LYP/6-311G(d,p)in gas phase

Zero-point correction= 0.408927 (Hartree/Particle)
Thermal correction to Energy= 0.436180
Thermal correction to Enthalpy= 0.437124
Thermal correction to Gibbs Free Energy= 0.346222
Sum of electronic and zero-point Energies= -1377.550210
Sum of electronic and thermal Energies= -1377.522958
Sum of electronic and thermal Enthalpies= -1377.522014
Sum of electronic and thermal Free Energies= -1377.612915

Name of compound	TS-7-C2-H-OOH

Cartesian Coordinates optimized at B3LYP/6-311G(d,p)			
0 2			
O	-0.98870500	-2.05353400	-0.78309200
O	1.26850800	1.21860200	0.69507500
O	-5.62479400	1.79912100	0.18818700
O	6.13709000	1.48614700	-0.87213300
O	-7.00854000	-0.10808400	-1.00991800
O	7.25863000	-0.72683800	0.03930500
C	-0.46404600	0.24604100	-0.63654400
C	0.86081900	-0.53366900	-0.79376700
C	-1.46128000	-0.85963400	-0.23874100
C	1.63457900	-0.14852600	0.49409600
C	0.44270900	-2.00246400	-0.93712500
C	-0.12790400	1.33987000	0.40856500
C	-2.91536500	-0.66557000	-0.46739700
C	3.13340500	-0.28401700	0.41072600
C	-3.53876300	0.51700700	-0.01821900
C	3.89866200	0.72272600	-0.19718900
C	-3.69595800	-1.65223800	-1.07760900
C	3.77107400	-1.42169600	0.89669500
C	-4.89968600	0.69980000	-0.19971400
C	5.27197000	0.57425400	-0.31640100
C	-5.06302700	-1.46259000	-1.26328200
C	5.15274000	-1.57861300	0.76587800
C	-5.67555500	-0.29397800	-0.83070200
C	5.91001200	-0.58839400	0.16023100
C	-4.94846800	2.85386700	0.86304500
C	5.60715200	2.71594200	-1.34925200
H	-0.79195100	0.68470500	-1.58290800
H	1.42856500	-0.19088200	-1.65988100
H	-1.35633800	-0.99562300	1.02476000
H	1.25938400	-0.75411400	1.33062000
H	0.67087900	-2.41509600	-1.92101900
H	0.90404200	-2.64290800	-0.17832600
H	-0.29150000	2.34803000	0.02296400
H	-0.70626300	1.20978700	1.32767900
H	-2.95443200	1.27455700	0.48461800
H	3.40086500	1.62109700	-0.53544500
H	-3.22276500	-2.56338700	-1.41617500
H	3.19492900	-2.19241800	1.39692300
H	-5.67324600	-2.21570400	-1.74715500
H	5.65854900	-2.45877000	1.14392300
H	-7.22944500	0.75788600	-0.64081700
H	7.58909900	0.07656400	-0.38437400
H	-4.18050000	3.30284300	0.22449500
H	-4.48988000	2.49918200	1.79170300
H	-5.70792700	3.59876900	1.09361400
H	5.12375400	3.27783600	-0.54340500
H	4.88875100	2.55011700	-2.15915100
H	6.45590300	3.28231500	-1.72915300
O	-2.12238300	-0.25237000	2.89274700
H	-2.93025900	-0.78049200	2.97513600
O	-1.21963400	-1.15591700	2.30502800

Frequency and Energy at B3LYP/6-311G(d,p)in gas phase
Zero-point correction= 0.402950 (Hartree/Particle)<br>Thermal correction to Energy= 0.429946<br>Thermal correction to Enthalpy= 0.430891<br>Thermal correction to Gibbs Free Energy= 0.341956<br>Sum of electronic and zero-point Energies= -1377.525643<br>Sum of electronic and thermal Energies= -1377.498647<br>Sum of electronic and thermal Enthalpies= -1377.497703<br>Sum of electronic and thermal Free Energies= -1377.586637

**Name of compound**	**Int2-7-C2-H-OOH**

Cartesian Coordinates optimized at B3LYP/6-311G(d,p)

```
0 2
 O                 -1.02230900   -1.54468000   -1.00456800
 O                  1.04728000    1.20866000    1.01431100
 O                 -5.77316000    2.03801100    0.45168900
 O                  5.99372100    1.86948900   -0.26343800
 O                 -7.15757400   -0.19562600    0.16890300
 O                  7.05690800   -0.53766300   -0.01604600
 C                 -0.62701100    0.77311300   -0.64040300
 C                  0.70751300    0.06439200   -0.99504600
 C                 -1.63103900   -0.34183100   -0.69388600
 C                  1.42735700   -0.01628100    0.37635700
 C                  0.27580900   -1.27197000   -1.58731200
 C                 -0.34363200    1.38014600    0.77329100
 C                 -3.02008200   -0.31090900   -0.48174800
 C                  2.92583400   -0.14467700    0.31349700
 C                 -3.67016800    0.90701500   -0.11113400
 C                  3.72228900    0.98385800    0.06561100
 C                 -3.82471900   -1.47367200   -0.62512200
 C                  3.53053000   -1.39063100    0.45868700
 C                 -5.03396000    0.93496000    0.09728400
 C                  5.09817400    0.85062900   -0.03924200
 C                 -5.19178200   -1.42606000   -0.40811900
 C                  4.91581400   -1.52594400    0.34049600
 C                 -5.81421100   -0.23497200   -0.04474600
 C                  5.70557500   -0.41462500    0.09055800
 C                 -5.10190700    3.27614300    0.63643600
 C                  5.49366000    3.19487800   -0.37714400
 H                 -0.87662600    1.56850800   -1.35042700
 H                  1.31545000    0.63996700   -1.69409100
 H                 -0.80009100   -3.06525700    0.04946500
 H                  1.01889800   -0.86665000    0.93886200
 H                  0.16019700   -1.21787800   -2.67390200
 H                  0.92627000   -2.10920000   -1.33493000
 H                 -0.55888500    2.44897100    0.82961900
 H                 -0.93380800    0.85708300    1.53620000
 H                 -3.08692200    1.81081400    0.00085100
 H                  3.24737200    1.95264200   -0.00788000
 H                 -3.35899900   -2.40408500   -0.91979100
 H                  2.92272300   -2.26117800    0.68001600
 H                 -5.80546700   -2.31219300   -0.51898600
 H                  5.39863100   -2.48912500    0.45309800
```

H	-7.38125600	0.71411800	0.40686800
H	7.41104300	0.34781200	-0.17297700
H	-4.61123200	3.60255100	-0.28684600
H	-4.36096500	3.20898000	1.44030500
H	-5.86949900	3.99743500	0.91184800
H	4.97768600	3.50338000	0.53804500
H	4.81158800	3.28964500	-1.22897300
H	6.36208600	3.83192100	-0.53695900
O	0.48750600	-3.26761900	1.42112600
H	0.08791900	-3.56243000	2.24980500
O	-0.41492100	-3.86647200	0.45313600

Frequency and Energy at B3LYP/6-311G(d,p)in gas phase

Zero-point correction= 0.407255 (Hartree/Particle)
Thermal correction to Energy= 0.435325
Thermal correction to Enthalpy= 0.436269
Thermal correction to Gibbs Free Energy= 0.344463
Sum of electronic and zero-point Energies= -1377.553750
Sum of electronic and thermal Energies= -1377.525680
Sum of electronic and thermal Enthalpies= -1377.524736
Sum of electronic and thermal Free Energies= -1377.616542

Name of compound	Int1-7-O4'-H-OOH

Cartesian Coordinates optimized at B3LYP/6-311G(d,p)

0 2

O	2.53234800	-2.47260200	-0.72780100
O	-0.26588500	0.04438100	-1.66370800
O	6.19638900	2.28344400	-0.04526500
O	-4.95240400	1.43364300	-0.32507500
O	7.15764100	0.90056500	1.99491700
O	-6.00725500	-0.60955400	1.05332500
C	1.20969100	-0.66907000	0.01852400
C	0.26559500	-1.85073100	-0.34496600
C	2.54680000	-1.04655400	-0.67915300
C	-0.82583100	-1.20920000	-1.24064800
C	1.20293700	-2.84586000	-1.06903400
C	0.50226700	0.55126000	-0.56935500
C	3.79480200	-0.55713500	0.01140000
C	-2.18789600	-1.03568400	-0.57396400
C	4.39679400	0.63900500	-0.40478800
C	-2.90200700	0.15618100	-0.75642100
C	4.34205800	-1.25289000	1.08718700
C	-2.78647200	-2.06960900	0.14390200
C	5.51378700	1.12921200	0.26042800
C	-4.17232800	0.31063000	-0.21514900
C	5.46852500	-0.76696800	1.74971000
C	-4.06439700	-1.92044300	0.68277700
C	6.05761700	0.42389600	1.34901700
C	-4.77015500	-0.73537600	0.51615000
C	5.75121800	3.06462900	-1.14489200
C	-4.45363300	2.53597000	-1.06617700
H	1.36703200	-0.57820000	1.09385200
H	-0.16598200	-2.30230900	0.54620700

H	2.51910200	-0.65050000	-1.70699900
H	-0.96009600	-1.78698700	-2.16119300
H	1.06176700	-3.88316900	-0.76182300
H	1.06188300	-2.78070600	-2.15801100
H	-0.14982800	1.02397800	0.17608500
H	1.18132900	1.30744800	-0.96916800
H	3.99240400	1.17074800	-1.25726300
H	-2.44214000	0.94812300	-1.33112800
H	3.89593800	-2.19067200	1.39201400
H	-2.26896800	-3.01215800	0.28516000
H	5.90776800	-1.30482400	2.58139600
H	-4.53427100	-2.72042400	1.24252500
H	7.40975000	1.72404000	1.55651400
H	-6.37849300	0.26474600	0.84667300
H	4.72862100	3.42474700	-0.98797400
H	5.79850300	2.49720200	-2.08055900
H	6.42797700	3.91551100	-1.20414100
H	-4.26300300	2.26320100	-2.11002000
H	-3.53313800	2.93240300	-0.62335100
H	-5.23083800	3.29773000	-1.02685500
O	-8.83165700	1.34823100	1.48019200
H	-9.44630900	2.10363300	1.41677500
O	-7.74358300	1.72549900	0.82266500

Frequency and Energy at B3LYP/6-311G(d,p)in gas phase

Zero-point correction= 0.407629 (Hartree/Particle)
Thermal correction to Energy= 0.435425
Thermal correction to Enthalpy= 0.436370
Thermal correction to Gibbs Free Energy= 0.342655
Sum of electronic and zero-point Energies= -1377.538522
Sum of electronic and thermal Energies= -1377.510726
Sum of electronic and thermal Enthalpies= -1377.509782
Sum of electronic and thermal Free Energies= -1377.603497

**Name of compound**	**TS-7-O4’-H-OOH**

Cartesian Coordinates optimized at B3LYP/6-311G(d,p)

0 2

O	2.43567600	-2.47310800	-0.72068200
O	-0.29604600	0.12868800	-1.66726200
O	6.24835100	2.16030300	-0.06201900
O	-4.90748500	1.58864400	-0.22975200
O	7.13712500	0.78010400	2.01180900
O	-6.04720600	-0.53244200	0.96245800
C	1.15664000	-0.63705300	0.01588600
C	0.18405000	-1.79133200	-0.35758300
C	2.48733800	-1.04741600	-0.67550900
C	-0.87773800	-1.11822000	-1.26863300
C	1.10151600	-2.81340700	-1.07184400
C	0.48683800	0.60564700	-0.56738600
C	3.74387400	-0.58859300	0.01980300
C	-2.24890500	-0.93914900	-0.62973700
C	4.39270000	0.57746300	-0.41135000
C	-2.91055000	0.28695300	-0.72419900

C	4.25416200	-1.28435000	1.11365100
C	-2.90120000	-2.02177900	-0.00984400
C	5.52054500	1.03841000	0.25660200
C	-4.18695400	0.44791600	-0.19403800
C	5.39087100	-0.82796200	1.77923100
C	-4.16954900	-1.87981800	0.51589700
C	6.02723200	0.33315200	1.36333700
C	-4.85876000	-0.64556400	0.45259200
C	5.84707300	2.93586100	-1.18270100
C	-4.36247400	2.74145700	-0.86045600
H	1.31070100	-0.55640400	1.09241100
H	-0.27220500	-2.23060000	0.52792000
H	2.47431900	-0.65312900	-1.70426500
H	-1.00811800	-1.68954900	-2.19470400
H	0.93070500	-3.84567500	-0.76300800
H	0.96999900	-2.74750300	-2.16208700
H	-0.15205200	1.09496600	0.17868800
H	1.18852700	1.34275400	-0.96280500
H	4.01650600	1.10807900	-1.27745900
H	-2.41091100	1.10244100	-1.22801200
H	3.77201000	-2.20013100	1.43050500
H	-2.40863600	-2.98616600	0.05249600
H	5.80216200	-1.36629400	2.62470600
H	-4.68123000	-2.70514600	0.99578400
H	7.42592400	1.58471000	1.56101400
H	-6.62713700	0.46597800	0.89555200
H	4.83775100	3.33888800	-1.04569500
H	5.88341100	2.34810400	-2.10616400
H	6.55727200	3.75831500	-1.25003800
H	-4.15984200	2.55283300	-1.91952500
H	-3.44235400	3.06495700	-0.36325700
H	-5.12344500	3.51267400	-0.76300500
O	-8.51264000	1.07709500	1.52322300
H	-9.05301300	1.87723400	1.46039600
O	-7.34409800	1.47013700	0.87450200

Frequency and Energy at B3LYP/6-311G(d,p)in gas phase

Zero-point correction= 0.402928 (Hartree/Particle)
Thermal correction to Energy= 0.429725
Thermal correction to Enthalpy= 0.430669
Thermal correction to Gibbs Free Energy= 0.340755
Sum of electronic and zero-point Energies= -1377.529333
Sum of electronic and thermal Energies= -1377.502536
Sum of electronic and thermal Enthalpies= -1377.501591
Sum of electronic and thermal Free Energies= -1377.591505

**Name of compound**	**Int2-7-O4’-H-OOH**

Cartesian Coordinates optimized at B3LYP/6-311G(d,p)

0 2

O	-2.45021800	2.52522400	-0.53170600
O	0.33992400	0.01013900	-1.59271600
O	-6.15611000	-2.23244200	-0.30214100
O	4.97213200	-1.32466600	-0.11425000

O	-7.11508400	-1.04548800	1.85996000
O	6.06863000	0.82142100	0.97008500
C	-1.14167500	0.67899300	0.10743300
C	-0.18664700	1.86579100	-0.20005600
C	-2.46825500	1.09870300	-0.58436600
C	0.87920900	1.25947500	-1.15663200
C	-1.12226700	2.91813100	-0.84698100
C	-0.44307400	-0.52495700	-0.51822200
C	-3.72519600	0.56006300	0.04888400
C	2.26657700	1.11569400	-0.55047300
C	-4.33725800	-0.58201000	-0.48713500
C	2.94409700	-0.10313400	-0.62278400
C	-4.27100000	1.15280700	1.18547900
C	2.90314500	2.24849000	0.02497600
C	-5.46407300	-1.12302300	0.11962300
C	4.23083800	-0.22165000	-0.11046700
C	-5.40702200	0.61675000	1.79003900
C	4.17054900	2.16496500	0.53357300
C	-6.00682500	-0.52187400	1.27005600
C	4.91456000	0.92694700	0.50645000
C	-5.71474900	-2.90559600	-1.47335000
C	4.47034100	-2.54448200	-0.66047000
H	-1.31067000	0.54910600	1.17684900
H	0.27103000	2.25693000	0.70739500
H	-2.42215800	0.77596200	-1.63688300
H	0.97792800	1.87388400	-2.05949400
H	-0.97521100	3.92875200	-0.46367300
H	-0.98225200	2.93288200	-1.93805400
H	0.19964900	-1.03152700	0.21287200
H	-1.12608600	-1.25945600	-0.94891300
H	-3.93365900	-1.03128200	-1.38613300
H	2.45106500	-0.93955200	-1.09840200
H	-3.81872700	2.05142200	1.58514800
H	2.37527900	3.19602700	0.05460600
H	-5.84631400	1.07511100	2.66791600
H	4.67062500	3.01886100	0.97453000
H	-7.37746500	-1.81774100	1.34145500
H	7.13648900	-0.79171800	0.75011300
H	-4.69770300	-3.29290900	-1.34884600
H	-5.75053300	-2.24647700	-2.34728100
H	-6.40173600	-3.73734600	-1.61959300
H	4.26337500	-2.43385500	-1.72889500
H	3.56305800	-2.85846800	-0.13577400
H	5.26640600	-3.26624600	-0.49902400
O	7.29868300	-2.66116200	1.07673100
H	7.88374700	-2.84113700	1.82263300
O	7.87156900	-1.41699200	0.59203400

Frequency and Energy at B3LYP/6-311G(d,p)in gas phase

Zero-point correction= 0.407590 (Hartree/Particle)
Thermal correction to Energy= 0.435298
Thermal correction to Enthalpy= 0.436242
Thermal correction to Gibbs Free Energy= 0.344218
Sum of electronic and zero-point Energies= -1377.548565

Sum of electronic and thermal Energies=	-1377.520857
Sum of electronic and thermal Enthalpies=	-1377.519913
Sum of electronic and thermal Free Energies=	-1377.611937

Name of compound	Int1-9-O4'-H-OOH		
Cartesian Coordinates optimized at B3LYP/6-311G(d,p)			
0 2			
C	1.38530800	1.40110200	-0.15820500
H	1.89854100	1.59382100	0.79420800
C	0.29734800	-0.54192400	0.60075100
H	-0.53274100	-1.23958900	0.48475100
H	0.62785800	-0.56865500	1.64856800
C	2.05728700	0.15819500	-0.80149800
O	1.36614400	-0.97551900	-0.25650100
H	2.53677000	2.92410900	-1.16420300
H	1.02976500	2.51669100	-1.98441200
O	0.77793100	3.76751500	-0.42435900
C	1.48179900	2.66828900	-1.00191400
C	3.55170300	0.05970000	-0.57001500
C	4.05166800	-0.63162900	0.54198200
C	4.44635600	0.67671400	-1.43671500
C	5.41726400	-0.69187700	0.79955500
H	3.34915900	-1.15143000	1.17759700
C	5.81975500	0.62841100	-1.18275800
H	4.08767300	1.18911700	-2.32278700
C	6.31674400	-0.04295600	-0.07475700
H	6.51548200	1.11077200	-1.86400000
O	7.64727500	-0.12917700	0.22040000
C	5.13714000	-2.03585800	2.74945600
H	5.79459900	-2.47896000	3.49570700
H	4.57336800	-2.82866900	2.24523300
H	4.43599700	-1.35337900	3.24352200
O	5.98870600	-1.34604600	1.84743000
H	1.87939400	0.17148400	-1.88605300
C	-1.03363300	0.96647500	-1.00450900
H	-1.11599100	2.01339700	-1.30753000
H	-0.63354400	0.41469700	-1.86128600
C	-0.03715600	0.88914000	0.17629300
H	-0.45596300	1.45289600	1.01360500
C	-2.41432800	0.44244300	-0.67068600
C	-3.31822600	1.21715500	0.05041900
C	-2.81309600	-0.84284300	-1.07172500
C	-4.58434900	0.72353100	0.37129900
H	-3.04445200	2.22026000	0.36059700
C	-4.07395400	-1.34652000	-0.76063400
H	-2.12136300	-1.44754000	-1.64451000
C	-4.97899700	-0.55111200	-0.02254200
H	-5.28132800	1.34032400	0.92932400
H	1.18744000	3.96557400	0.42383200
O	-6.19615500	-1.08459700	0.25409100
H	-6.73498700	-0.44358300	0.74213900
C	-3.67728000	-3.41955100	-1.87360300

H	-4.24101000	-4.33437800	-2.05019300
H	-2.75984700	-3.66117400	-1.32391800
H	-3.41094800	-2.96779700	-2.83648500
O	-4.53374500	-2.57817700	-1.12018200
O	-8.10901200	0.61258000	1.68603500
H	8.14179700	0.33994600	-0.45917500
O	-7.63374400	1.80715000	2.01018000
H	-8.37297400	2.27093000	2.44755900

Frequency and Energy at B3LYP/6-311G(d,p)in gas phase

Zero-point correction= 0.428125 (Hartree/Particle)
Thermal correction to Energy= 0.457869
Thermal correction to Enthalpy= 0.458813
Thermal correction to Gibbs Free Energy= 0.360871
Sum of electronic and zero-point Energies= -1378.719284
Sum of electronic and thermal Energies= -1378.689539
Sum of electronic and thermal Enthalpies= -1378.688595
Sum of electronic and thermal Free Energies= -1378.786538

Name of compound	TS-9-O4'-H-OOH

Cartesian Coordinates optimized at B3LYP/6-311G(d,p)

0 2

C	1.38100100	1.36857900	0.04735400
H	1.93769300	1.45382900	0.99060700
C	0.33453500	-0.65873600	0.62679800
H	-0.49828500	-1.34549400	0.46854700
H	0.71010100	-0.80494600	1.64891500
C	2.02334300	0.21446300	-0.76721500
O	1.36267700	-0.98125500	-0.32185200
H	2.47245700	3.01990100	-0.81388600
H	0.94767600	2.67617100	-1.62807000
O	0.72208900	3.73496400	0.07142300
C	1.42841800	2.72545000	-0.64841800
C	3.52705700	0.10334000	-0.62492800
C	4.08553900	-0.66503600	0.40609700
C	4.37327700	0.78459900	-1.49151000
C	5.46282200	-0.74029400	0.58390100
H	3.41963600	-1.23231200	1.04059200
C	5.75857700	0.72208400	-1.31668200
H	3.96712000	1.35935900	-2.31688000
C	6.31421500	-0.02660400	-0.28910100
H	6.41652000	1.25478900	-1.99780000
O	7.65761200	-0.13103500	-0.07324100
C	5.28993800	-2.22476600	2.44251000
H	5.98819000	-2.72290000	3.11299000
H	4.69517200	-2.97726900	1.91285000
H	4.62171200	-1.58231200	3.02752100
O	6.09007200	-1.46726600	1.54744900
H	1.79020900	0.34980200	-1.83276200
C	-1.07027000	1.01654800	-0.73157700
H	-1.15521500	2.08996000	-0.92029700
H	-0.71904000	0.54765200	-1.65567600
C	-0.02145900	0.81025500	0.38944000

H	-0.40097900	1.27118300	1.30434300
C	-2.43549300	0.47676500	-0.37908500
C	-3.26764700	1.17864900	0.50946700
C	-2.89026500	-0.73592400	-0.91545900
C	-4.50697200	0.68309600	0.86876300
H	-2.93010300	2.12460100	0.91939300
C	-4.14187400	-1.24709600	-0.58327000
H	-2.25435500	-1.26974600	-1.60994800
C	-4.98625200	-0.53747100	0.33827400
H	-5.14876400	1.22738300	1.54885900
H	1.17035100	3.86787100	0.91272000
O	-6.13755300	-1.06513400	0.65973100
H	-6.94706500	-0.36131800	0.94055500
C	-3.88924600	-3.16765900	-1.97507400
H	-4.50296500	-4.03254600	-2.21847600
H	-2.95055600	-3.50208700	-1.52081800
H	-3.67263200	-2.60489000	-2.88936100
O	-4.66022100	-2.39593900	-1.06198400
O	-7.99076400	0.38776300	0.98472100
H	8.11687800	0.38725000	-0.74183200
O	-7.49422600	1.68699700	1.05765600
H	-8.08670900	2.17186500	0.46496700

Frequency and Energy at B3LYP/6-311G(d,p)in gas phase

Zero-point correction= 0.423831 (Hartree/Particle)
Thermal correction to Energy= 0.452460
Thermal correction to Enthalpy= 0.453404
Thermal correction to Gibbs Free Energy= 0.359512
Sum of electronic and zero-point Energies= -1378.714829
Sum of electronic and thermal Energies= -1378.686200
Sum of electronic and thermal Enthalpies= -1378.685256
Sum of electronic and thermal Free Energies= -1378.779148

**Name of compound**	**Int2-9-O4’-H-OOH**

Cartesian Coordinates optimized at B3LYP/6-311G(d,p)

0 2

C	1.37181600	1.36854700	-0.17826500
H	1.87429800	1.55228000	0.78077900
C	0.29476100	-0.59909100	0.53441000
H	-0.53093200	-1.30128300	0.40884200
H	0.62704400	-0.64399300	1.58067800
C	2.05970900	0.14304500	-0.83840100
O	1.35789100	-1.00696800	-0.33746900
H	2.50621000	2.93905300	-1.13033700
H	1.04175300	2.49440900	-2.00283900
O	0.69635500	3.71636300	-0.43805300
C	1.45567600	2.64929200	-1.00375200
C	3.54930600	0.04488400	-0.58143900
C	4.03259700	-0.65251000	0.53438300
C	4.45485000	0.67217900	-1.42873700
C	5.39399900	-0.70929000	0.81390800
H	3.32361800	-1.18019200	1.15623900
C	5.82418100	0.62722500	-1.15281900

H	4.10853700	1.19018000	-2.31657600
C	6.30532400	-0.05048700	-0.04178700
H	6.52888700	1.11703900	-1.81909000
O	7.62947800	-0.13474700	0.27544300
C	5.08977600	-2.06236800	2.75441000
H	5.73873800	-2.50543700	3.50783700
H	4.53561000	-2.85560300	2.24037400
H	4.38074000	-1.38286300	3.24113100
O	5.95127000	-1.36731300	1.86530700
H	1.90257800	0.18090600	-1.92528200
C	-1.03463500	0.93559300	-1.04805400
H	-1.10465300	1.98616800	-1.34273800
H	-0.63984300	0.37671500	-1.90127800
C	-0.04719400	0.84014300	0.14301700
H	-0.47210200	1.38512900	0.98904600
C	-2.41617700	0.43790500	-0.71100400
C	-3.27813700	1.23886600	0.08453800
C	-2.85946400	-0.81356700	-1.16093900
C	-4.52955300	0.80968300	0.43267200
H	-2.93027100	2.21375600	0.41031900
C	-4.12548800	-1.28339800	-0.83207200
H	-2.19654200	-1.40942100	-1.77553000
C	-5.03016700	-0.47652900	0.00264300
H	-5.20369400	1.41351600	1.03076000
H	1.09709300	3.95361100	0.40427700
O	-6.16600500	-0.90233200	0.30495600
H	-7.25970000	-0.07303100	1.47622500
C	-3.85097200	-3.32676800	-2.02858900
H	-4.47269400	-4.20118900	-2.20657200
H	-2.93374200	-3.62631700	-1.51234200
H	-3.60042600	-2.85287900	-2.98263500
O	-4.63734900	-2.45770200	-1.21554800
O	-7.82814600	0.39767000	2.12419200
H	8.13606400	0.34203300	-0.38988400
O	-7.30221400	1.74752800	2.01597200
H	-8.02806400	2.18731600	1.55546300

Frequency and Energy at B3LYP/6-311G(d,p)in gas phase	
Zero-point correction=	0.428622 (Hartree/Particle)
Thermal correction to Energy=	0.458080
Thermal correction to Enthalpy=	0.459024
Thermal correction to Gibbs Free Energy=	0.362543
Sum of electronic and zero-point Energies=	-1378.734430
Sum of electronic and thermal Energies=	-1378.704972
Sum of electronic and thermal Enthalpies=	-1378.704028
Sum of electronic and thermal Free Energies=	-1378.800509

Figure S1: IRC plots for all transition states related to the reaction of HOO• radical with cyclolariciresinol **1**, pinoresinol **7** and lariciresinol **9** at B3LYP/6-311G(d,p) level of theory in the gas phase.

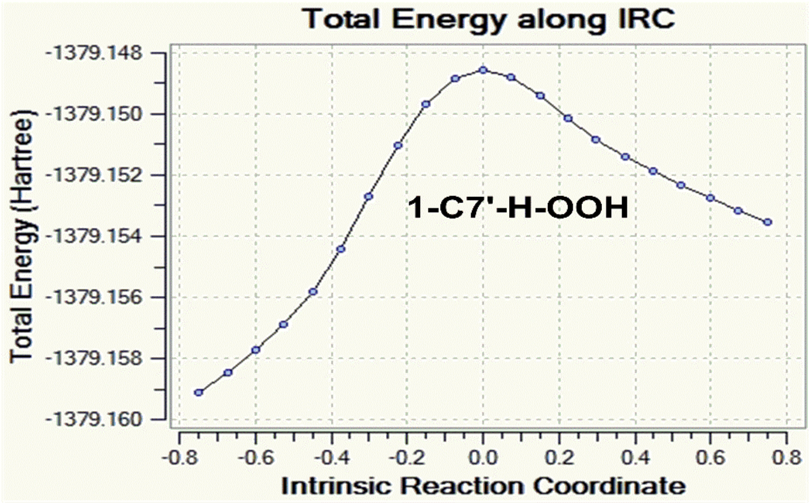

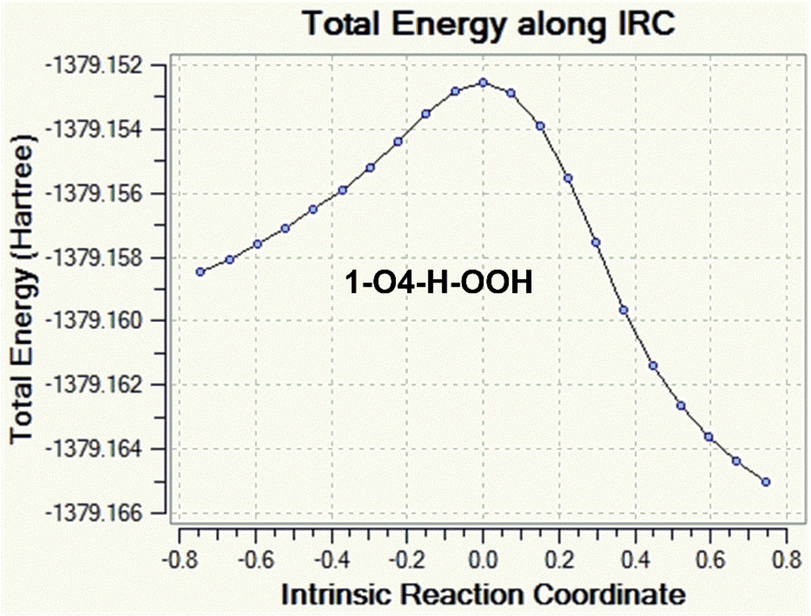

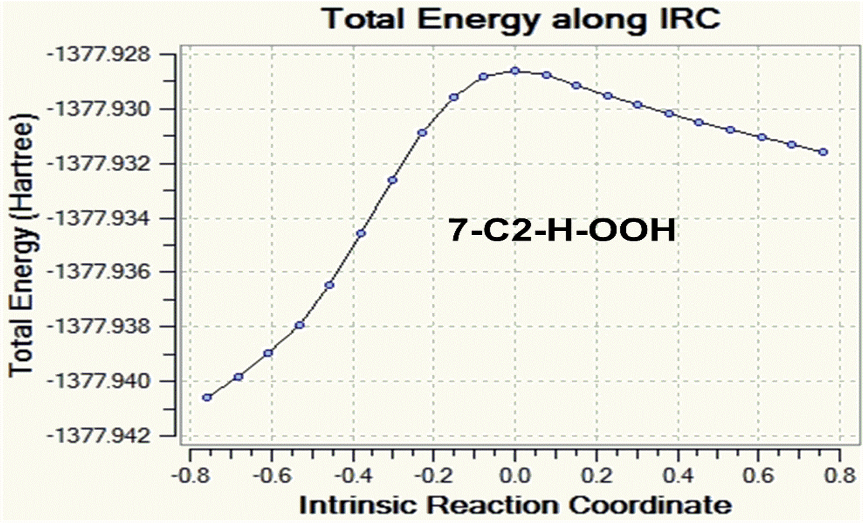
Total Energy along IRC
Total Energy (Hartree)
-1377.928
-1377.930
-1377.932
-1377.934
-1377.936
-1377.938
-1377.940
-1377.942
7-C2-H-OOH
-0.8
-0.6
-0.4
-0.2
0.0
0.2
0.4
0.6
0.8
Intrinsic Reaction Coordinate

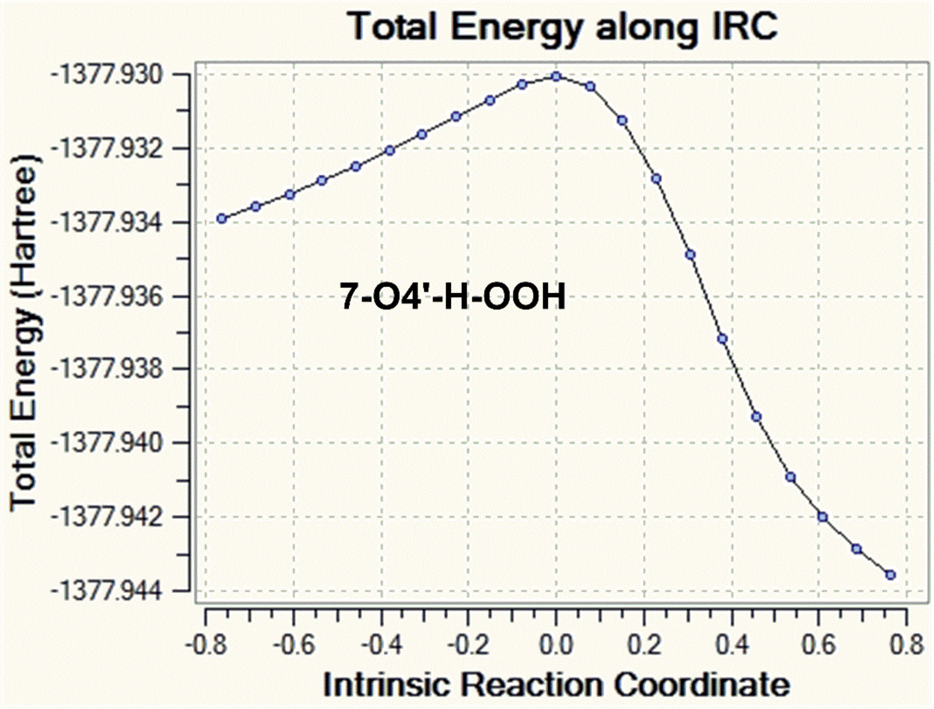
Total Energy along IRC
Total Energy (Hartree)
-1377.930
-1377.932
-1377.934
-1377.936
-1377.938
-1377.940
-1377.942
-1377.944
7-O4'-H-OOH
-0.8
-0.6
-0.4
-0.2
0.0
0.2
0.4
0.6
0.8
Intrinsic Reaction Coordinate

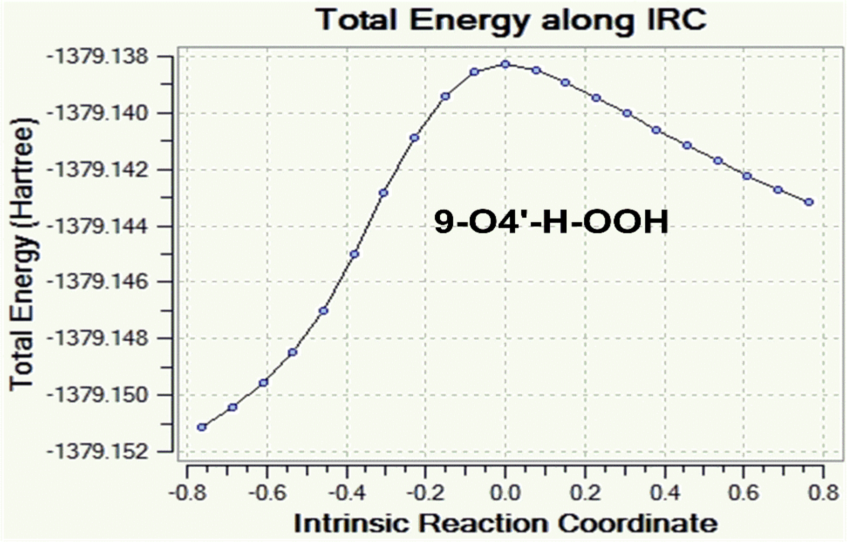
Total Energy along IRC
Total Energy (Hartree)
-1379.138
-1379.140
-1379.142
-1379.144
-1379.146
-1379.148
-1379.150
-1379.152
9-O4'-H-OOH
-0.8
-0.6
-0.4
-0.2
0.0
0.2
0.4
0.6
0.8
Intrinsic Reaction Coordinate

Figure S2. Optimized geometries of TS for the H abstraction channel of reaction between the selected phenolic compounds and the HOO• radical at the B3LYP/6-311G(d,p) level of theory (distances are given in angstroms)

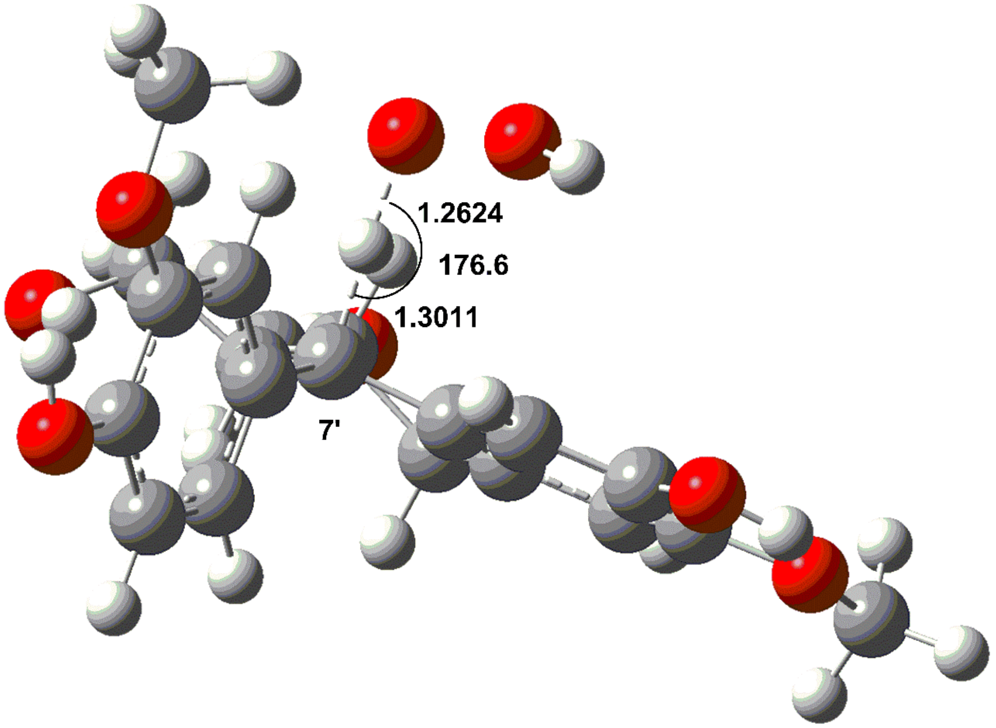

TS-1-C7'-H--OOH

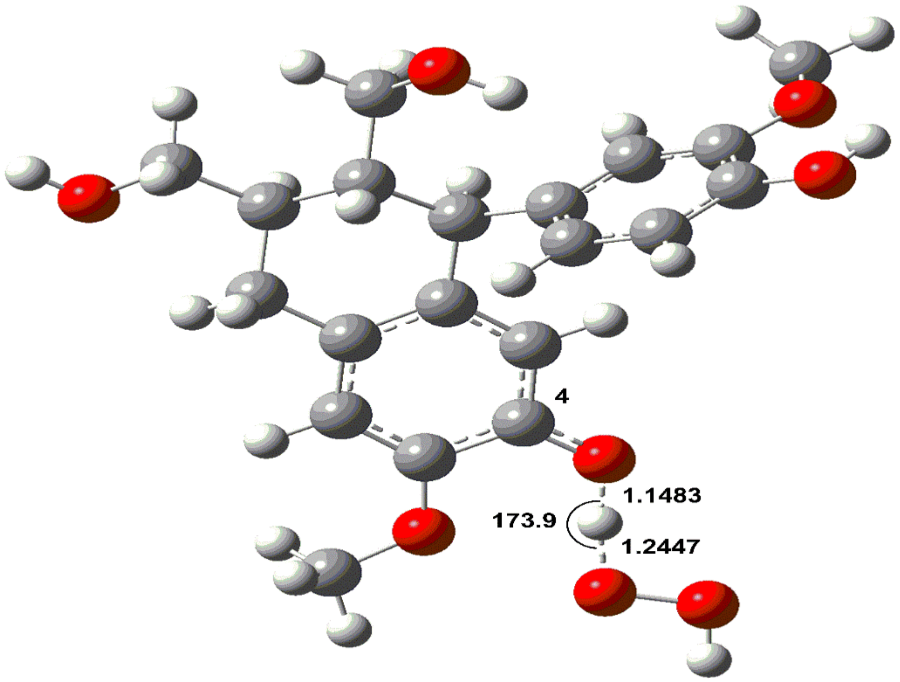

TS-1-O4-H--OOH

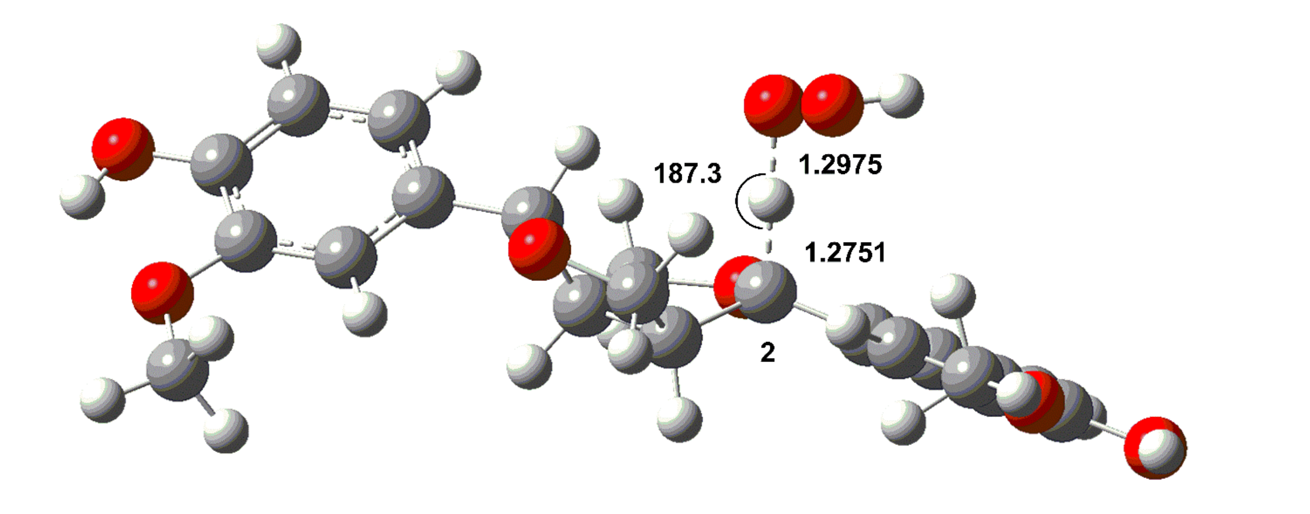

TS-7-C2-H-OOH

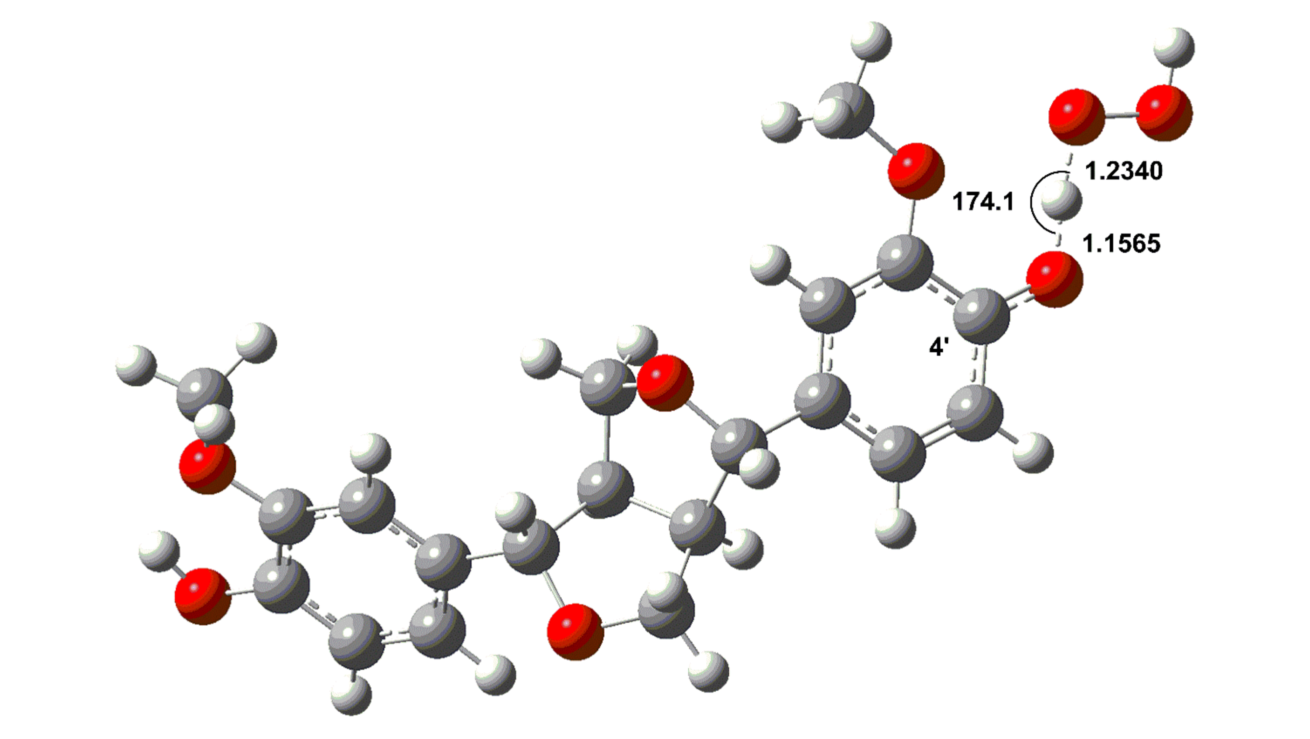

TS-7-O4’-H-OOH

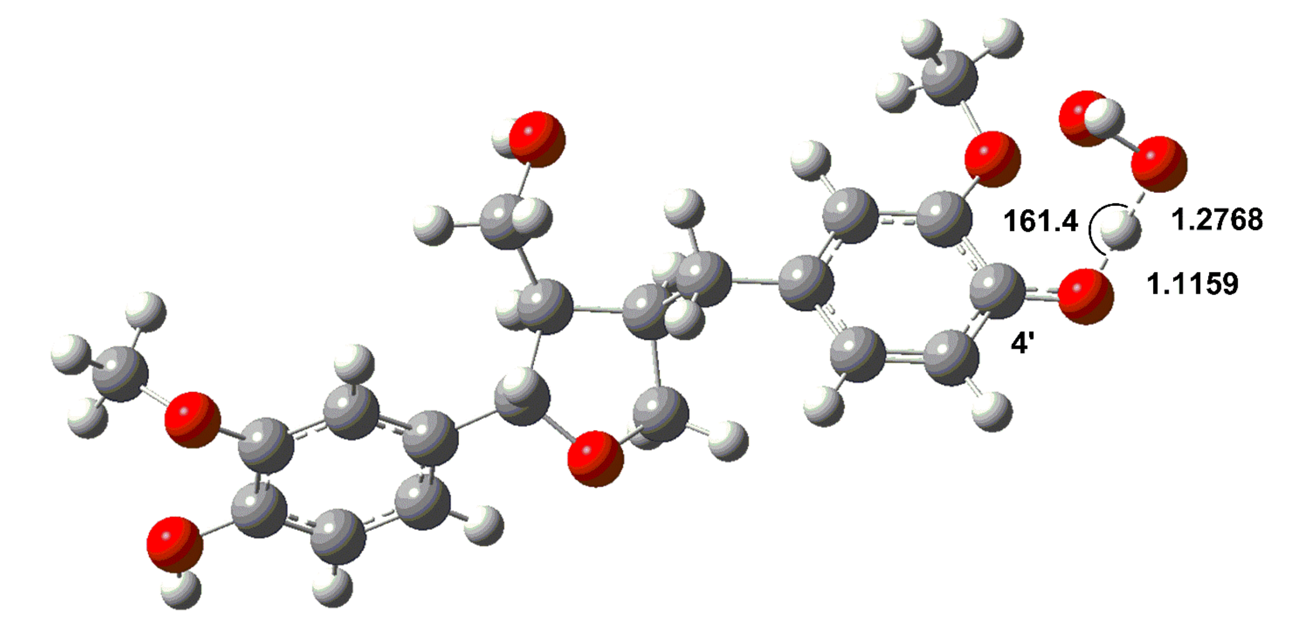

TS-9-O4’-H-OOH
